# A novel antiviral lncRNA, EDAL, shields a T309 *O*-GlcNAcylation site to promote EZH2 lysosomal degradation

**DOI:** 10.1186/s13059-020-02150-9

**Published:** 2020-09-01

**Authors:** Baokun Sui, Dong Chen, Wei Liu, Qiong Wu, Bin Tian, Yingying Li, Jing Hou, Shiyong Liu, Juan Xie, Hao Jiang, Zhaochen Luo, Lei Lv, Fei Huang, Ruiming Li, Chengguang Zhang, Yuling Tian, Min Cui, Ming Zhou, Huanchun Chen, Zhen F. Fu, Yi Zhang, Ling Zhao

**Affiliations:** 1 State Key Laboratory of Agricultural Microbiology, Huazhong Agricultural University, Wuhan, 430070 China; 2 Key Laboratory of Preventive Veterinary Medicine of Hubei Province, College of Veterinary Medicine, Huazhong Agricultural University, Wuhan, 430070 China; 3 Center for Genome analysis, ABLife Inc., Wuhan, 430075 China; 4 Center for Genome analysis and Laboratory for Genome Regulation and Human Health, ABLife Inc., Wuhan, 430075 China; 5 School of Physics, Huazhong University of Science and Technology, Wuhan, 430074 China; 6 Key Laboratory of Marine Drugs, Ministry of Education, School of Medicine and Pharmacy, Shandong Provincial Key Laboratory of Glycoscience and Glycotechnology, Ocean University of China, Qingdao, 266003 China; 7 Department of Pathology, University of Georgia, Athens, GA 30602 USA

**Keywords:** EZH2, lncRNA, neurotropic virus, *O*-GlcNAcylation, PCP4L1

## Abstract

**Background:**

The central nervous system (CNS) is vulnerable to viral infection, yet few host factors in the CNS are known to defend against invasion by neurotropic viruses. Long noncoding RNAs (lncRNAs) have been revealed to play critical roles in a wide variety of biological processes and are highly abundant in the mammalian brain, but their roles in defending against invasion of pathogens into the CNS remain unclear.

**Results:**

We report here that multiple neurotropic viruses, including rabies virus, vesicular stomatitis virus, Semliki Forest virus, and herpes simplex virus 1, elicit the neuronal expression of a host-encoded lncRNA EDAL. EDAL inhibits the replication of these neurotropic viruses in neuronal cells and rabies virus infection in mouse brains. EDAL binds to the conserved histone methyltransferase enhancer of zest homolog 2 (EZH2) and specifically causes EZH2 degradation via lysosomes, reducing the cellular H3K27me3 level. The antiviral function of EDAL resides in a 56-nt antiviral substructure through which its 18-nt helix-loop intimately contacts multiple EZH2 sites surrounding T309, a known *O*-GlcNAcylation site. EDAL positively regulates the transcription of Pcp4l1 encoding a 10-kDa peptide, which inhibits the replication of multiple neurotropic viruses.

**Conclusions:**

Our findings show that a neuronal lncRNA can exert an effective antiviral function via blocking a specific *O*-GlcNAcylation that determines EZH2 lysosomal degradation, rather than the traditional interferon-dependent pathway.

## Backgrounds

Among infectious diseases of the CNS, those caused by viral pathogens—known as neurotropic viruses—are far more common than bacteria, fungi, and protozoans [1, 2]. Neurotropic viruses arrive to the CNS through multiple routes and propagate within various cell types including astrocytes, microglia and neurons, depending on the entering routes and virus types [3]. Infection of some neurotropic viruses can cause meningitis or encephalitis and result in severe neurologic dysfunction, such as vesicular stomatitis virus (VSV), Semliki Forest virus (SFV), herpes simplex virus 1 (HSV-1), and human immunodeficiency virus (HIV) [4–6]. Moreover, nearly half of all emerging viruses are neurotropic viruses [7], including the Dengue and Zika viruses [8, 9]. Rabies virus (RABV) is a typical neurotropic virus and is the causative agent of rabies, a globally well-known and often lethal encephalitis. Therefore, it is urgent to develop new approaches for therapies as well as for cheaper and more effective vaccines against rabies [10, 11].

LncRNAs are involved in the development, plasticity, and pathology of the nervous system [12–15]. Notably, around 40% of lncRNAs detected to date are expressed specifically in the brain [16]. Genome-wide association studies (GWASs) and functional studies have associated lncRNAs with neurological diseases including autism spectrum disorders (ASD), schizophrenia, Alzheimer’s disease, and neuropathic pain, among others [13]. Mechanistically, it has been shown that lncRNAs can regulate chromatin modifications and gene expression, at both the transcriptional and the post-transcriptional levels [17–20]. LncRNAs have recently been shown to regulate innate immune responses by either promoting or inhibiting viral genome replication, highlighting them as a class of novel targets for developing antiviral therapies [21–27]. It is conceivable that antiviral lncRNAs targeting none-innate immune response pathway may exist in neuron cells and brains, which has not been documented yet.

Polycomb repressive complex 2 (PRC2) is a protein complex with an epigenetic regulator function in maintaining the histone modifications that mark transcriptional repression states which are established during early developmental stages [28]. Some lncRNAs are known to interact with and direct PRC2 toward the chromatin sites of action, thusly defining a trans-acting lncRNA mechanism [29, 30]. The EZH2 methyltransferase enzyme is the catalytic component of PRC2: it binds RNAs and catalyzes di- or tri-methylation of histone H3 lysine 27 (H3K27me2/3), a modification which leads to the formation of facultative heterochromatin and thus to transcriptional repression [31–33]. Many cancers are known to feature very high EZH2 expression levels, so this protein has emerged as an anticancer target for which multiple chemical inhibitors have been developed [34, 35]. It has also been recently reported that inhibitors of the histone methyltransferase activity of EZH2 can suppress infection by several viruses, suggesting a function of EZH2 and/or PRC2 in regulating viral infection [36]. However, it is unclear how this regulation occurs. In general, PRC2 (EZH2) binds different classes of RNAs in a promiscuous manner in vitro and in cells, and some lncRNAs such as RepA RNA show in vitro specificity with PRC2 [37, 38]. The specificity of PRC2 (EZH2) interaction with lncRNAs is expected for at least some of its regulation and biological function in living cells, which require further studies [39].

Biochemical studies have established that post-translational modifications (PTM) of EZH2, including phosphorylation and *O*-GlcNAcylation, can regulate its stability [40–42]. NIMA-related kinase (NEK2) was recently shown to phosphorylate EZH2, which protects EZH2 from ubiquitin-dependent proteasome degradation, thereby promoting glioblastoma growth and radio-resistance [43]. LncRNAs have been shown to regulate the stability of proteins such as ZMYND8 and CARM1, expanding the scope of their known regulatory functions [44, 45]. It was recently reported that a newly identified lncRNA (ANCR) increases the phosphorylation-mediated stability of EZH2 by promoting its interaction with the well-known kinase CDK1 [46]. However, it remains unclear how lncRNA interacts with proteins to regulate their stability.

Here, we report our discovery of a novel virus-inducible lncRNA (EZH2 degradation-associated lncRNA, EDAL) that we identified via deep RNA-seq of RABV-infected Neuro-2a (N2a) cells. EDAL can inhibit the replication of multiple neurotropic viruses in neuronal cells, including two negative-strand RNA viruses-RABV and VSV, a positive-strand RNA virus-SFV, and a DNA virus-HSV-1, as well as RABV infection in mice. We found that increased EDAL levels reduce the cellular level of EZH2 and of its enzymatic product H3K27me3 epigenetic marks. Mutational analysis, structural prediction, and molecular simulations revealed that a 56-nt functional substructure of EDAL, wherein a helical-loop intimately contacts EZH2 T309 and the surrounding regions. This protein-lncRNA interaction prevents T309 from receiving a previously demonstrated *O*-GlcNAcylation PTM that is known to increase EZH2’s cellular stability. We further show that *Pcp4l1* is a EDAL-regulated gene which encodes a small peptide suppressing RABV, VSV, SFV, and HSV-1 infection. Thus, our study reveals a previously unknown lncRNA-PTM-mediated link between host antiviral responses and epigenetic regulation.

## Results

### Identification of a host lncRNA induced by viral infection

**Fig. 1:**
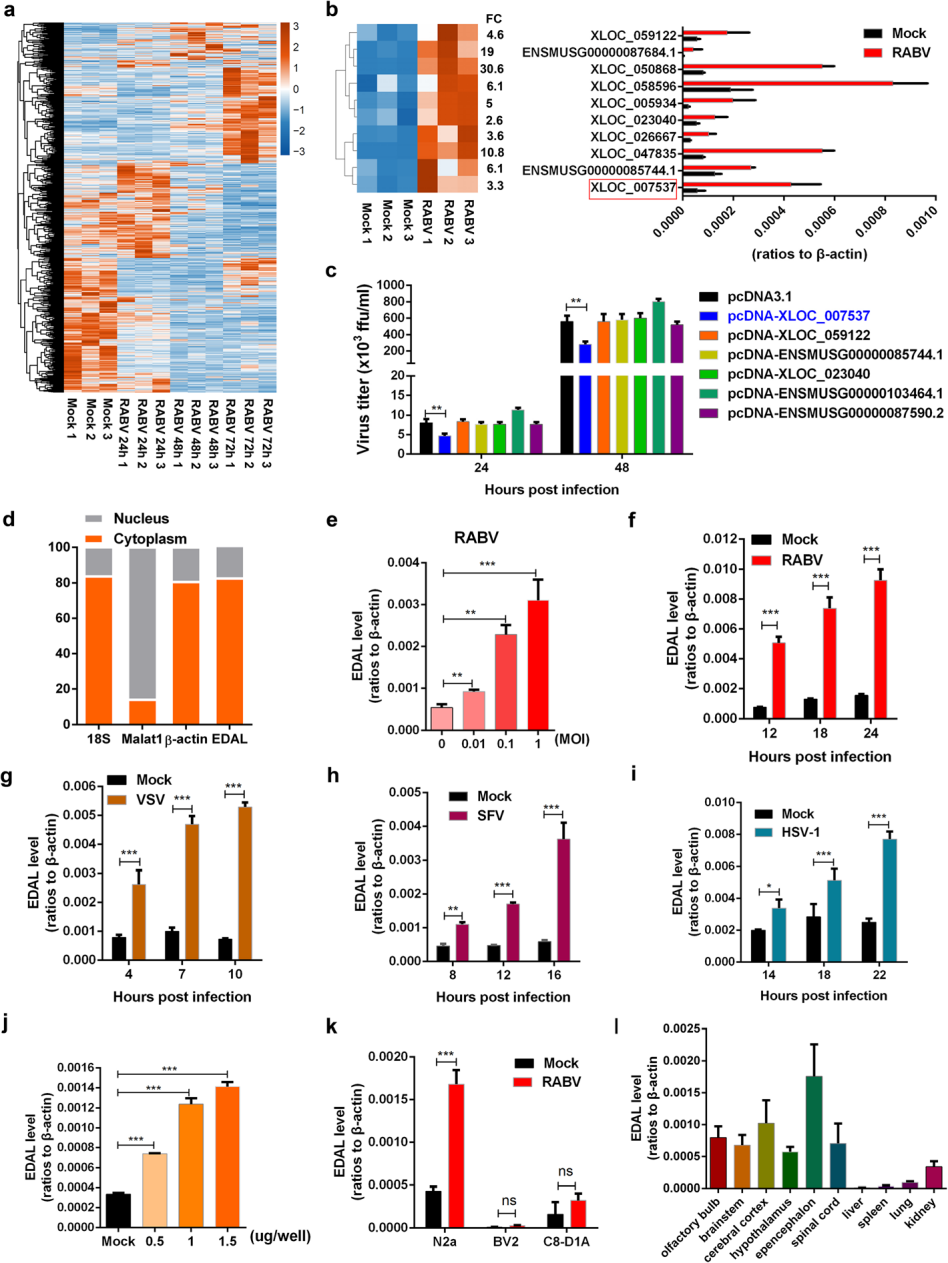
LncRNA EDAL is upregulated after viral infection. **a** Total 1434 differentially expressed lncRNAs were identified by RNA-seq analysis in RABV-infected N2a cells compared with mock-infected cells (*n* = 3; 2 fold change (FC) and 0.01 *p* value). These lncRNAs were clustered and shown by heatmap. **b** Ten of the differentially expressed lncRNAs were selected and clustered in a heatmap (left); the corresponding express level were confirmed by qPCR (right). **c** The indicated upregulated lncRNAs were selected and expressed in N2a cells. At 12 h post transfection, the cells were infected with RABV at MOI 0.01 and virus titers in supernatants were measured at indicated time point. **d** Cytosol and nuclear fractions from N2a cells were extracted. Subcellular localization of EDAL was determined by qPCR. 18S ribosomal RNA (18S) and β-actin were included as cytoplasmic RNA markers, while lncRNA Malat1 was used as a nuclear marker. **e** N2a cells were infected with RABV at different MOIs for 24 h and EDAL level was analyzed by qPCR. **f**–**i** N2a cells were infected with RABV (**f**), VSV (**g**), SFV (**h**), or HSV-1 (**i**) at MOI 1 and at indicated time points post infection. EDAL levels were determined by qPCR. **j** N2a cells were transfected with RABV genomic RNA at different doses for 24 h, and EDAL level was analyzed by qPCR. **k** The basal or induced level of EDAL (infected with RABV at MOI 1 for 24 h) in different cell lines were determined by qPCR. **l** The basal level of EDAL in different tissues was analyzed by qPCR. Statistical analysis of grouped comparisons was carried out by Student’s *t* test (*P < 0.05; **P < 0.01; ***P < 0.001). Bar graph represents means ± SD, *n* = 3

We conducted a time-course RNA-seq analysis of cultured N2a cells that were infected with pathogenic RABV (CVS-B2c strain) or were mock infection treated. Subsequently, after a conventional data analysis for differentially expressed mRNA transcripts and a correlation-based analysis to identify time-dependent patterns of transcriptome-wide gene expression changes in response to RABV infection (Additional file 1: Figure S1), we used TopHat2 and Cufflinks [47] to perform a novel lncRNA species prediction and then conducted a similar differential expression analysis to identify lncRNAs which exhibited significant changes in their accumulation upon RABV infection. This identified 1434 differentially expressed lncRNAs (Fig. 1a). qPCR analysis successfully confirmed the significantly upregulated expression of ten of the most highly upregulated of these lncRNAs in response to RABV infection (Fig. 1b).

Pursuing the idea that lncRNAs accumulated in response to viral infection may somehow participate in cellular responses to RABV, we cloned six of the strongly upregulated lncRNAs and overexpressed them in N2a cells; these cells were then infected with pathogenic RABV at a low multiplicity of infection (MOI of 0.01). Excitingly, one of these—XLOC_007537, was predicted to be 1564 nt in length and to be transcribed from an intergenic locus on chromosome 11—was found to inhibit RABV infection in N2a cells (Fig. 1c and Additional file 1: Figure S2a). The 5′ and 3′ boundaries of this XLOC_007537 lncRNA were confirmed by 5′- and 3′-RACE experiments (Additional file 1: Figure S2b). The long 5′- and 3′-RACE sequences indicated there was no spanning exons in this novel lncRNA (Additional file 1: Figure S2b). This long intergenic non-coding RNA had no obvious annotation hits after examining its sequence using tools available with the NONCODEv5 [48], lncRNAdb 2.0 [49], or LNCipedia 5.0 [50] databases. Our PhyloCSF analysis [51] yielded a score of − 498.50 for this candidate lncRNA, indicating its non-coding characteristics (Additional file 1: Figure S2c). To further confirm its non-coding ability, we isolated the ribosome-RNA complex by size exclusion chromatography as previously reported [52], and then by using qPCR, we quantified the level of EDAL as well as a well-known lncRNA Malat1 and two coding RNAs Dennd1b and Crebrf. To be noted, Dennd1b and Crebrf displayed the comparable basal level with EDAL in N2a cells. The results showed that the level of ribosome-binding EDAL was far more less than lncRNA Malat1 and two coding RNAs, Dennd1b and Crebrf, indicating its non-coding characteristics (Additional file 1: Figure S2d). Since XLOC_007537 was found to cause EZH2 degradation in the following study, we named it as EZH2 degradation-associated lncRNA (EDAL). EDAL is partially conserved among rats, humans, rhesus, and chimps (Additional file 1: Figure S2e). By using a well-known cytoplasmic RNA control (18S ribosomal RNA, 18S) and nuclear RNA control (lncRNA Malat1), we found that EDAL was mainly localized in the cytoplasm of N2a cells (Fig. 1d). Consistently, RNA fluorescence in situ hybridization (FISH) revealed that EDAL occurs in both the cytoplasm and the nucleus, but the EDAL signal was stronger in the cytoplasm (Additional file 1: Figure S2f).

### Neuronal cell-specific accumulation of EDAL induced by viral infection

We next conducted experiments wherein N2a cells were infected with RABV at different doses for different periods, and EDAL levels were measured via qPCR over a time course of infection. We found that the extent of EDAL upregulation was dependent on the MOI used for viral infection (Fig. 1e), as well as on the infection duration (Fig. 1f): increased MOI and increased virus infection duration resulted in an increased extent of upregulation due to the accumulation of RABV. Besides RABV, we found several other neutropic viruses, including another negative-strand RNA viruses-VSV (Fig. 1g and Additional file 1: Figure S3a), a positive-strand RNA virus-SFV (Fig. 1h and Additional file 1: Figure S3b), and a DNA virus-HSV-1 (Fig. 1i and Additional file 1: Figure S3c), could also induce upregulation of EDAL. Additional experiments showed that only RABV viral genomic RNA could induce EDAL accumulation: viral proteins, double-stranded RNA (dsRNA), and interferons did not significantly induce EDAL (Fig. 1j and Additional file 1: Figure S3d-3g).

We then used qPCR to investigate both the basal level and the RABV-induced levels of EDAL in three mouse neuronal cell lines. These experiments revealed that the basal level of EDAL was much higher in N2a cells (neuron cell line) than that in glia cells, including BV2 (microglia cell line) and C8-D1A (astrocyte cell line) cells (Fig. 1k). After RABV infection, the level of EDAL in N2a was significantly upregulated, while no significant change in the EDAL level was detected in BV2 or C8-D1A cells (Fig. 1k). Furthermore, EDAL levels were much higher in brains and spinal cords than in the spleen, liver, or lung (Fig. 1l).

### EDAL inhibits viral replication

**Fig. 2:**
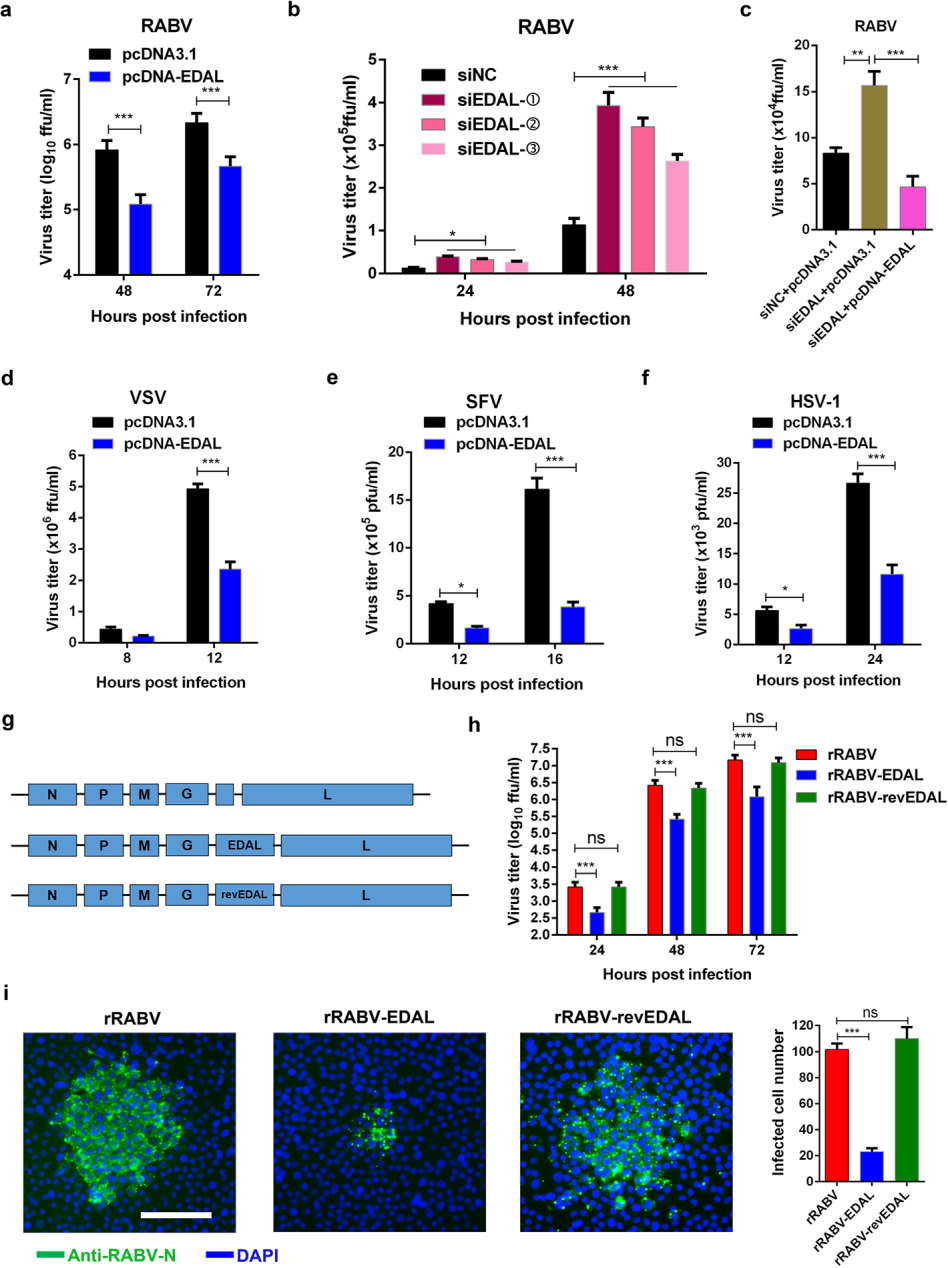
EDAL inhibits viral replication in neuronal cells. **a** N2a cells were transfected with pcDNA3.1 or pcDNA-EDAL, then at 12 h post transfection, the cells were infected with RABV at MOI 0.01 and virus titers were measured at indicated time points. **b** N2a cells were transfected with three different sets of EDAL-specific siRNAs (siEDAL-①, ②, ③). At 24 h post transfection, the cells were infected with RABV at MOI 0.01 and virus titers were measured at indicated time points. **c** N2a cells were transfected with siEDAL or siNC (negative control) for 8 h and then transfected with pcDNA3.1 or pcDNA-EDAL. At 12 h post transfection, the cells were infected with RABV at MOI 0.01 for 24 h and virus titers in the cell supernatant were measured. **d** N2a cells were transfected with pcDNA3.1 or pcDNA-EDAL, then at 12 h post transfection, the cells were infected with VSV at MOI 0.01 and virus titers were measured at indicated time points. **e** N2a cells were transfected with pcDNA3.1 or pcDNA-EDAL, then at 24 h post transfection, the cells were infected with SFV at MOI 0.01 and virus titers were measured at indicated time points. **f** N2a cells were transfected with pcDNA3.1 or pcDNA-EDAL, then at 12 h post transfection, the cells were infected with HSV-1 at MOI 0.01 and virus titers were measured at indicated time points. **g**, **h** EDAL and reverse EDAL (revEDAL) were inserted into the genome of a recombinant RABV (rRABV), named rRABV-EDAL and rRABV-revEDAL respectively (**g**), and their growth kinetics in N2a cells (MOI = 0.01) were compared (**h**). **i** N2a cells were infected with rRABV, rRABV-EDAL, or rRABV-revEDAL at MOI 0.005 for 48 h and the viral spread was compared by calculating the cell numbers within the fluorescence focus. Scale bar, 50 μm. Statistical analysis of grouped comparisons was carried out by Student’s *t* test(*P < 0.05;**P < 0.01; ***P < 0.001). Bar graph represents means ± SD, *n* = 3

We next transfected N2a cells with pcDNA3.1 plasmid expressing either EDAL (pcDNA-EDAL) or an EDAL-specific small interfering RNA (siEDAL) and then verified that EDAL was appropriately expressed or specifically silenced in N2a cells (Additional file 1: Figure S4a and 4b). We also confirmed that overexpression or silencing of EDAL did not affect cell viability (Additional file 1: Figure S4c-4d). Next, we transfected N2a cells with pcDNA-EDAL and then infected them with RABV at 12 h (h) post transfection. The viral titer in the supernatant of RABV-infected cells transfected with the pcDNA-EDAL vector was 8-fold lower than the titer of control cells transfected with the empty vector pcDNA3.1 at 48 h post infection (hpi). At 72 hpi, the same trend was apparent, but the difference was 4.5-fold (Fig. 2a).

The impact of EDAL silencing on virus titer was assessed using direct immunofluorescence assays with an antibody against the RABV N protein, which allowed calculation of the number of living RABV particles according to the number of immunofluorescent foci [53]. Excitingly, and consistent with a virus-replication-inhibiting function for EDAL in N2a cells, when the expression of EDAL was silenced by three different sets of siEDAL, the RABV titer increased by more than 2-fold compared to the siRNA control cells at 48 hpi. Among these three sets of siEDAL, siEDAL-① achieved the best silencing efficiency and was chosen to be used in the following study (Fig. 2b; Additional file 1: Figure S4b). The impact of siEDAL silencing was abolished by subsequent overexpression of EDAL, confirming that siEDAL was not off-target (Fig. 2c). Interestingly, a similar trend of reduced viral titers in cells transfected with pcDNA-EDAL was observed in VSV, SFV, and HSV-1-infected cells (Fig. 2d–f).

To further explore a role for EDAL in inhibiting viral replication, we next developed a series of recombinant viruses for later experiments with live mice. Specifically, we here used a recombinant RABV (rRABV) virus that was derived from the CVS-B2c strain, and used three different viral constructs: unaltered rRABV, rRABV harboring the EDAL sequence (rRABV-EDAL), and rRABV harboring the reverse complement sequence of EDAL (rRABV-revEDAL) (Fig. 2g). Virus growth kinetics experiments with N2a cells showed that the virus titer was significantly lower in the rRABV-EDAL-infected cells than both the rRABV-infected cells and the rRABV-revEDAL-infected cells (Fig. 2h).

We also analyzed the capacity of the recombinant viruses to spread between infected cells and neighboring cells, the infected N2a cells were covered by low melting agar to inhibit the virus release into the supernatant [53]. The rRABV-EDAL recombinant virus yielded much smaller fluorescent foci than rRABV and rRABV-revEDAL in the neighboring N2a cells (Fig. 2i, left) at 48 hpi, and the fluorescent foci we observed in the rRABV-EDAL-infected samples comprised significantly fewer cells than the fluorescent foci present in the rRABV or rRABV-revEDAL samples (Fig. 2i, right). In addition, pretreatment with anti-interferon receptor (IFNR) antibody in N2a cells did not abolish the antiviral function of EDAL, indicating that its antiviral activity is independent on IFN pathway (Additional file 1: Figure S4e).

### EDAL reduces RABV pathogenicity in vivo

**Fig. 3:**
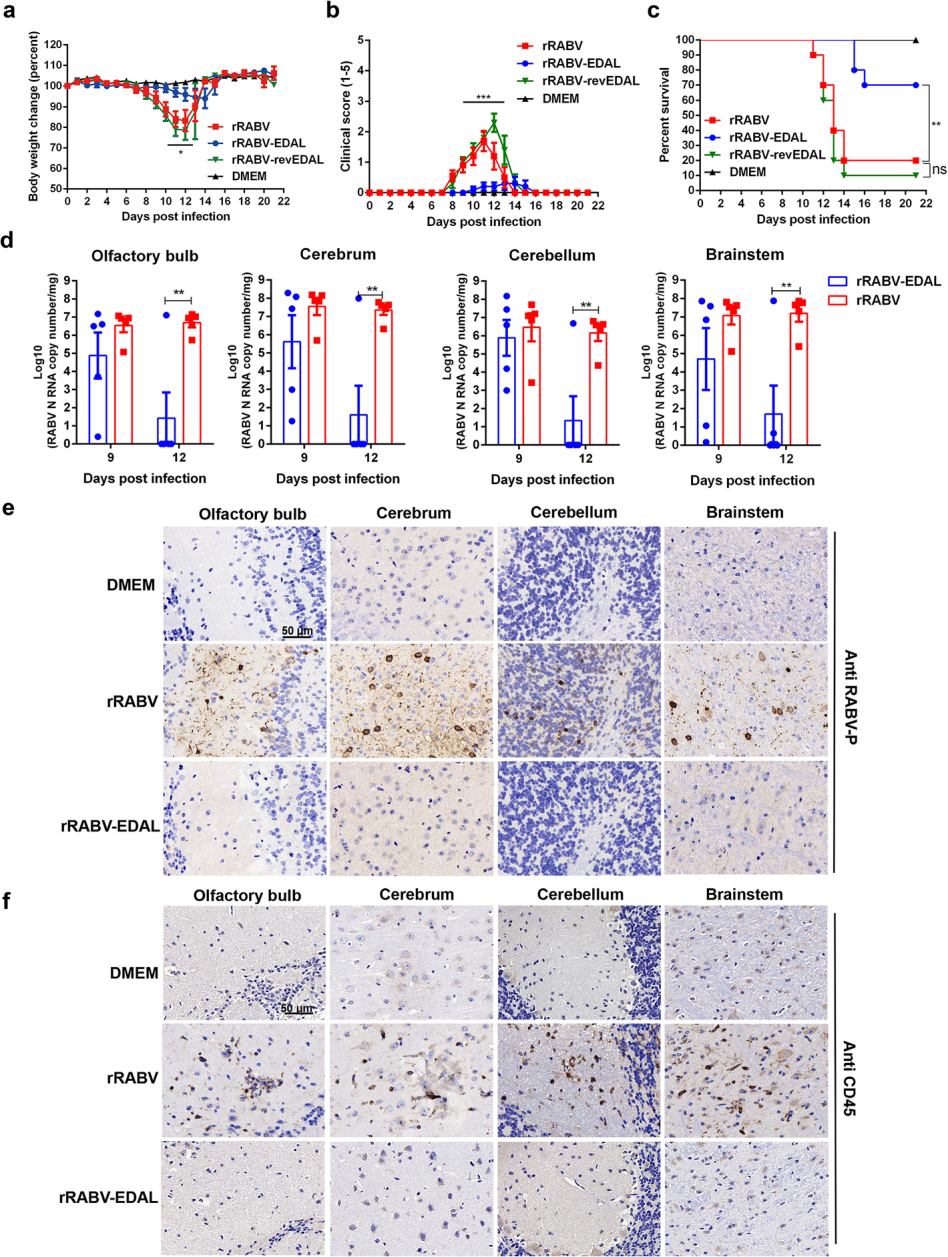
EDAL attenuates RABV pathogenicity in vivo. **a–c** Female C57BL/6 mice (8-week-old, *n* = 10) were infected intranasally with 100 FFU rRABV, rRABV-EDAL, or rRABV-reEDAL, or mock infected. Body weight change (**a**), clinical score (**b**), and survival ratio (**c**) were monitored daily for continuous 3 weeks (means ± SEM; **P < 0.01; body weight change and clinical score was analyzed by two-way ANOVA test; survival ratio was analyzed by log rank test). **d** At indicated time points, the brains from the infected mice were collected for analyzing the level of RABV N mRNA by qPCR (*n* = 5; means ± SEM; **P < 0.01 by Student’s two-way ANOVA test). **e**, **f** At 12 dpi, the brains were collected, resolved by paraffin sections, and analyzed by immunohistochemistry by staining with antibodies against RABV P (**e**) or CD45 (**f**). Scale bar, 50 μm

To investigate the role of EDAL in RABV infection in vivo, we compared the pathogenicity of rRABV, rRABV-EDAL, and rRABV-revEDAL in the C57BL/6 mouse model. Mice were infected intranasally (i.n.) with rRABV, rRABV-EDAL, or rRABV-revEDAL (100 FFU). The mice infected with rRABV and rRABV-revEDAL exhibited decreased body weights starting from 7 to 9 days post infection (dpi), and these decreases became significant between 9 and 14 dpi. In contrast, the body weight of mice infected with rRABV-EDAL only exhibited a slight decrease between 10 and 14 dpi (Fig. 3a). Moreover, the rabies symptoms (including weight loss, ruffled fur, body trembling, and paralysis) of the symptomatic rRABV- and rRABV-revEDAL-infected mice appeared at 7 dpi and became exacerbated until death at 14 dpi, whereas symptomatic mice infected with rRABV-EDAL had only mild symptoms which occurred from 9 to 15 dpi (Fig. 3b). Among all mice, 70% of the mice infected with rRABV-EDAL survived, compared with only 20% and 10% survival ratio for rRABV- and rRABV-revEDAL-infected mice, respectively (Fig. 3c).

To quantify the viral load in rRABV- and rRABV-EDAL-infected brains, the RABV *N* mRNA level in different encephalic regions was analyzed by qPCR after i.n. infection with 100 FFU of different viruses. At 12 dpi, we observed dramatically reduced RABV *N* mRNA levels in rRABV-EDAL-infected vs. rRABV-infected mice: specifically, these reductions were observed in the olfactory bulb, cerebrum, cerebellum, and brain stem regions (Fig. 3d). Further immunohistochemistry analysis of the RABV P protein (Fig. 3e) and CD45-positive cells (Fig. 3f) in various brain regions showed that, unlike rRABV-infected brains, almost no viral antigen or virus-induced inflammation could be observed in rRABV-EDAL-infected mouse brains at 12 dpi. Collectively, these results establish that EDAL can dramatically inhibit intranasal-inoculation-induced RABV infection in mice.

### EDAL decreases H3K27me3 levels by promoting lysosome-mediated EZH2 degradation

**Fig. 4:**
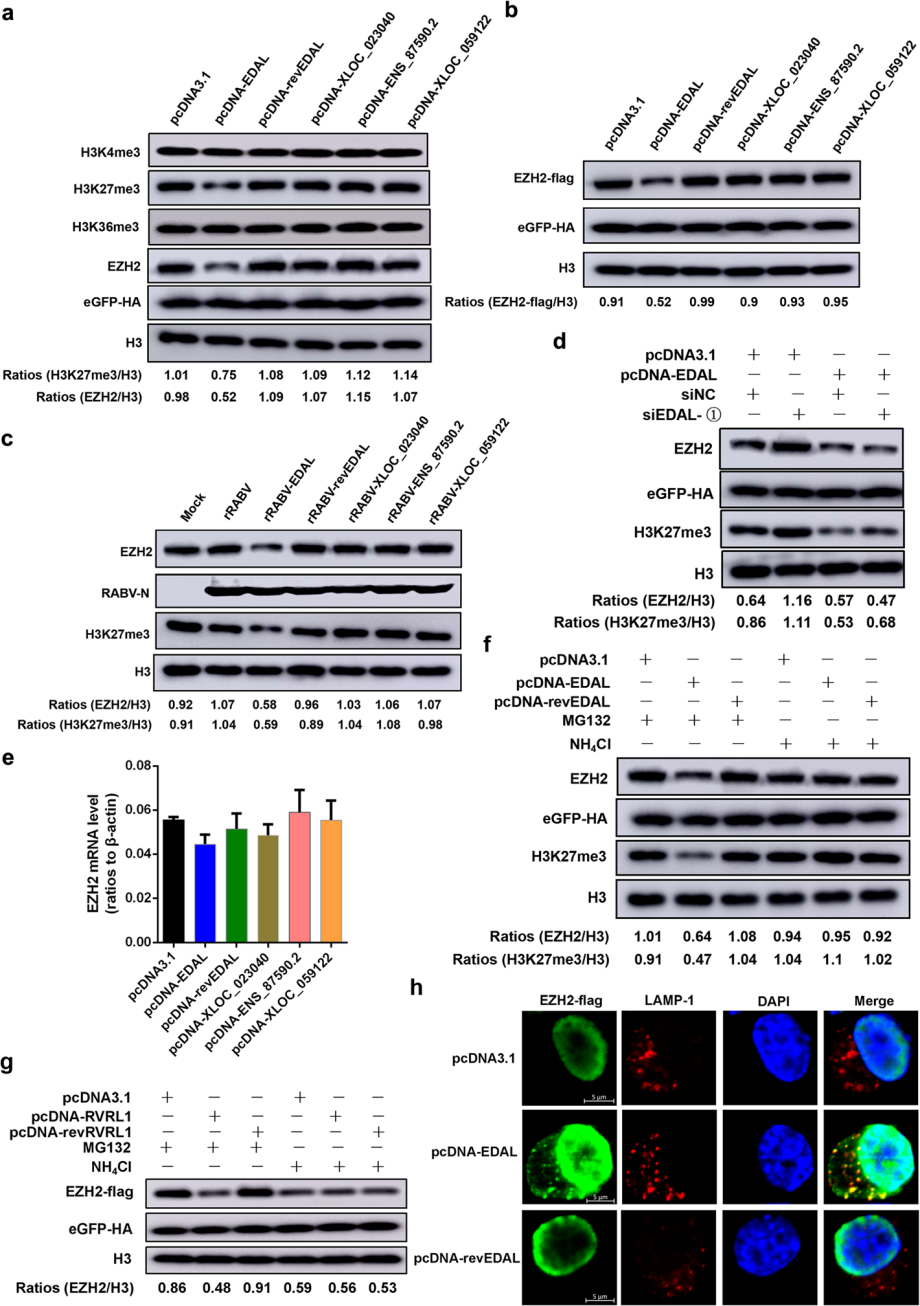
EDAL downregulates H3K27me3 level by causing the degradation of EZH2. **a** EDAL, reverse EDAL (revEDAL), XLOC_023040, ENSMUSG00000087590.2 (ENS_87590.2), or XLOC_059122 was overexpressed in N2a cells for 48 h and then EZH2, H3K4me3, H3K27me3, and K3K79me3 levels were measured by Western blotting. The plasmid pCAGGS-eGFP containing a HA tag was used as a transfection control. **b** N2a cells were transfected with pcDNA3.1, pcDNA-EDAL, pcDNA-revEDAL, pcNDA-XLOC_023040, pcDNA-ENS_87590.2, or pcDNA-XLOC_059122, and pCAGGS-EZH2-FLAG and pCAGGS-eGFP-HA (transfection control). EZH2-FLAG levels were measured by Western blotting and normalized to H3. **c** N2a cells were infected with rRABV, rRABV-EDAL, rRABV-revEDAL, rRABV-XLOC_023040, rRABV-ENS_87590.2, or rRABV-XLOC_059122 at MOI 3. At 36 hpi, the EZH2 and H3K27me3 levels were resolved by Western blotting and normalized to H3. **d** N2a cells were transfected with siEDAL or siNC (negative control) for 8 h and then transfected with pcDNA3.1 or pcDNA-EDAL. Then EZH2 and H3K27me3 levels were resolved by Western blotting and normalized to H3. **e** N2a cells were transfected with pcDNA3.1, pcDNA-EDAL, pcDNA-revEDAL, pcNDA-XLOC_023040, pcDNA-ENS_87590.2, or pcDNA-XLOC_059122. The mRNA levels of EZH2 were analyzed by qPCR (*n* = 3). **f** pcDNA3.1, pcDNA-EDAL, or pcDNA-revEDAL was transfected into N2a cells. The specific inhibitors for proteasome and lysosome, MG132 (10 μM) and NH_4_Cl (5 mM), were applied. Then EZH2 and H3K27me3 levels were analyzed by Western blotting and normalized to H3. **g** pcDNA3.1, pcDNA-EDAL, or pcDNA-revEDAL was transfected together with pCAGGS-EZH2-flag into N2a cells. The specific inhibitors for proteasome and lysosome, MG132 (10 μM) and NH_4_Cl (5 mM), were applied. Then EZH2-flag level was analyzed by Western blotting and normalized to H3. **h** pcDNA3.1, pcDNA-EDAL, or pcDNA-revEDAL was transfected together with pCAGGS-EZH2-flag into N2a cells. At 36 h post transfection, EZH2-flag and LAMP-1 were analyzed by immunofluorescence. Scale bar, 5 μm. Western blot data are representative of at least two independent experiments

Having demonstrated that RABV infection induces the accumulation of EDAL and established that EDAL can restrict RABV replication in vitro and in vivo, we were interested in potential mechanism(s) through which EDAL may exert its antiviral effects. We have for some time been interested in the potential contributions of epigenetic regulation on host responses to neurotropic viruses, and we noted that the N2a cells transfected with the pcDNA-EDAL plasmid had significantly decreased levels of histone methylation. Specifically, immunoblotting experiments with an antibody against the H3K27me3 tri-methylation mark revealed that cells with the empty control plasmid had a signal for this histone methylation of the N-terminal tail of the core histone H3 that was 1.35 times as strong as the signal for cells transfected with the pcDNA-EDAL plasmid. In contrast, no significant change for the H3K4me3 or H3K36me3 mark could be observed post EDAL expression (Fig. 4a).

To confirm an impact specifically from EDAL on the observed reduction in the H3K27me3 tri-methylation level, we evaluated three other lncRNAs from our dataset which were induced by RABV, namely XLOC_023040, ENSMUSG00000087590.2 (ENS_87590.2) and XLOC_059122 mentioned in Fig. 1c. Notably, the expression of these lncRNAs did not change the H3K27me3 tri-methylation level (Fig. 4a), strongly supporting the specificity of EDAL in exerting this inhibitory effect. These results led us to speculate that EDAL may interfere with viral replication via alteration of histone methylation.

It is now understood that PRC2 mediates the H3K27me3 tri-methylation process [54], so we performed additional immunoblotting with an antibody against EZH2—the enzymatic subunit of PRC2 responsible for its methyl-transferase activity. As with the signal for H3K27me3 tri-methylation, we observed weaker signals for EZH2 in cells with the plasmid for pcDNA-EDAL compared to controls (Fig. 4a, b). We next used the recombinant viruses that we used for mice infection (Fig. 3) to repeat the above experiments, and the same decreasing trend was observed in N2a cells infected with the rRABV-EDAL virus (Fig. 4c). Moreover, no such decreases in the H3K27me3 tri-methylation signal or the EZH2 protein level were observed upon expression of revEDAL or the three aforementioned lncRNAs (Fig. 4c), again highlighting an apparently specific contribution of EDAL to the reduced levels of H3K27me3 and its catalyst EZH2.

To further determine the impact of EDAL on the H3K27me3 tri-methylation signal and/or the EZH2 protein level, N2a cells were transfected with siEDAL. Consistently, silencing of EDAL enhanced the levels of both EZH2 and H3K27me3 in N2a cells (Fig. 4d), and overexpression of EDAL counteracted the elevated EZH2 level induced by siEDAL (Fig. 4d). Importantly, we also found that the EZH2 protein level, but not the *EZH2* mRNA level, was reduced by EDAL—and noted that expression of revEDAL or other three control lncNRAs did not affect the protein or the mRNA level for EZH2 (Fig. 4e)—results clearly suggesting that the impact of EDAL on EZH2 accumulation occurs at the protein level.

We therefore suspected that an EDAL–EZH2 interaction might somehow promote the degradation of EZH2, thereby reducing the overall cellular capacity for its methyltransferase activity, potentially explaining the observed reduction in H3K27me3 tri-methylation. To test this hypothesis, we treated cells with compounds that inhibit the protein degradation functions of proteasomes (MG132) or lysosomes (NH_4_Cl), and then assayed the EZH2 protein accumulation and the H3K27me3 tri-methylation level upon EDAL expression. These experiments showed that NH_4_Cl but not MG132 treatment restored the EZH2 protein and H3K27me3 tri-methylation levels, results supporting that EDAL somehow causes the endogenous EZH2 degradation via the lysosomal degradation pathway (Fig. 4f). Then we further confirmed these observations by overexpressing EZH2 and EDAL in N2a cells treated with NH_4_Cl or MG132. The results indicated that NH_4_Cl but not MG132 treatment restored the degraded EZH2 protein level by EDAL (Fig. 4g). To further confirm that EZH2 is degraded via lysosomal pathway, we transfected pcDNA3.1, pcDNA-EDAL, or pcDNA-revEDAL together with pCAGGS-EZH2-flag plasmids into N2a cells and then performed indirect immunofluorescence to localize EZH2 and lysosomes by using antibodies against flag-tag and the lysosomal marker LAMP-1. As expected, EZH2 were located in nucleus when transfected with pcDNA3.1 or pcDNA-revEDAL, but partially relocated to lysosomes post EDAL expression (Fig. 4h). These results suggest that EDAL expression causes EZH2 degradation via the lysosomal degradation pathway.

### A 56 nt 5′ segment is responsible for EDAL’s antiviral activity

**Fig. 5:**
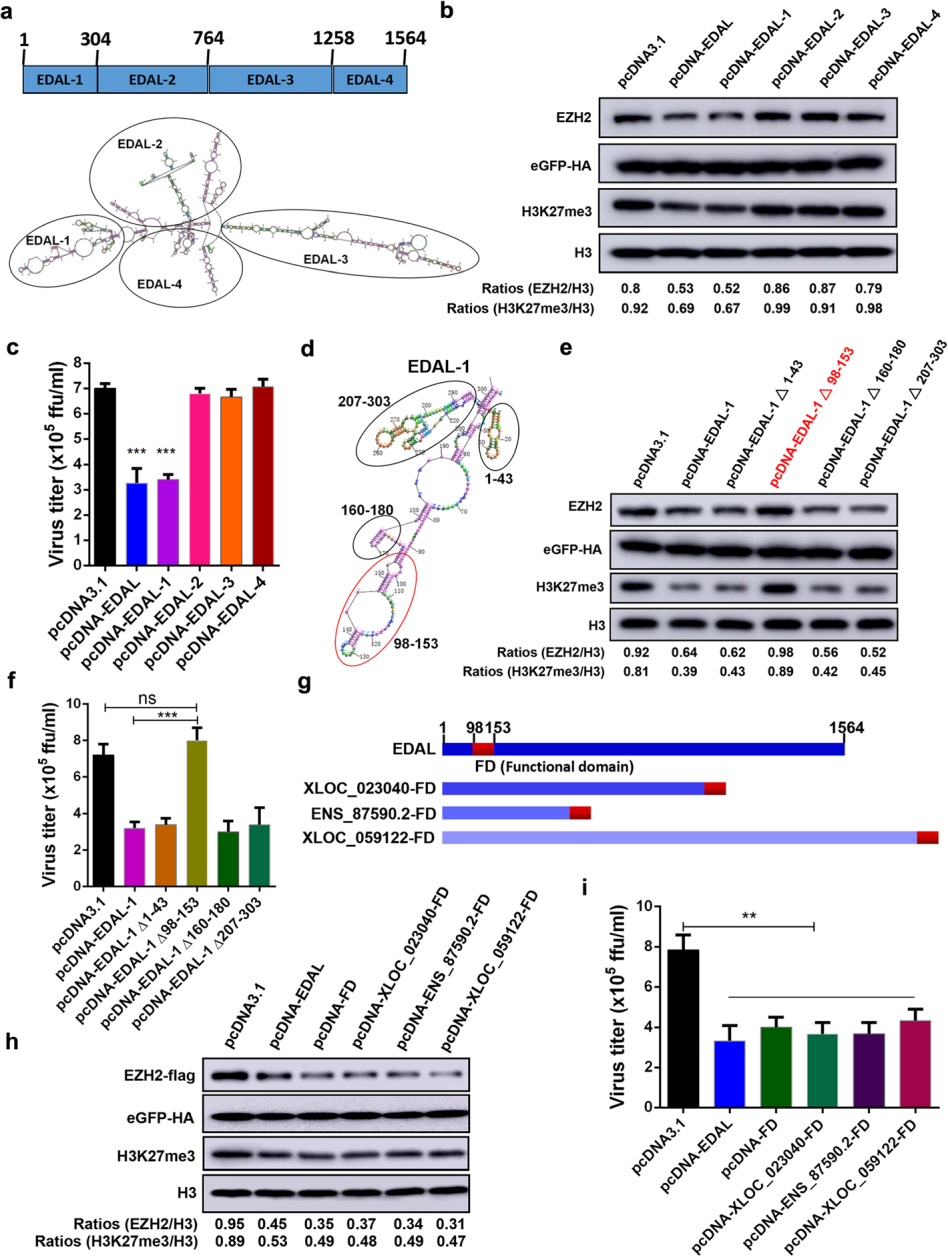
The 56-nt portion of EDAL in 5′ end carries the antiviral function. **a** EDAL secondary structure was predicted by RNAstructure Version 5.8 software (https://rnastructure.software.informer.com/). EDAL was divided into four sections based on sub-structures: EDAL-1 (1–304 nt), EDAL-2 (305–764 nt), EDAL-3 (765–1258 nt), and EDAL-4 (1259–1564 nt). **b** The full-length EDAL and its truncations were separately transfected into N2a cells for 48 h. The EZH2 and H3K27me3 levels were resolved by Western blotting and the ratio normalized to H3 was calculated. **c** The full-length EDAL and its truncations were expressed in N2a cells for 12 h and then the cells were infected with RABV at MOI 0.01. At 48 hpi, the virus titers in the cell supernatant were measured. **d**, **e** Four sections within EDAL-1 were selected based on the secondary structures (**d**). The four truncations EDAL-1 deleting 1–43 nt (EDAL-1 ∆1–43), 98–153 nt (EDAL-1 ∆98–153), 160–180 nt (EDAL-1 ∆160–180), and 207–303 nt (EDAL-1 ∆207–303) were cloned into pcDNA3.1, respectively. The different truncations as well as full-length EDAL-1 were overexpressed in N2a cells for 48 h. Then EZH2 and H3K27me3 levels were resolved by Western blotting and normalized to H3 (**e**). **f** N2a cells were transfected with pcDNA3.1, pcDNA-EDAL-1, or different truncations of EDAL-1 for 12 h. Then the cells were infected with RABV at MOI 0.01 and the virus titers in supernatant were measured at 48 hpi. **g**, **h** The functional domain (FD) of the 56-nt portion of EDAL was cloned into pcDNA3.1 or fused with 3′ end of the other three control lncRNAs (**g**). Then these lncRNAs were transfected together with pCAGGS-EZH2-flag into N2a cells for 48 h. EZH2 and H3K27me3 levels were analyzed by Western blotting and normalized to H3 (**h**). **i** N2a cells were transfected with pcDNA3.1, pcDNA-EDAL, or different recombinant lncRNAs for 12 h. Then the cells were infected with RABV at MOI 0.01 and the virus titers in supernatant were measured at 48 hpi. Statistical analysis of grouped comparisons was carried out by Student’s *t* test(**P < 0.01; ***P < 0.001). Bar graph represents means ± SD, *n* = 3. Western blot data are representative of at least two independent experiments

Although not necessarily conserved, secondary structures are thus far good candidates for identification of functional elements of lncRNAs [18, 55–57]. Seeking to identify secondary structures of EDAL that affect its specific interaction with EZH2, predictions using the RNAstructure 5.3 program indicated that EDAL could be divided into four major sub-structures, each containing a number of base-paired structures and hairpin structures (Fig. 5a). We cloned the segments corresponding to the four sub-structures (EDAL-1, EDAL-2, etc.) into pcDNA3.1, and then each of the four segments was individually expressed in N2a cells, followed by immunoblotting-based evaluation of the EZH2 protein and H3K27me3 tri-methylation levels. Interestingly, the first truncated segment (EDAL-1) located at the 5′ end of EDAL, but none of the other three segments, significantly reduced both the EZH2 and H3K27me3 levels (Fig. 5b). Consistent with a specific impact from this EDAL sub-structure, only EDAL-1 restricted RABV replication in N2a cells (Fig. 5c).

To pinpoint the specific fragment capable of exerting the antiviral function, EDAL-1 was assessed as four separate truncation segments (EDAL-1 ∆1–43, EDAL-1 ∆98–153, EDAL-1 ∆160–180, and EDAL-1 ∆207–303) (prepared as depicted in Fig. 5d). Each of the EDAL-1 variants were assessed in N2a cells: only EDAL-1 ∆98-153 failed to decrease the EZH2 and H3K27me3 levels and failed to inhibit rRABV replication (Fig. 5e, f).

To confirm that EDAL 98–153 nt can inhibit RABV infection, this 56 nt segment was expressed by itself and as a fusion with the 3′ end of the three aforementioned lncRNAs (i.e., from our experiments to successfully demonstrate the specificity of EDAL’s antiviral effects) (Fig. 5g). As expected, the fragment alone and the three fusion lncRNAs reduced the EZH2 and H3K27me3 levels (Fig. 5h) and also reduced RABV replication (Fig. 5i). These results establish that the 56-nt segment at the 98–153 position of the 5′ end of EDAL is essential for the EZH2-mediated antiviral effects we observed in neuronal cells.

In order to probe whether the 304-nt EDAL-1 and the 56-nt functional RNA unit formed the predicted secondary structures, we in vitro transcribed these two RNA molecules by T7 RNA polymerase and probed their secondary structures by limited enzymatic digestion using micrococcal ribonuclease, an endo-exonuclease that preferentially digests single-stranded nucleic acids at the 5′ side of A or T. The digested RNA fragments were ligated with 5′ and 3′ adaptors followed by cDNA library preparation and Illumina sequencing. After mapping on the corresponding RNA molecule sequence, we recovered both the 5′-end and 3′-end nucleotide signals representing the cleavage site from mapped reads. Plotting the cleavage signals showed that ribonuclease probing results were primarily in good accordance with the predicted structures of both molecules (Additional file 1; Figure S5 and Additional file 2: Table S1). To be noted, the presence of 5 mM MgCl_2_ enhanced the stem-loop structures in general, exposing the loop regions to be more accessible by micrococcal ribonuclease.

### EDAL reduces EZH2 stability by impeding an *O*-GlcNAcylation PTM at the T309 site

Previous studies have revealed that phosphorylation and *O*-GlcNAcylation can influence the stability of EZH2 [40–42]. At least two phosphorylation sites among human EZH2, T345 and T487, were shown to affect its stability [40]. However, we found that EDAL could still cause the degradation of murine EZH2 when the corresponding phosphorylation sites were mutated to T341A and T485A, (Additional file 1: Figure S6a), indicating that EDAL does not apparently impair the phosphorylation of EZH2.

**Fig. 6:**
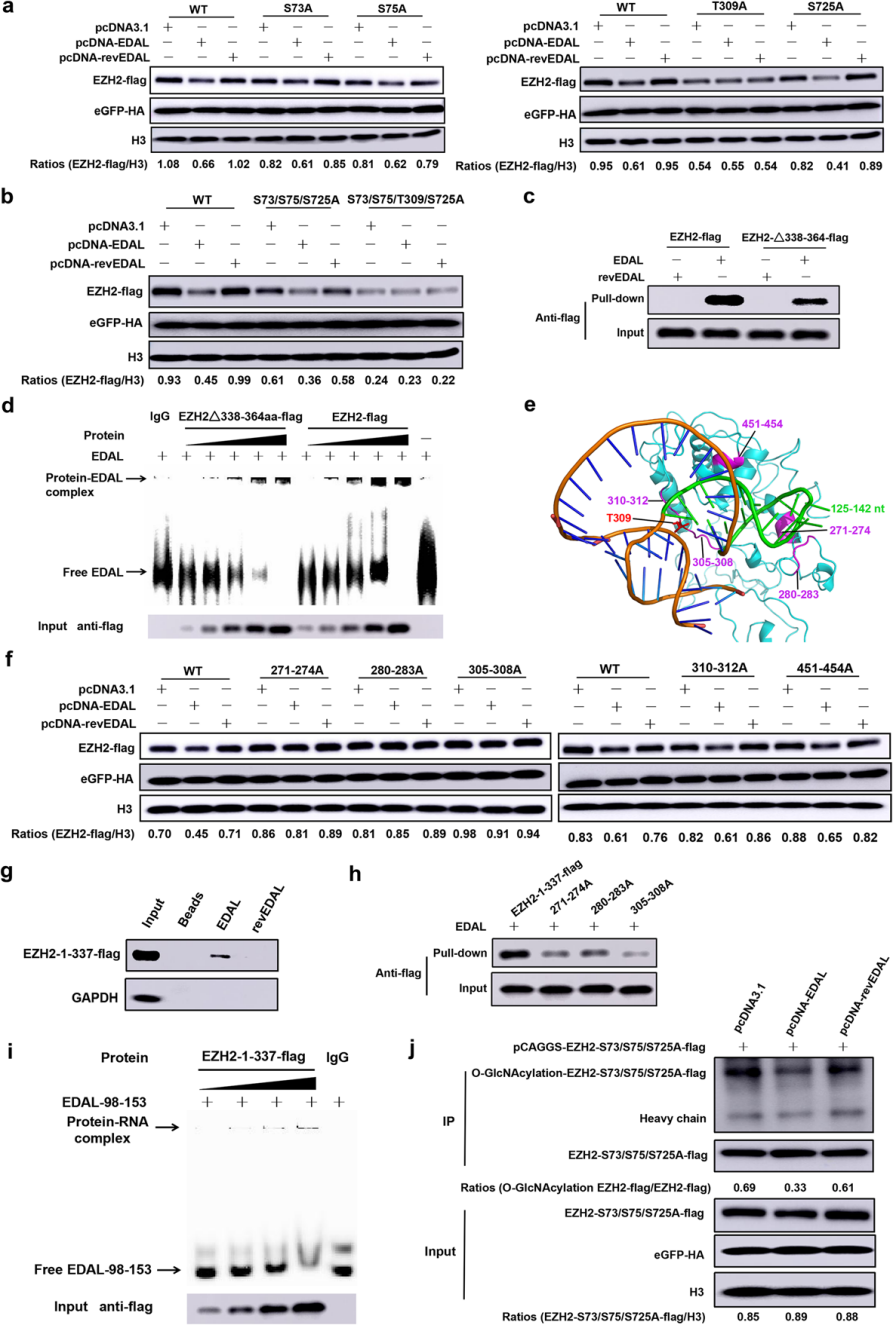
EDAL promotes EZH2 degradation via impeding the *O*-GlcNAcylation at T309 site. **a** The potential *O*-GlcNAcylation sites of murine EZH2 was individually mutated and expressed together with EDAL or revEDAL in N2a cells for 48 h. Then EZH2 level was analyzed by Western blotting and normalized to H3. **b** The potential *O*-GlcNAcylation sites of murine EZH2 were mutated and co-expressed together with EDAL or revEDAL in N2a cells for 48 h. Then EZH2 level was analyzed by Western blotting and normalized to H3. **c** N2a cells were transfected with EZH2-flag or EZH2∆338–364-flag for 48 h. Then RNA pull-down was performed to determine the interaction between EZH2 and EDAL (or revEDAL), respectively. **d** N2a cells were transfected with EZH2-flag or EZH2∆338–364-flag for 48 h. The targeted proteins were pulled down with flag-tag monoclonal antibody, and then EMSA was performed to determine the interaction between EZH2 and EDAL. **e** Murine EZH2 3D structure was predicted with SWISS-MODEL (https://swissmodel.expasy.org/interactive) based on human EZH2 3D structure (PDB code: 5HYN). EDAL-FD 3D structure model was predicted with RNAComposer (http://rnacomposer.ibch.poznan.pl/). The interaction between EDAL functional domain (98–153 nt) and EZH2 was predicted by 3dRPC. The predicted interactional residues among EZH2 were marked with magenta color and among EDAL with green color. **f** The predicted interaction residues of EZH2 were mutated and cloned into pCAGGS vector, and then co-transfected with pcDNA3.1, pcDNA-EDAL, or pcDNA-revEDAL in N2a cells for 48 h. Then EZH2 level was analyzed by Western blotting and normalized to H3. The plasmid pCAGGS-eGFP containing a HA tag was used as a transfection control. **g**, **h** The truncated EZH2 1–377 and other three mutants with alanine substitution at the 271–274, 280–283, or 305–308 aa based on EZH2 1–377 were cloned into pCAGGS vector, and then transfected into N2a cells for 48 h. RNA pull-down was performed to determine the interaction between EZH2 1–377 (**g**) or its mutants (**h**) and EDAL. **i** N2a cells were transfected with EZH2-1-337-flag for 48 h. EZH2-1-337-flag was pulled-down with flag-tag monoclonal antibody, and then EMSA was performed to determine the interaction between EZH2-1-337-flag and EDAL-98-153. **j** The plasmid expressing EZH2-S73/S75/S725A-flag was co-transfected with pcDNA3.1, pcDNA-EDAL, or pcDNA-revEDAL in N2a cells and treated with NH_4_Cl (5 mM) for 48 h. Then the *O*-GlcNAcylation level of EZH2-S73/S75/S725-flag was analyzed by Western blotting. Western blot data are representative of at least two independent experiments

There are five known *O*-GlcNAcylation sites (S73, S76, S84, T313, and S729) in human EZH2 that can regulate EZH2 stability and enzymatic activity [41, 42]. We found that endogenous or over-expressed murine EZH2 could be detected by an antibody specially against *O*-Linked N-Acetylglucosamine, RL2, which indicated that murine EZH2 was modified by *O*-GlcNAcylation (Additional file 1: Figure S6b). Based on the sequence alignment between human and murine EZH2, we found that S73, S75, T309, and S725 are potential *O*-GlcNAcylation sites of murine EZH2 (Additional file 1: Figure S6c). We mutated each of the potential *O*-GlcNAcylation sites of murine EZH2 and then co-transfected these mutant variants together with pcDNA3.1, pcDNA-EDAL, or pcDNA-revEDAL in N2a cells. We found only T309A mutation lost the EDAL-promoted EZH2 degradation (Fig. 6a), while there was no significant difference in the extent of degradation among the wild type (WT), S73A, S75A, or S725A variants of EZH2 (Fig. 6a). We observed the same trends for EZH2 variants bearing multiple mutations: a S73/S75/S725 triple-alanine-mutant did not affect EDAL-promoted EZH2 degradation, whereas EDAL lost its impact on the degradation of a tetra-alanine EZH2 variant with mutation of position 309 (Fig. 6b). These results together indicated that EDAL mediated EZH2 degradation via specifically blocking T309 *O*-GlcNAcylation site.

We speculate that EDAL may bind with EZH2 and then block its T309 *O*-GlcNAcylation site. Thus, we performed RNA pull-down and RNA electrophoretic mobility shift assay (EMSA) to investigate if EDAL could bind with EZH2. Previous studies have demonstrated that there is a well-known RNA binding domain in 343–368 aa of human EZH2, which can bind many lncRNAs with limited specificity [58], and this corresponding region in murine EZH2 was 338–364 aa. Thus, the murine EZH2 deleting 338–364 aa was constructed and included as a control. As expected, RNA pull-down (Fig. 6c) and EMSA (Fig. 6d) results showed that EDAL could bind both EZH2 and EZH2 deleting 338–364 aa, suggesting that besides 338–364 aa there are other EDAL binding sites in EZH2.

In order to further pursue the EDAL-EZH2 interactions which may contribute to the EDAL-specific blocking of the EZH2 T309 *O*-GlcNAcylation, we decided to predict the interaction sites between the 56-nt antiviral EDAL substructure and EZH2. RNA tertiary structure prediction revealed a tertiary structure for the 56-nt antiviral RNA segment: the helix-loop tertiary structure folded by the 18-nt terminal hairpin corresponding to 125–142 aa of EDAL was packed on the second helix folded by the stem base-paired structure, and most of the two structural components were free for contacting other partners (Fig. 6e). We then conducted for molecular docking using the 3dRPC program taking the advantage of recently published tertiary structures for EZH2 [32, 33, 59, 60]. Among the top scored structures, one shows that the 18-nt terminal helix-loop tertiary structure was intimately interacted with EZH2 residues at positions 271–274, 280–283, 305–308, 310–312, and 451–454 aa (Fig. 6e). To validate these predicted interactions, we mutated all these EDAL interacting residues in EZH2 to alanine (A). We co-transfected N2a cells with plasmids expressing wild type EZH2 and EZH2 mutant variants together with the pcDNA3.1, pcDNA-EDAL, or pcDNA-revEDAL plasmids. The results revealed a striking difference: in the presence of EDAL, there was no obvious reduction in the levels of the EZH2 variants bearing alanine substitution mutations at the 271–274, 280–283, or 305–308 aa positions, whereas there was obvious degradation of WT EZH2 and the other variants (Fig. 6f). Thus, the cellular stability of EZH2 is directly affected by an interaction between EDAL and the EZH2 residues at positions 271–274, 280–283, and 305–308 aa. In order to verify these binding sites between murine EZH2 and EDAL, we truncated murine EZH2 into 1–337 aa (EZH2 1–377) and constructed another three mutants with alanine substitution at the 271–274, 280–283, or 305–308 aa based on EZH2 1–377. RNA pull-down assay showed that EDAL could bind the EZH2 N-terminal region (1–337 aa) (Fig. 6g), and 271–274, 280–283, and 305–308 aa were the critical binding sites (Fig. 6h). EMSA results demonstrated that the 56-nt segment at the 98–153 position of the 5′ end of EDAL was associated with EZH2 1–377 (Fig. 6i).

The molecular docking and validation experiments supported a model that EDAL can specifically bind to EZH2 at the T309 *O*-GlcNAcylation site. We therefore speculated that EDAL binding might impair the *O*-GlcNAcylation at T309 site, potentially preventing an EZH2-stability-promoting effect associated with this PTM. Pursuing this, we evaluated the effect of EDAL expression on the *O*-GlcNAcylation level of EZH2 at the T309 site. To exclude the impact of other *O*-GlcNAcylation sites on the detected level of EZH2 *O*-GlcNAcylation, pCAGGS-EZH2-S73/S75/S725A-flag plasmid was transfected together with pcDNA3.1, pcDNA-EDAL, or pcDNA-revEDAL into N2a cells, and then the *O*-GlcNAcylation level on the EZH2-S73/S75/S725A-flag fusion protein was measured post treatment with NH_4_Cl. Interestingly, we found that expression of EDAL dramatically reduced the *O*-GlcNAcylation level of EZH2 (Fig. 6j).

To further verify that EZH2 T309 was modified with *O*-GlcNAcylation. We pre-treated the cells with OSMI-1, a GlcNAc-transferase (OGT) inhibitor, and then we transfected cells with EZH2-flag, EZH2-S73/S75/S725A-flag, or EZH2-S73/S75/T309/S725A-flag plasmids. Both WT EZH2 and EZH2-S73/S75/S725A levels were obviously reduced, while the EZH2-S73/S75/T309/S725A-flag level remained unchanged (Additional file 1: Figure S6d). Reciprocally, we transfected OGT together with EZH2-flag, EZH2-S73/S75/S725A-flag, or EZH2-S73/S75/T309/S725A-flag plasmids into N2a cells and found that both WT EZH2 and EZH2-S73/S75/S725A expression levels were upregulated, while EZH2-S73/S75/T309/S725A-flag expression level almost kept unchanged, further indicating *O*-GlcNAcylation of EZH2 T309 site impacts its stability (Additional file 1: Figure S6e). These results support that EDAL specifically contacts T309, shielding T309 from *O*-GlcNAcylation.

### The EZH2 inhibitor gsk126 protects neuronal cells from viral infection

If EDAL’s antiviral effects are indeed mediated by its reduced EZH2 methyltransferase activity, then we could anticipate that chemical inhibition of EZH2 should cause antiviral effects. Gsk126 is a specific inhibitor of EZH2 methyltransferase activity [61], and we evaluated the effects of gsk126 on RABV and VSV replication in N2a cells. After testing toxicity (Additional file 1: Figure S7a) and identifying a suitable working concentration of gsk126 (Additional file 1: Figure S7b), we pretreated N2a cells with 4 μmol (μM) gsk126 and then infected them with rRABV or VSV. The replication of both rRABV (Additional file 1: Figure S7c) and VSV (Additional file 1: Figure S7d) was significantly decreased by treatment with gsk126, results which reinforce a specific role for EZH2’s methyltransferase activity on the antiviral effects we observed in N2a cells and which demonstrate proof-of-concept for a therapeutic strategy against a neurotropic virus.

### EDAL restricts viral replication by upregulation of an antiviral peptide PCP4L1

**Fig. 7:**
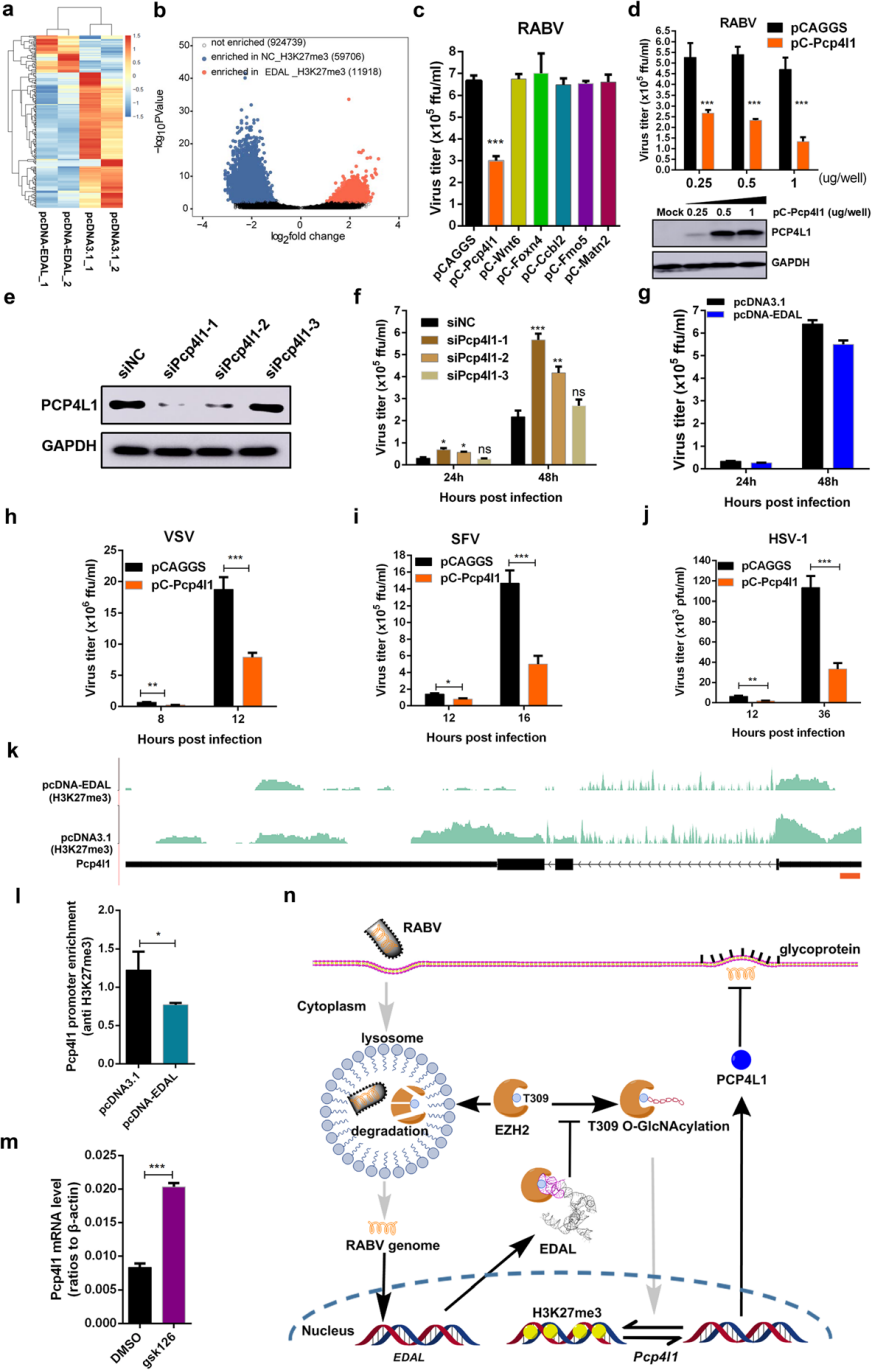
EDAL restricts viral replication by upregulation of *Pcp4l1*. **a** N2a cells were transfected with pcDNA3.1 or pcDNA-EDAL for 12 h and then infected with RABV at MOI 1 for 48 h. Total RNA was isolated and subjected to RNA-seq analysis (*n* = 2; 2 fold change (FC) and 0.01 *p* value). **b** N2a cells were transfected with pcDNA3.1 or pcDNA-EDAL for 48 h and then ChIP-seq analysis was performed. Volcano plot showed the peaks enriched in negative control (NC) cells and EDAL overexpression cells. The *X* axis was the log2 ratio of EDAL versus NC signals for each peak, and the *Y* axis was the significance of the differences (−log10 (*p* values)). **c** Six upregulated and loss of H3K27me3 mark genes were cloned into the mammalian expression vector pCAGGS and overexpressed in N2a cells. At 12 h post transfection, the cells were infected with RABV for 48 h at MOI 0.01, and virus titers in the supernatant were measured. **d** N2a cells were transfected with pCAGGS-*Pcp4l1* (pC-*Pcp4l1*) at indicated dose for 12 h, and then infected with RABV at MOI 0.01. At 48 hpi, the virus load in the cell supernatant was measured. PCP4L1 expression level was analyzed by Western blotting. **e** N2a cells were transfected with siNC or three different sets of siPcp4l1 for 72 h and then PCP4L1 protein level was analyzed by Western blotting. **f** N2a cells were transfected with siNC or three different sets of siPcp4l1 for 24 h and then infected with RABV at MOI 0.01. At indicated hpi, virus load in cell culture was measured. **g** N2a cells were transfected with siNC or siPcp4l1–1 for 24 h and then transfected with pcDNA3.1 or pcDNA-EDAL for 12 h. Cells were then infected with RABV at MOI 0.01 and virus load in cell culture was measured at indicated hpi. **h** N2a cells were transfected with pCAGGS-*Pcp4l1* (pC-*Pcp4l1*) for 12 h, and then infected with VSV at MOI 0.01. At indicated hpi, the virus load in the cell supernatant was measured. **i** N2a cells were transfected with pC-*Pcp4l1* for 24 h, and then infected with SFV at MOI 0.01. At indicated hpi, the virus load in the cell supernatant was measured. **j** N2a cells were transfected with pC-*Pcp4l1* for 24 h, and then infected with HSV-1 at MOI 0.01. At indicated hpi, the virus load in the cell supernatant was measured. **k** Sequencing profile of *Pcp4l1* for ChIP-seq. The two tracks show H3K27me3 signals for pcDNA3.1 and pcDNA-EDAL samples after removing input background. The brown rectangle indicates the predicted promoter region of *Pcp4l1*. **l** N2a cells were transfected with pcDNA-EDAL or pcDNA3.1 for 48 h, and then ChIP-qPCR were performed with H3K27me3 antibody in the promoter region of *Pcp4l1*. **m** N2a cells were treated with 4 μM gsk126 or DMSO (mock) for 48 h and *Pcp4l1* mRNA level was analyzed by qPCR. **n** Proposed model for EDAL-induced EZH2 lysosomal degradation, and the potential subsequent impact on EZH2-mediated epigenetic silencing of *Pcp4l1.* Statistical analysis of grouped comparisons was carried out by Student’s *t* test (**P < 0.01; ***P < 0.001). Bar graph represents means ± SD, *n* = 3. Western blot data are representative of at least two independent experiments

Next we attempt to identify the genes which might be upregulated by EDAL via decreasing H3K27me3 levels. N2a cells were transfected with pcDNA-EDAL or pcDNA3.1, and then infected with RABV at MOI 1. At 48 hpi, the poly(A)-RNA was isolated for deep sequencing. A cut-off of 0.05 FDR resulted in a total of 75 upregulated genes (Fig. 7a). We next attempted to identify the targeted genes regulated by EDAL. We turned our attention to the altered H3K27me3 modification as an additional selection criterion for EDAL to induce EZH2 degradation and reduce the H3K27me3 level. Chromatin immunoprecipitation sequencing (ChIP-seq) was performed by using anti-H3K27me3 antibody to profile the distribution of H3K27me3 marks on the genome of N2a cells upon transfection with pcDNA-EDAL or control plasmids, and then the data were summarized in Additional file 3: Table S2. Analysis of H3K27me3 peaks indicative of the epigenetic silencing positions revealed many fewer peaks—11,918 vs. 59,706—in EDAL overexpressed samples compared with the samples transfected with empty control plasmids, consistent with the EDAL-reduced cellular level of H3K27me3. In total, 2026 genes lost H3K27me3 mark and only 167 genes gained after EDAL overexpression (Fig. 7b). Most EDAL-upregulated genes naturally did not contain H3K27me3 mark, consistent with a recent report that many H3K27me3 marks in adult mice is not related to transcriptional regulation [62].

The EDAL-response genes with upregulated transcription and the loss of H3K27me3 mark should represent candidate genes whose expression was subjected to the EDAL-EZH2 regulation, which we considered for further investigation. Based on the results of RNA-seq and ChIP-seq, 6 most upregulated genes, losing H3K27me3 mark simultaneously, were selected and evaluated whether they could restrict RABV replication. These genes were overexpressed by transient transfection in N2a cells and then RABV was infected at 12 h later. The supernatant was collect at 48 hpi and the virus titers in cell supernatant were measured. The results demonstrated that the gene encoding purkinje cell protein 4-like 1 (PCP4L1), which is a small neuronal IQ motif protein closely related to the calmodulin-binding protein PCP4/PEP-19 [63, 64], could significantly inhibit RABV replication (Fig. 7c). By transfecting different amounts of the plasmid expressing PCP4L1 in N2a cells, we found that PCP4L1 could inhibit RABV replication in a dose-dependent manner (Fig. 7d). To confirm that EDAL restricts viral replication via PCP4L1, we knocked down PCP4L1 with three different sets of siRNAs and then infected with RABV. The viral titer in the cell culture supernatant were upregulated around 3 folds post treatment with siPCP4L1-1 (Fig. 7e, f). Consistently, EDAL lost its antiviral ability post siPCP4L1-1 treatment in N2a cells (Fig. 7g). Furthermore, we found that PCP4L1 overexpression reduced the virus titers of VSV, SFV, and HSV-1 in N2a cells (Fig. 7h–j).

ChIP-seq results showed that the H3K27me3 level on the promoter region of *Pcp4l1* was dramatically decreased after EDAL expression (Fig. 7k), which was validated by ChIP-qPCR assay (Fig. 7l). After treatment with EZH2’s inhibitor gsk126, the transcriptional level of *Pcp4l1* was significantly increased, confirming that *Pcp4l1* transcription is regulated by EZH2 (Fig. 7m). All these results together suggest that EDAL might promote PCP4L1 expression by downregulating the EZH2-mediated H3K27me3 deposition.

## Discussion

We report here that multiple neurotropic viruses elicit the expression of a host lncRNA EDAL. EDAL inhibits the replication of RABV, VSV, SFV, and HSV-1 in neuronal cells and suppresses RABV infection in mouse brains. EDAL binds to the histone methyltransferase EZH2, a widely conserved epigenetic regulator, and specifically causes EZH2’s lysosomal degradation by blocking T309 *O*-GlcNAcylation. This in turn reduces cellular H3K27me3 levels. EDAL’s antiviral function resides in a 56-nt antiviral substructure that can fold into a tertiary structure with a 18-nt helix-loop that intimately contacts the T309 *O*-GlcNAcylation site of EZH2. Mutation analysis confirmed that EDAL’s effect on EZH2 lysosomal degradation requires the interaction between the 18 nt helix-loop of EDAL and EZH2 sites surrounding T309 *O*-GlcNAcylation, supporting that EDAL blocks a specific EZH2 PTM via tertiary interactions. Additionally, EDAL antiviral function could be attributed to its activated expression of a novel antiviral small peptide PCP4L1. Our discovery that neurotropic viruses elicit the expression of a neuronal antiviral lncRNA which facilitates the key epigenetic regulator EZH2 toward lysosomal degradation illustrates a way for a low level of lncRNA to effectively reduce the level of its target protein, as well as a direct biomolecular link among virus infection, host antiviral responses, and epigenetic regulation (Fig. 7n). The findings of the antiviral and EZH2 degradation function carried by a 56-nt segment of EDAL and its predicted capability of folding into a functional tertiary structure together highlight a mechanism for the specificity of lncRNA actions.

Recent studies have shown that post-translational modification (PTM) of EZH2 by phosphorylation affects its stability. CDK1 phosphorylates human EZH2 at T345 and T487, promoting ubiquitination of EZH2 and its subsequent degradation in proteasomes [40, 58]. T345 phosphorylation site is involved in regulating EZH2 binding with HOTAIR and XIST lncRNA [58]. K348 acetylation reduces the phosphorylation of EZH2 at T345 and T487 and increases the stability of EZH2 without interrupting PRC2 formation [65]. LncRNA ANCR facilitates the CDK1-EZH2 interaction and enhances the phosphorylation at T345 and T487, leading to EZH2 degradation and the attenuation of the invasion and metastasis of breast cancer [46].

It has been recently shown that *O*-GlcNAcylation catalyzed by OGT occurs at S73, S76, S84, T313, and S729 sites of the human EZH2, which does not affect the formation of the PRC2 complex. S76 and T313 are conserved in mammals, and S76A and T313A mutations independently reduce the stability of EZH2 [41, 42]. In the present study, molecular docking indicated that a 56-nt functional domain of EDAL lncRNA conveying both the antiviral and EZH2 degradation activity can shield T309 of mouse EZH2, the analogue of T313 in human EZH2, from the *O*-GlcNAcylation modification. PTM of biologically and therapeutically important proteins by *O*-GlcNAcylation are of interest both as lncRNA targets and therapeutic targets. *O*-GlcNAcylation is highly abundant in eukaryotes, occurring in both the nucleus and the cytoplasm [66–68]. In light of our confirmation of EDAL’s regulation of EZH2 *O*-GlcNAcylation, lncRNA regulation of other *O*-GlcNAcylation modification sites on other target regulatory (and other) proteins can be anticipated.

Note that EZH2-lncRNA interactions have been a popular model for studies of epigenetic silencing by PRC2 [28, 31, 55, 69–71]. However, the binding specificity of PRC2 for lncRNAs and other transcripts has been challenged and re-examined recently, leading to controversy about binding specificity and promiscuity [38, 39, 72]. Our findings indicated that EDAL binds to EZH2 at a site different from that of lncRNA-HOTAIR binding of human EZH2 via residues in 342–368 aa region [58]. More importantly, this study has shown that a 56-nt EDAL segment independently carries both the antiviral and EZH2 degradation function. Although we have not yet obtained structural data to support its predicted structure, our data for the function of the intimate contacts between the 18-nt helix-loop of EDAL and EZH2’s T309 *O*-GlcNAcylation site offers a new example of EZH2-lncRNA recognition and specificity.

DNA viral genome-encoded lncRNAs have recently been shown to actively interact with host epigenetic machinery to regulate both their own and host chromatin structure dynamics [73]. Some DNA viruses repress transcription and stabilize viral latency by methylating their host’s genomic DNA [74, 75]. In plants, both RNA and DNA viruses encode suppressors that limit the silencing capability of the host plants [76–79]. These silencing suppressors also reduce RNA-directed DNA methylation activity at transposons and repetitive sequences in the host genome, suggesting a potential regulatory role that plant viruses impose on their host epigenetic dynamics [77, 79, 80].

The present study reveals that neurotropic viruses elicits the expression of EDAL, a host cell lncRNA which restricts the replication of RABV, VSV, SFV, and HSV-1. We experimentally link EDAL’s antiviral activity to its function in decreasing the cellular stability of EZH2, a protein whose antiviral activity has been recently revealed against the DNA virus HSV-1 [36]. Consequently, we found that the cellular level of H3K27me3 marks was reduced in neuronal cells, which was accompanied by the removal of in the enriched H3K27me3 mark in an antiviral gene *Pcp4l1*. These findings suggest that viruses can elicit the expression of a host lncRNA which mediates EZH2 destabilization and reprograms host chromatin structure dynamics. This regulation could be anticipated during the infection by other RNA viruses and DNA virus as well. Alteration of the host epigenetic dynamics by virus-elicited host lncRNAs might not be limited to EZH2 and H3K27me3 mark. In *Drosophila*, the null mutants of the histone H3 lysine 9 methyltransferase G9a are more sensitive to RNA virus infection, and G9a controls the epigenetic state of immunity genes [81, 82]. It is thus possible that lncRNAs may be involved in G9a-regulated RNA virus responses.

PCP4L1 is a 68 aa polypeptide which display sequence similarity to the Purkinje Cell Protein 4 gene (*Pcp4*) and both of which are characterized by their C-terminal IQ domain ends [63]. PCP4L1 display a distinct expression pattern which is dominantly expressed in the CNS, and mostly expressed in circumventricular organs and modulate the production of the cerebrospinal fluid in the adult brain [63]. Previous studies showed that PCP4L1 may be a latent calmodulin-binding protein which becomes activated by post-translational modification [64]. Recently, PCP4L1 has been found to be involved in the development of diabetes and urinary bladder and colorectal cancer [83–85]. However, its role in suppressing pathogen infections has never been reported. Here we show the first evidence that PCP4L1 inhibits multiple neurotropic virus infection in neuronal cells. Moreover, our preliminary results revealed that PCP4L1 could be associated with RABV nucleoprotein (RABV-N) and resulted in the degradation of RABV-N, while the detailed mechanism is still under investigation.

In summary, our study of a major neurotropic virus reveals a previously unknown lncRNA-EZH2 PTM-mediated link between host antiviral responses and epigenetic regulation, and the involvement of a high specificity of lncRNA-protein tertiary interaction. The findings may reshape the current understanding of the lncRNA regulatory function, mechanism, and its partnership with EZH2. EZH2 is a promising anticancer target with a well-established oncogenic role in a large variety of cancers [34, 86]. The anticancer activities of a number of EZH2 inhibitor compounds have been reported [34, 61]. The exciting finding of the 56-nt RNA substructure carrying the full EZH2 inhibitor function not only offers an example of EZH2-lncRNA recognition and specificity, but also provides new opportunity for developing anticancer and antiviral therapeutics, as well as for developing molecular tracers of EZH2 to explore the cellular activity of EZH2 during its life time.

## Materials and methods

### Cell lines and viruses

Cell lines N2a (murine neuroblastoma N2a cells, ATCC® CCL-131), BSR (a clone of BHK-21, ATCC®CCL-10), C8-D1A (murine astrocytes, ATCC®CRL-2541), and Vero (*Cercopithecus aethiops* kidney cells, ATCC®CCL-81) were obtained from American Type Culture Collection (ATCC). BV2 (murine microglia, BNCC337749) were obtained from BeNa Culture Collection (BNCC). Cells were grown in a 37 °C humidified 5% CO_2_ atmosphere, and growth media was DMEM or RPMI1640 supplemented with 10% (vol/vol) FBS (Gibco) and 1% antibiotics (penicillin and streptomycin) (Beyotime). The recombinant rRABVs were cloned from RABV strain challenge virus standard-B2c (CVS-B2c) and constructed as described previously [53]. VSV is propagated in BHK-21 cells and stored in our lab. SFV and HSV-1 is a gift from Dr. Bo Zhang (Wuhan Institute of Virology, Chinese Academy of Sciences, Wuhan, China) and Dr. Gang Cao (Huazhong Agricultural University, China), respectively, both of which are propagated in Vero cells.

### Cell line authentication

Cell lines N2a (murine neuroblastoma N2a cells, ATCC® CCL-131), BSR (a clone of BHK-21, ATCC®CCL-10), C8-D1A (murine astrocytes, ATCC®CRL-2541), and Vero (Cercopithecus aethiops kidney cells, ATCC®CCL-81) were obtained from ATCC. BV2 (murine microglia, BNCC337749) were obtained from BNCC. Cell lines from ATCC or BNCC were authenticated by ATCC or BNCC and were not validated further in our laboratory. All cell lines used in this study have been regularly tested for potential mycoplasma contamination.

### Viral infection

Cells (N2a, BV2, C8-D1A and Vero) were infected with different rRABVs, VSV, SFV, or HSV-1 at a multiplicity of infection (MOI) of 0.01, 0.1, 1, or 3. After 1 h at 37 °C, the supernatant was discarded and cells were washed three times with PBS then cultured in DMEM or RPMI1640 supplemented with 2% (vol/vol) FBS (Gibco) and 1% antibiotics (penicillin and streptomycin, Beyotime) at 34 °C in a humidified 5% CO_2_ atmosphere.

### RNA-seq library construction, sequencing, and lncRNA prediction pipeline

Total RNA from RABV-infected N2a cells or mock-infected cells were isolated by using Trizol® reagent (Ambion) following the manufacturer’s instructions, and then treated with RQ1 DNase (Promega) to remove DNA. RNA quality and quantity were determined by measuring absorbance at 260 nm/280 nm (A260/A280) using a SmartSpec Plus spectrophotometer (BioRad). RNA integrity was verified by subjecting a sample of the RNA to electrophoresis in a 1.5% agarose gel.

Each RNA-seq library was prepared using 5 μg of total RNA. Polyadenylated mRNAs were purified and concentrated with oligo (dT)-conjugated magnetic beads (Invitrogen) and then used as templates for directional RNA-seq library preparation. Purified RNAs were iron fragmented at 95 °C, followed by end repair and 5′ adaptor ligation. Reverse transcription was performed using RT primers harboring a 3′ adaptor sequence and randomized hexamer. The cDNAs were purified and amplified by PCR, and products 200–500 bp in length were isolated, quantified, and used for sequencing.

For high-throughput sequencing, the libraries were prepared following the manufacturer’s instructions and analyzed using the Illumina NextSeq500 system for 150 nt pair-end sequencing.

### RNA-seq data processing and alignment

Raw reads containing more than two unknown (N) bases were discarded. Adaptors were removed from the remaining reads, and then short reads (less than 16 nt in length) and low-quality reads (containing more than 20 low quality bases) were also excluded by using the FASTX-Toolkit sequence processing pipeline (Version 0.0.13, http://hannonlab.cshl.edu/fastx_toolkit/) to yield the final data set (clean reads). The *Mus musculus* genome sequence (GRCm38) and annotation file (gencode.vM6 basic annotation) were obtained from the GENCODE database [87]. Clean reads were aligned end-to-end to the mouse genome by TopHat2 [88], allowing 2 mismatches. Reads that aligned to more than one genomic location were discarded, and uniquely localized reads were used to calculate the number of reads and RPKM values (RPKM represents reads per kilobase and per million) for each gene. Other statistics, such as gene coverage and depth, and read distribution around transcription start sites (TSSs) and transcription terminal sites (TTSs) were also obtained.

After calculating the expression levels for all genes in the samples, differentially expressed genes (DEGs) between samples were identified by edgeR [89] using the TMM normalization method [90]. For each gene, the fold changes, *p* values, and adjusted *p* values (FDR) were also determined by the edgeR package. Genes with FDR < 0.05 were classified as DEGs.

### LncRNA prediction pipeline

The lncRNA prediction pipeline was implemented following the methods described by Liu et al. [16]. The detailed descriptions of the prediction pipeline and filtering thresholds are as follows:

1. First, using the aligned RNA-seq data (see above), transcripts were assembled by Cufflinks V2.2.1 [47] using default parameters. After the initial assembly, transcripts with FPKM greater than or equal to 0.1 were subjected to a series of filters.
2. Cuffcompare (embedded in Cufflinks) was used to compare the transcripts with known mouse genes. Novel transcripts, including those that were intronic, intergenic, and antisense, were retained as candidate lncRNAs. Transcripts within 1000 bp of known coding genes were regarded as UTRs and discarded.
3. To remove potential protein-coding transcripts, coding potential score (CPS) was evaluated using the Coding Potential Calculator (CPC) [91]. CPC is a support vector machine-based classifier that assesses the protein-coding potential of transcripts based on six biologically meaningful sequence features. Transcripts with CPS scores below zero were regarded as non-coding RNAs.
4. Transcripts satisfying the above conditions, containing multiple exons and no fewer than 200 bases, or containing a single exon and no fewer than 1000 bases, were considered to be candidate lncRNAs.
5. We used Cuffmerge (from Cufflinks) to merge lncRNAs from all samples together to obtain the final lncRNA set. A total of 1662 novel lncRNA transcripts were identified, originating from 1377 lncRNA loci. The expression level of each lncRNA gene was recalculated, and antisense reads of lncRNAs were discarded.
6. Novel and known lncRNAs were combined into a single data set and subjected to analysis to identify differentially expressed lncRNA, using the same methods used to identify differentially expressed protein coding genes.

### Quantitative real-time PCR (qPCR)

Total RNA was isolated from cells and tissues by using TRIzol® reagent (Invitrogen). The genomic DNA was eliminated with TURBO DNA-free™ Kit (Invitrogen, AM1907) as the manufacturer’s instructions. RNA quality was assessed by using NanoDrop 2000 (Thermo Scientific). The cDNAs were synthesized by ReverTra Ace qPCR RT Master Mix (Toyobo, FSQ-201) or First-Strand cDNA Synthesis Kit (Toyobo, FSK-101). qPCR was performed using SYBR Green Supermix (Bio-Rad, 172-5124). For nuclear/cytoplasmic fractionation, N2a cells were treated with cytosol buffer containing 10 mM HEPES (pH 7.9), 1.5 mM MgCl_2_, 10 mM KCl, 1 mM EDTA, 1 mM DTT, 0.05% NP-40, and Rnase inhibitor (Thermo, EO0381), then left on ice for 15 min. The nuclei were then separated from the cytosolic fraction by centrifugation at 4 °C at 3000 rpm/min for 10 min. The cytosolic fraction at up layer and the nuclei pellet fraction at bottom were then treated with TRIzol® reagent for RNA isolation and qPCR analysis. Primer sequences used in this study were listed in Additional file 3: Table S3.

### Transfections

After seeding, cells were incubated for 12 h at 37 °C. Plasmids or siRNA were transfected into cells by using Lipofectamine 3000 (Invitrogen) according to the manufacturer’s instruction.

### Rapid amplification of cloned cDNA ends (RACE)

Total RNA from N2a cells was isolated by using Trizol® reagent (Invitrogen), and 5′- or 3′-RACE was performed with SMARTer®RACE 5′/3′ Kit (Takara, 634858) following the manufacturer’s instructions. Primers used for 5′- or 3′-RACE were designed based on the known sequence information. 5′ specific primer-GGGCTGGAGAAGTGGTTCCGTTGCTAAGGGTATTCCC; 3′ specific primer-1-GGGAATACCCTTAGCAACGGAACCACTTCTCCAGCC; 3′ specific primer-2-AGACTCCACGAGGACAACAGA.

### Ribosome-RNA complex isolation

Ribosome-RNA complex was isolated as previously reported [52]. Briefly, N2a cells were washed 3 times with PBS and treated with cycloheximide (100 μg/ml) for 2 min. Then the cells were harvested and lysed prior to size exclusion chromatography using MicroSpin S-400 HR Columns. The collected flow-through from the size exclusion column contains the ribosome-RNA complex. The RNA extracted from ribosome-RNA complex were then used to qPCR analyzing.

### Fluorescent in situ hybridization

The red fluorescence-labeled probe (Ribo-lncRNA FISH Probe Mix) against EDAL lncRNA or 18S was designed by Ribobio Co (Guangzhou, China) and was detected by Fluorescent In Situ Hybridization Kit (Ribobio, R11060.1) according to the manufacturer’s instructions. Briefly, N2a cells grown on cover slips in 24-well plates with indicated treatment were fixed with 4% (v/v) paraformaldehyde for 10 min (min) at room temperature then washed three times with cold PBS. And the cells were permeabilized in PBS containing 0.5% Triton X-100 for 5 min in 4 °C, then blocked in pre-hybridization buffer for 30 min at 37 °C. Cells were then incubated with a hybridization buffer-containing probe (2.5 μl, 20 μM probe in 250 μl hybridization buffer) overnight at 37 °C away from the light. After hybridization, cells were washed in the dark with washing buffer (4 × SSC/2 × SSC/1 × SSC) then stained with DAPI for 10 min. Cells were again washed three times with PBS, and then imaged with a ZEISS confocal microscope under oil objective.

### siRNAs

The specific siRNAs were designed and synthesized by Ribobio Co. To knock down the target genes, the final concentration of 50 nM siRNAs were transfected into N2a cells according to the manufacturer’s instruction.

EDAL-specific siRNAs: siEDAL-①: the target sequence was 5′-GGTAGACACCCAGTGACAA-3′, and siEDAL-①sequence was 5′-GGUAGACACCCAGUGACAA-3′; siEDAL-②: the target sequence was 5′-GGTGGCCTCAGATAGCTAA-3′, and siEDAL-② sequence was 5′-GCUCUUUACUGAUGAGCUA-3′; siEDAL-③: the target sequence was 5′-GCTCTTTACTGATGAGCTA-3′, and siEDAL-③ sequence was 5′-CCUACAGUUAAGAGACUUU-3′.

PCP4L1-specific siRNAs: siPcp4l1–1: the target sequence was 5′-GCTGGTAGTCACTAGGCTA-3′, and siPcp4l1–1 sequence was 5′-GCUGGUAGUCACUAGGCUA-3′; siPcp4l1–2: the target sequence was 5′-CCTAGTGCAGCTGCACTTT-3′, and siPcp4l1–2 sequence was 5′-CCUAGUGCAGCUGCACUUU-3′; siPcp4l1–3: the target sequence was 5′-CCAGCCTGGTTGACATCAT-3′, and siPcp4l1–3 sequence was 5′-CCAGCCUGGUUGACAUCAU-3′.

### Cell viability assay

N2a cells were transfected with plasmids and siRNAs or treated with EZH2 specific inhibitor gsk126 (Apexbio, A3446) for indicated time. The viability of N2a cells was evaluated by Cell Titer 96 AQueous One Solution cell proliferation assay kits (Promega, G3582) according to the manufacturer’s instruction.

### Construction of the recombinant RABVs (rRABV)

Mouse lncRNAs, reverse EDAL (revEDAL), were amplified from the total RNA extracted from RABV-infected N2a cells using the ReverTra Ace qPCR RT Master Mix (TOYOBO, FSQ-201) with Phanta Max Super-Fidelity DNA polymerase (Vazyme Biotech Co., Ltd, P505-d1). The primer sets used were designed by Primer 6 (PREMIER Biosoft Biolabs) (Additional file 3: Table S3). PCR products were digested with *BsiW*I and *Nhe*I (New England Biolabs) then ligated into the genome of recombinant RABV strain B2c (rB2c) digest which used the same enzymes as previously described [53].

### Rescue of rRABVs

Recombinant RABVs were rescued as reported previously [53]. Briefly, BSR cells were transfected with 2 μg of a fully infectious clone, 0.5 μg of pcDNA-N, 0.25 μg of pcDNA-P, 0.15 μg of pcDNA-G, and 0.1 μg of pcDNA-L using Lipo3000 transfection reagent (Invitrogen) according to the manufacturer’s instruction. Four days post transfection, supernatants were harvested and examined for the presence of rescued viruses using FITC-conjugated anti-RABV N antibodies (Fujirebio Diagnostics, Malvern, PA).

### Virus titration

To determine rRABV and VSV titers, BSR cells were infected with serial dilutions of the viruses. After 1 h incubation in 37 °C, the cell supernatant was discarded and washed once with PBS, and then overlaid with DMEM containing 1% low melting point agarose (VWR, 2787C340). After incubation in 34 °C for 72 h, the cells were stained with FITC-conjugated anti-RABV N antibody (Fujirebio Diagnostics, Malvern, PA). Then, the fluorescent foci were counted under a fluorescence microscope. For VSV titration, the plaques were counted at 48 h post infection.

For SFV and HSV-1 titration, Vero cells were seeded in 12-well plates and infected with serial dilutions of the viruses. After 1 h incubation in 37 °C, the cell supernatant was discarded and washed once with PBS, and then overlaid with DMEM containing 1% low melting point agarose. After incubation in 34 °C for 48 h, the agarose were removed and then fixed and stained with a solution of 0.1% crystal violet and 10% formalin in PBS under UV light. After staining for 4 h, the plates were washed with water, and the plaques were counted.

### Mouse infection

Eight-week-old female C57BL/6 mice were randomly divided into indicated groups and infected intranasally with rRABV, rRABV-EDAL, and rRABV-revEDAL (100 FFU) or mock infected with DMEM in a volume of 20 μl. When moribund, the mice were euthanized with CO_2_, and then the brains were collected for qPCR or immunohistochemistry analysis.

### Immunohistochemistry analysis

Groups of female C57BL/6 mice were infected intranasally with rRABV or rRABV-EDAL. At indicated times post infection (pi), mouse brains were harvested and fixed in 4% paraformaldehyde for 2 days at 4 °C. Tissues were then dehydrated in 30% sucrose in PBS for 48 h at 4 °C, then embedded in paraffin and sliced into 4-μm sections. For immunohistochemistry (IHC), the sections were deparaffinized and rehydrated in xylene and ethanol. Endogenous peroxidase was quenched by incubation in 3% hydrogen peroxide, and antigen retrieval was performed in 0.01 M citrate buffer. Sections were blocked then incubated with primary anti-RABV P antibody (prepared in our lab, 1:500) or CD45 antibody (Servicebio, GB11066, 1:3000) overnight at 4 °C. Sections were washed again then incubated with HRP-conjugated anti-mouse (Servicebio, G1211, without dilution) or anti-rabbit secondary antibodies (Servicebio, GB23303, 1:200). After washing, sections were incubated with diaminobenzidine (Servicebio, G1211) for color development then photographed and analyzed using an XSP-C204 microscope (CIC).

### Western blotting

N2a cells were lysed in RIPA buffer (Beyotime, P0013B) supplemented with 1× protease inhibitor cocktail (Roche). Total cell lysates were separated on 8–12% SDS-PAGE gels and transferred to PVDF membranes (Bio-Rad). Membranes were blocked with TBST with 5% (w/v) non-fat dry milk for 4 h and probed with primary antibodies which were diluted with TBST and 5% (w/v) non-fat dry milk overnight in 4 °C. The primary antibodies were against RABV N protein (prepared by our lab, 1:5000), H3K27me3 (Abclonal Technology, Wuhan, China, A2363, 1:2000), H3K4me3 (Abclonal, A2357, 1:2000), H3K36me3 (Abclonal, A2366, 1:2000), H3 (Abclonal, A2348, 1:2000), EZH2 (CST, #5246, 1:2000), Flag tag (MBL, M185-3 L, 1:10,000), HA tag (MBL, M180-3, 1:10,000), PCP4L1 (ProteinTech, 25933-1-AP, 1:2000), or GAPDH (ProteinTech, 60004-1-Ig, 1:5000). After rinsing, membranes were probed with HRP-conjugated goat anti-mouse (Boster, Wuan, China, BA1051), goat anti-rabbit secondary antibodies (Boster, BA1055, 1:6000), or goat anti-mouse IgG light-chain secondary antibodies (Abbkine, A25012, 1:5000), then developed using BeyoECL Star kit (Beyotime, P0018A). Images were captured with an Amersham Imager 600 (GE Healthcare) imaging system.

### Immunofluorescence analysis

N2a cells were transfected with indicated plasmids with Lipofectamine 3000 (Invitrogen) according to the manufacturer’s protocol. At 48 h post transfection, the cells were washed three times with cold PBS and fixed with 4% (v/v) paraformaldehyde for 10 min at room temperature then washed three times with cold PBS. And the cells were permeabilized in PBS containing 0.5% Triton X-100 for 5 min at 4 °C, then blocked in 10% goat serum which were diluted with PBS for 2 h at 37 °C, and probed with primary antibodies which were diluted with PBS and 5% (w/v) BSA for 2 h at 37 °C. The primary antibodies were against Flag tag (MBL, M185-3L, 1:500) and LAMP-1 (Abcam, Ab208943, 1:100), then treated with Alexa Fluor 594-conjugated anti-rabbit antibody (Invitrogen, A11012, 1:500) or Alexa Fluor 488-conjugated goat anti-mouse antibody (Invitrogen, R37120, 1:500) as a secondary antibody for 1 h at 37 °C, and then stained with DAPI for 10 min. Cells were again washed three times with PBS, and then imaged with a ZEISS confocal microscope under oil objective.

### Micrococcal nuclease footprinting sequencing

The DNA sequences of EDAL-1 and EDAL-98-153 were transcribed in vitro using T7 High Yield RNA Transcription kit (Vazyme Biotech Co., Ltd, TR101) and then purified with 4% agarose gel by using the Zymoclean™ Gel RNA Recovery Kit (Zymo Research, R1011). The RNA (5 ng) in 16 μl of 62.5 mM Tris–HCl (pH 7.5) was denatured at 95 °C for 1 min, annealed at 37 °C for 2 min, and then chilled on ice. Each sample was then allowed to fold at 37 °C for 5 min with 2 μl of 10× RNA folding buffer containing the desired concentrations of MgCl_2_ (5 mM or 0 mM), as we previously described [92, 93].

Micrococcal nuclease (Thermo, EN0181) (5.4 μl of 0.004 U/μl, total volume 30 μl) was then added to initiate the cleavage reaction, followed by incubation at 37 °C for 1 min. Cleavage was stopped by sequentially adding 480 μl DEPC water and 500 μl of phenol/chloroform/isoamyl alcohol (pH < 5.8), and immediately followed by vigorous vortexing. After centrifugation at 10,000*g*/min for 10 min, 450 μl of supernatant was removed and equal volume of loading buffer was added into the samples, and then the samples were stored at − 20 °C until electrophoresis.

After dephosphorylation and phosphorylation, all samples were used for small RNA cDNA library preparation with Balancer NGS Library Preparation Kit for small/microRNA (GnomeGen, K02420-L) following the manufacturer’s instruction. Briefly, RNAs were ligated to 3′ and 5′ adaptor sequentially and reverse transcribed to cDNA and then PCR amplified. Whole library was applied to 10% native PAGE gel electrophoresis, and bands corresponding to microRNA insertion were cut and eluted. After ethanol precipitation and washing, the purified small RNA libraries were quantified and stored at − 80 °C until sequencing.

For high-throughput sequencing, the libraries were prepared following the manufacturer’s instructions and applied to Illumina Novaseq 6000 system for 150 nt paired-end sequencing. Raw sequences from Novaseq 6000 were used to determine the 5′ and 3′ end for each read, which reflected the MNase cleavage site. Firstly, adaptors were removed from raw reads using cutadapt, and low-quality bases were trimmed using FASTX-Toolkit (version 0.0.13; http://hannonlab.cshl.edu/fastx_toolkit/index. html). Reads ≥ 5 nt were aligned to the EDAL RNAs using bowtie2. Aligned reads were filtered to obtain unique reads. Then read coverage of the two end points of all aligned reads were counted and sorted. The nucleotide sites of each RNA were partitioned into 5 groups according to the mapping signals (20% sites per group):open sites, less open sites, idle sites, and less protected sites from the highest to the lowest coverage. Protected site secondary structure models were generated using RNAfold (http://rna.tbi.univie.ac.at//cgi-bin/RNAWebSuite/RNAfold.cgi). Color coding by structure signal was done using VARNA (http://varna.lri.fr/).

### EDAL-EZH2 interaction 3D structure modeling

Murine EZH2 3D structure was predicted with SWISS-MODEL (https://swissmodel.expasy.org/interactive) based on human EZH2 3D structure (PDB code: 5HYN). Then amino acid sequence comparison was conducted between human EZH2 and Murine EZH2, and 98.24% similarity was calculated by Clustal2.1 (a multiple sequence alignment software, https://www.ebi.ac.uk/Tools/msa/muscle/). And the high sequence similarity ensures the authenticity of our predicted Murine EZH2 3D structure. EDAL-FD 3D structure model was predicted with RNAComposer (an automated RNA structure 3D modeling server, http://rnacomposer.ibch.poznan.pl/). In order to predict the interaction between EDAL functional domain (98–153 nt) and Murine EZH2, the template-based docking method PRIME [94] (If a template can be found, it is often more accurate than the free docking method) was used to dock the EDAL and EZH2 monomer structures at first. However, these two monomer structures could not find a suitable template in the template library, so the free docking method 3dRPC [95, 96] (A computational method was designed for 3D RNA-protein complex structure prediction.) was then utilized to dock EDAL and EZH2. Two atoms between EZH2 and EDAL with distance less than 5 Å in the predicted complex structure are considered to have interactions.

### RNA electrophoretic mobility shift assay (EMSA)

The overexpressed proteins were pulled down with anti-flag antibody and eluted with elution buffer (0.1 M Glycine, pH 3.0); the eluted proteins were then neutralized with neutralization buffer (1 M Tris, pH 8.5). Then the proteins were incubated with the cy5-labeled (5′) RNA (Synthesized by TSINGKE, Beijing, China) in the interaction buffer (30 mM NaCl, 5 mM Tris pH 7.5, 4 mM DTT, 0.04 mg/ml BSA, 2% glycerol) for 30 min at RT in the dark, and then the RNA-protein complex were loaded into 6.5% native gel to shift at a voltage of 130 V in Tris-Borate-EDTA (TBE) buffer (20 mM Tris pH 8.0, 50 mM boric acid, 1 mM sodium EDTA). The gels were then scanned with the FLA-2000 fluorescent image analyzer (Fuji, Stamford, CT).

### RNA pull-down assay

RNA was transcribed in vitro with T7 RNA polymerase (Roche, 10881767001) and labeled with Biotin RNA Labeling Mix (Roche, 11685597910). The synthesized RNA was treated with Rnase-free DNase I (Thermo, EN0521) and then purified with MicroElute RNA Clean-Up Kit (OMEGA, R6247-01). The RNA was heated to 95 °C for 2 min, put on ice for 5 min, and then put it at room temperature for 20 min to form a secondary structure. The cells were lysed with RNA immunoprecipitation (RIP) buffer containing 150 mM KCl, 25 mM Tris (pH 7.4), 0.5 mM DTT, 0.5% NP-40, 1 mM PMSF, and Rnase inhibitor (Thermo, EO0381), and then treated with Streptavidin M-280 beads (Thermo Fisher Scientific, 11205D) for 1 h and the lysed cells were collected to another tube for the next step. The prepared RNA was then added to the lysed cells containing the overexpressed proteins and incubated for 2 h at 4 °C. Then the Streptavidin M-280 beads were added to the protein-RNA mix and incubated for 1 h at room temperature. After being washed with wash buffer for three times, the samples were then analyzed by Western blotting.

### *O*-GlcNAcylation labeling and detection

The plasmid pCAGGS-EZH2-S73/S75/S725A-flag was co-transfected with pcDNA3.1, pcDNA-EDAL, or pcDNA-revEDAL in N2a cells and treated with 5 mM NH_4_Cl for 48 h. Then, the cells were lysed, and EZH2-S73/S75/S725A-flag was pulled down by anti-flag beads (MBL, M185-10). The extracted protein was labeled with Click-iT™ *O*-GlcNAc Enzymatic Labeling System (Invitrogen, C33368) following the manufacturer’s protocol. Then the *O*-GlcNAcylation level of the labeled EZH2-S73/S75-S725A-flag was analyzed by Click-iT™ Protein Analysis Detection Kits (Invitrogen, C33370).

### Chromatin immunoprecipitation sequencing (ChIP-seq) library construction and sequencing

Briefly, N2a cell were transfected with pcDNA3.1 or pcDNA-EDAL for 48 h, then the growth media of N2a cells was removed and cells were rinsed three times with cold PBS. Then cells were added with formaldehyde to a final concentration of 1% and incubated at room temperature for 10 min. To stop the cross-linking reaction, glycine was added into cells to a final concentration of 0.125 M. Cells were harvested into cold PBS by scraping and transferred into a 1.5 ml microcentrifuge tube. After centrifugation at 1000*g* for 5 min at 4 °C, the formaldehyde crosslinked cells were collected and resuspended in 1 ml nuclei lysis buffer (50 mM Tris-HCl pH 8.0, 10 mM EDTA pH 8.0, 1% SDS, 1 mM PMSF). Chromatin was sheared to an average size of 100–500 bp by sonication, and then centrifuged (10 min, 10,000*g*, 4 °C). In total, 60 μl of supernatant was diluted 10-fold with 540 μl ChIP dilution buffer (1% Triton X-100, 1.2 mM EDTA, 167 mM NaCl, and 16.7 mM Tris-HCl pH 8.0), then incubated with rotation with anti-H3K27me3 (Millipore, 07–449, 10 μg) or anti-rabbit IgG (Millipore, 12–370, 10 μg) overnight at 4 °C. Then, 50 μl protein A/G Dynabeads (Pierce™, #26162) were added to each sample and incubation continued for 2 h at 4 °C on a rotating platform. Beads were pelleted then washed sequentially with low salt buffer (150 mM NaCl, 20 mM Tris–HCl pH 8.0, 0.1% SDS, 0.5% Triton X-100, and 2 mM EDTA), high salt buffer (0.1% SDS, 1% Triton X-100, 2 mM EDTA, 20 mM Tris-HCl, pH 8.1, 500 mM NaCl), and LiCl buffer (0.25 M LiCl, 1% sodium deoxycholate, 10 mM Tris–HCl pH 8.0, 1% NP-40, and 1 mM EDTA), then twice with TE buffer (1 mM EDTA and 10 mM Tris–HCl pH 8.0). Chromatin was eluted from the beads by two washes with 100 μl elution buffer (100 mM NaHCO_3_, 1% SDS), the Na^+^ concentration was adjusted to 300 mM with 5 M NaCl and the crosslinks were reversed by overnight incubation in a 65 °C water-bath. Samples were then incubated with 0.1 mg/ml RNase A for 1 h at 37 °C, then with 1 mg/ml proteinase K for 2 h at 55 °C. DNA was purified by phenol extraction and ethanol precipitation. For high-throughput sequencing, the libraries were prepared following the manufacturer’s instructions (ThruPLEX DNA-seq 48S Kit, R400427) and analyzed using an Illumina NextSeq-500 system for 150 nt pair-end sequencing (ABlife Inc., Wuhan, China).

### ChIP-seq data analysis

Adaptors and low-quality bases were trimmed from raw sequencing reads using Cutadapt [97]. Reads were aligned to the mouse-GRCm38 genome using Bowtie2 [98]. To evaluate the quality of ChIP-seq data, we performed a cross-correlation analysis, as well as FRiP and IDR analyses for the ChIP-seq data, according to the ChIP-seq guidelines provided by the ENCODE and modENCODE consortia [99]. Peaks enriched by immunoprecipitation (compared to input DNA) were identified using MACS v1.4 [100]. We selected peaks with *p* values less than 10^− 5^. All peaks from each sample were clustered by BEDTools [101]. In this step, peaks with at least 1 bp overlap or book-ended features are merged. To associate peaks with genes, we set 10,000 bp as the upstream limit for the distance from the peak maximum to the TSS (transcript start site), and 3000 bp as the downstream limit for distance from the peak maximum to the TSS.

### ChIP-qPCR

Formaldehyde crosslinking of N2a cells, chromatin sonication, and immunoprecipitation were performed following the same procedures as the ChIP-seq section described above. The DNA pellet was suspended in 10 μl DEPC-water. Real-time PCR was then performed using a QuantStudio 6 Flex System (ABI) according to the manufacturer’s standard protocol. Input was used to normalize the amount of each sample as an internal control. Assays were repeated at least three times and expressed as Ct values. All PCR primer sequences can be found in Additional file 3: Table S3.

### Statistical analysis

Statistical analysis was performed using the R software (https://www.r-project.org/) or GraphPad Prism 6. Significance of differences was evaluated with either Student’s test, when only two groups were compared, or the hypergeometric test for Venn diagram. Survival percent was analyzed by the log rank test. Hierarchical clustering was performed by Cluster3.0 or heatmap function in R. No statistical method was used to predetermine sample sizes. *P < 0.05, **P < 0.01, and ***P < 0.001.

## Supplementary information

**Additional file 1: Supplemental Figure S1.** Sample correlation analysis. Hierarchical clustering heatmap shows global transcriptional changes after RABV infection. The Pearson correlation coefficients (PCCs) for each sample pair are represented using the colors in the color bar to indicate coefficient magnitude. Figure S2. EDAL transcriptome analysis. (Related to Fig. 1). a Read density of EDAL. The read density is based on normalized RNA-seq signals (TPM) for each sample after RABV infection. The nine tracks show RNA-seq read density at three time points after RABV infection, with three replicates per time point. Density is shown on the y-axis. b The RACE track shows the genomic location of long sequences ends detected by 5′ RACE (blue) and 3′ RACE (orange). The black rectangle indicates the predicted genomic location of EDAL by RNA-seq. The locus of 5′-RACE, 3′-RACE, and RT-qPCR primers were shown in EDAL. c The PhyloCSF score track shows negative protein-coding scores calculated by PhyloCSF. Scores below zero indicate non-coding features. The repeated masker track shows predicted repeat sequences. d The basal level of the target RNAs in RNA-seq (left). Ribosome-RNA complex was isolated from N2a cells, and the RNA copy numbers were quantified by qPCR (right). Malat1 was included as a noncoding RNA control, while Dennd1b and Crebrf were selected as the coding mRNA controls. (*n* = 3). e Conserved sequences in EDAL. Sequence analyses were performed using the UCSC genome browser. f RNA fluorescent in situ hybridization (FISH) assay were performed in N2a cell. 18S ribosomal RNA (18S) was included as a cytoplasmic control. Figure S3. EDAL is not up-regulated by RABV proteins, dsRNA, or interferons. (Related to Fig. 1). a N2a cells were infected with VSV at different MOIs for 12 h and EDAL level was analyzed by qPCR. b N2a cells were infected with SFV at different MOIs for 18 h and EDAL level was analyzed by qPCR. c N2a cells were infected with HSV-1 at different MOIs for 18 h and EDAL level was analyzed by qPCR. d N2a cells were transfected with plasmids expressing different RABV proteins. EDAL levels were analyzed by qPCR at 24 h post transfection. e N2a cells were transfected with poly(I:C) (a mimic of dsRNA) at indicated doses. EDAL levels were measured by qPCR at 24 h post transfection. f,g N2a cells were treated with IFN-β (f) or IFN-γ (g) for 24 h. EDAL levels were analyzed by qPCR. Statistical analysis of grouped comparisons was carried out by student’s t test (**P < 0.01; ***P < 0.001). Bar graph represents means ± SD, *n* = 3. Figure S4. Cell viability post overexpressing or silencing EDAL. (Related to Fig. 2). a EDAL was cloned into a mammalian expression vector pcDNA3.1, named pcDNA-EDAL. After transfection in N2a cells for 48 h, the expression of EDAL was evaluated by qPCR and FISH, respectively. b N2a cells were transfected with three different sets of EDAL specific siRNAs (siEDAL-①, ②, ③) or siNC for 72 h, then the expression of EDAL was evaluated by qPCR and FISH. c N2a cells were transfected with pcDNA3.1 or pcDNA-EDAL for indicated times, cell viability was evaluated using a Cell Titer 96 AQueous One Solution cell proliferation assay kits (G3582) from Promega. d N2a cells were transfected with siEDAL or siNC for indicated times, cell viability was measured. e N2a cells were pretreated with anti-IFN α/β receptor antibody (2 μg/ml) and then infected with rRABV, rRABV-EDAL or rRABV-revEDAL at MOI 0.01. At 48 dpi, virus titers in the cell culture were measured. Statistical analysis of grouped comparisons was carried out by student’s t test (*P < 0.05; **P < 0.01; ***P < 0.001). Bar graph represents means ± SD, *n* = 3. Figure S5. Micrococcal nuclease footprinting sequencing. (Related to Fig. 5) The secondary structures of the in vitro transcribed EDAL-1 (a) and EDAL-98-153 (b) were probed by micrococcal nuclease partial digestion and footprinting sequencing. The cleavage site represented by the two ends of a sequence read was recovered. The cleave intensity was mapped onto to the predicted secondary structures. The structures obtained from RNA folded in the absence (left panels) and presence (right panels) of 5 mM MgCl_2_ are shown. Figure S6. Amino acid sequence comparison between murine and human EZH2. (Related to Fig. 6). a The potential phosphorylation sites of murine EZH2 was mutated into A. Then the mutated EZH2 was expressed together with pcDNA3.1, pcDNA-EDAL or pcDNA-revEDAL in N2a cells for 48 h. Then EZH2-flag level was analyzed by Western blotting and normalized to H3. b The cell lysates were added with IgG or RL2 antibody and IP assays were performed. Then the EZH2 or EZH2-flag level were analyzed by Western blotting. c The amino acid sequence of murine and human EZH2 were aligned by using an online software ESPript3.0 (http://espript.ibcp.fr/ESPript/cgi-bin/ESPript.cgi). The *O*-GlcNAcylation sites and phosphorylation sites of human EZH2 were marked by O (*O*-GlcNAcylation) or P (phosphorylation), respectively. d N2a cells were transfected with the plasmids expressing WT EZH2, EZH2-S73/S75/S725A or EZH2-S73/S75/T309/S725A for 12 h and then treated with OGT inhibitor OSMI-1 for 36 h. At 48 h post transfection, the protein level was analyzed by Western blotting and normalized to H3. e N2a cells were transfected with the plasmids expressing WT EZH2, EZH2-S73/S75/S725A or EZH2-S73/S75/T309/S725A together with pCAGGS-OGT-HA in N2a cells for 48 h. Then EZH2-flag level was analyzed by Western blotting and normalized to H3. Figure S7. EZH2 specific inhibitor gsk126 inhibits RABV and VSV replication in N2a cells. a,b After treatment with different concentrations of gsk126, an EZH2 specific inhibitor, the viability of N2a cells was evaluated by using Cell Titer 96 AQueous One Solution cell proliferation assay kit (Promega, Madison, WI) (a). (n = 3) H3K27me3 levels were measured by Western blotting and normalized to H3 (b). c N2a cells were treated with 4 μM gsk126 or DMSO for 12 h, and then infected with rRABV at MOI 0.01. At 48 hpi, the virus load in the supernatant was titrated. d N2a cells were treated with 4 μM gsk126 or DMSO for 12 h, then infected with VSV at MOI 0.01 for 12 h, the virus load in the supernatant were measured. Statistical analysis of grouped comparisons was carried out by student’s t test (***P < 0.001). Bar graph represents means ± SD, *n* = 3.

**Additional file 2.** Supplemental Table S1.

**Additional file 3.** Supplemental Table S2. Sequencing and mapping information of ChIP-seq experiments. Each sample was tested in duplicates. Table S3. The primer sets used in this study. Table S4. The sequence of lncRNA EDAL expressed in this study.

**Additional file 4.** Complete western blot images of all figures in the manuscript are provided in additional file 4.

**Additional file 5.** Review history.

## Data Availability

The RNA-seq and ChIP-seq data reported in this study have been deposited in the Gene Expression Omnibus (GEO) under accession number GSE107310 [102]. The data which support the findings of this study are available from the corresponding author on reasonable request.
