# Supplementary material for: A novel antiviral lncRNA, EDAL, shields a T309 O-GlcNAcylation site to promote EZH2 lysosomal degradation
Source: Genome Biol. 2020 Sep 1;21:228. doi: 10.1186/s13059-020-02150-9 (PMC7465408; doi:10.1186/s13059-020-02150-9)
Supplement: Supplementary file 1 — Additional file 1: Supplemental Figure S1. Sample correlation analysis. Hierarchical clustering heatmap shows global transcriptional changes after RABV infection. The Pearson correlation coefficients (PCCs) for each sample pair are represented using the colors in the color bar to indicate coefficient magnitude. Figure S2. EDAL transcriptome analysis. (Related to Fig. 1). a Read density of EDAL. The read density is based on normalized RNA-seq signals (TPM) for each sample after RABV infection. The nine tracks show RNA-seq read density at three time points after RABV infection, with three replicates per time point. Density is shown on the y-axis. b The RACE track shows the genomic location of long sequences ends detected by 5′ RACE (blue) and 3′ RACE (orange). The black rectangle indicates the predicted genomic location of EDAL by RNA-seq. The locus of 5′-RACE, 3′-RACE, and RT-qPCR primers were shown in EDAL. c The PhyloCSF score track shows negative protein-coding scores calculated by PhyloCSF. Scores below zero indicate non-coding features. The repeated masker track shows predicted repeat sequences. d The basal level of the target RNAs in RNA-seq (left). Ribosome-RNA complex was isolated from N2a cells, and the RNA copy numbers were quantified by qPCR (right). Malat1 was included as a noncoding RNA control, while Dennd1b and Crebrf were selected as the coding mRNA controls. (n = 3). e Conserved sequences in EDAL. Sequence analyses were performed using the UCSC genome browser. f RNA fluorescent in situ hybridization (FISH) assay were performed in N2a cell. 18S ribosomal RNA (18S) was included as a cytoplasmic control. Figure S3. EDAL is not up-regulated by RABV proteins, dsRNA, or interferons. (Related to Fig. 1). a N2a cells were infected with VSV at different MOIs for 12 h and EDAL level was analyzed by qPCR. b N2a cells were infected with SFV at different MOIs for 18 h and EDAL level was analyzed by qPCR. c N2a cells were infected with HSV-1 at different MOIs for [file 13059_2020_2150_MOESM1_ESM.docx]

Additional files for

**A novel antiviral lncRNA, EDAL, shields a T309 *O*-GlcNAcylation site to promote EZH2 lysosomal degradation**

Baokun Sui, Dong Chen, Wei Liu, Qiong Wu, Bin Tian, Yingying Li, Jing Hou, Shiyong Liu, Juan Xie, Hao Jiang, Zhaochen Luo, Lei Lv, Fei Huang, Ruiming Li, Chengguang Zhang, Yuling Tian, Min Cui, Ming Zhou, Huanchun Chen, Zhen F. Fu, Yi Zhang, Ling Zhao

Corresponding authors:

Ling Zhao, Mailing address: State Key Laboratory of Agricultural Microbiology, Huazhong Agricultural University, Wuhan, 430070, China. E-mail: [zling604@yahoo.com](mailto:zling604@yahoo.com)

Yi Zhang, Mailing address: Center for Genome analysis and Laboratory for Genome Regulation and Human Health, ABLife Inc., Wuhan, 430075, China. E-mail: [yizhang@ablife.cc](mailto:yizhang@ablife.cc)

**This file includes:** Figures S1 to S7

**
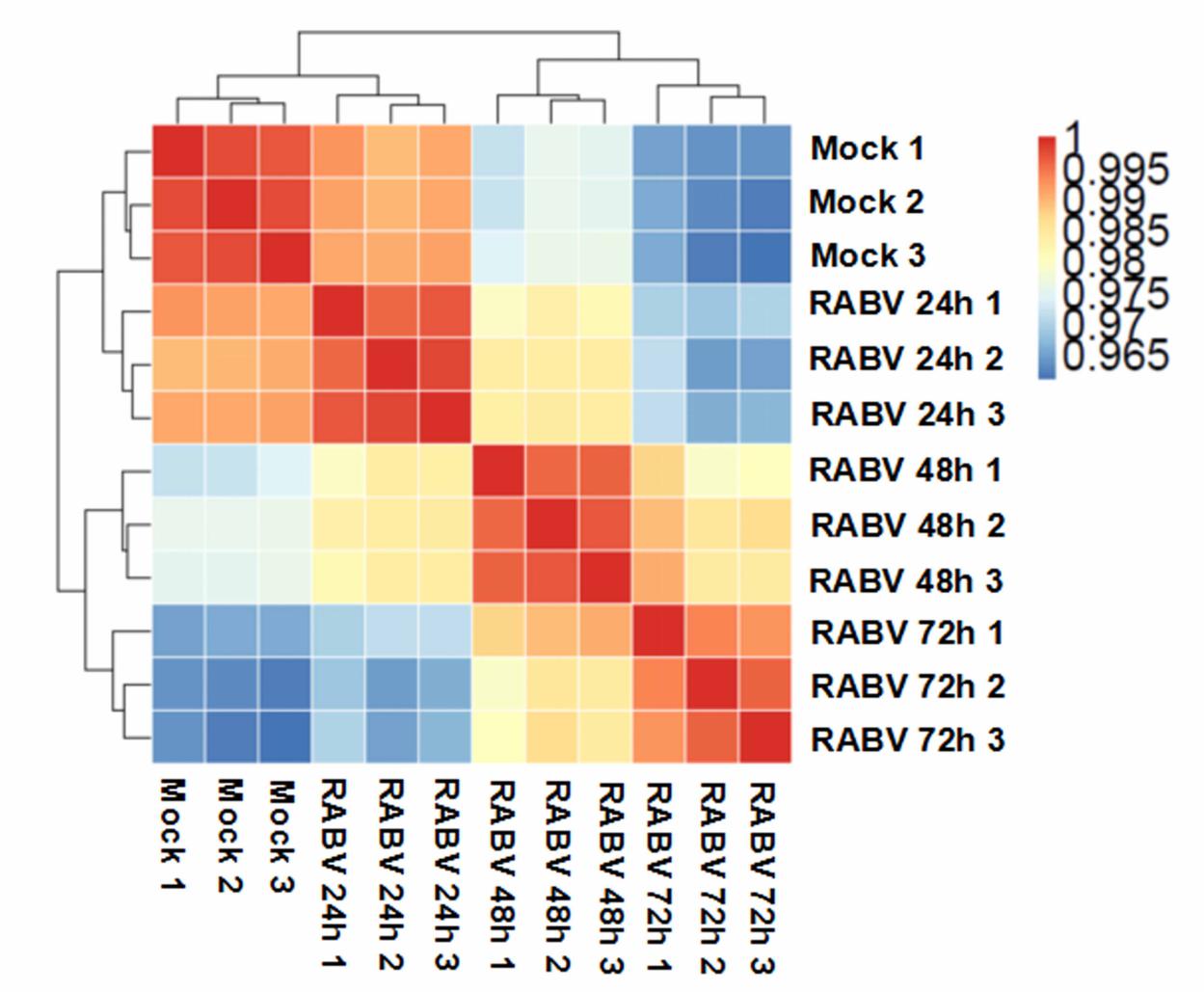
**

**Figure S1. Sample correlation analysis.** Hierarchical clustering heatmap shows global transcriptional changes after RABV infection. The Pearson correlation coefficients (PCCs) for each sample pair are represented using the colors in the color bar to indicate coefficient magnitude.


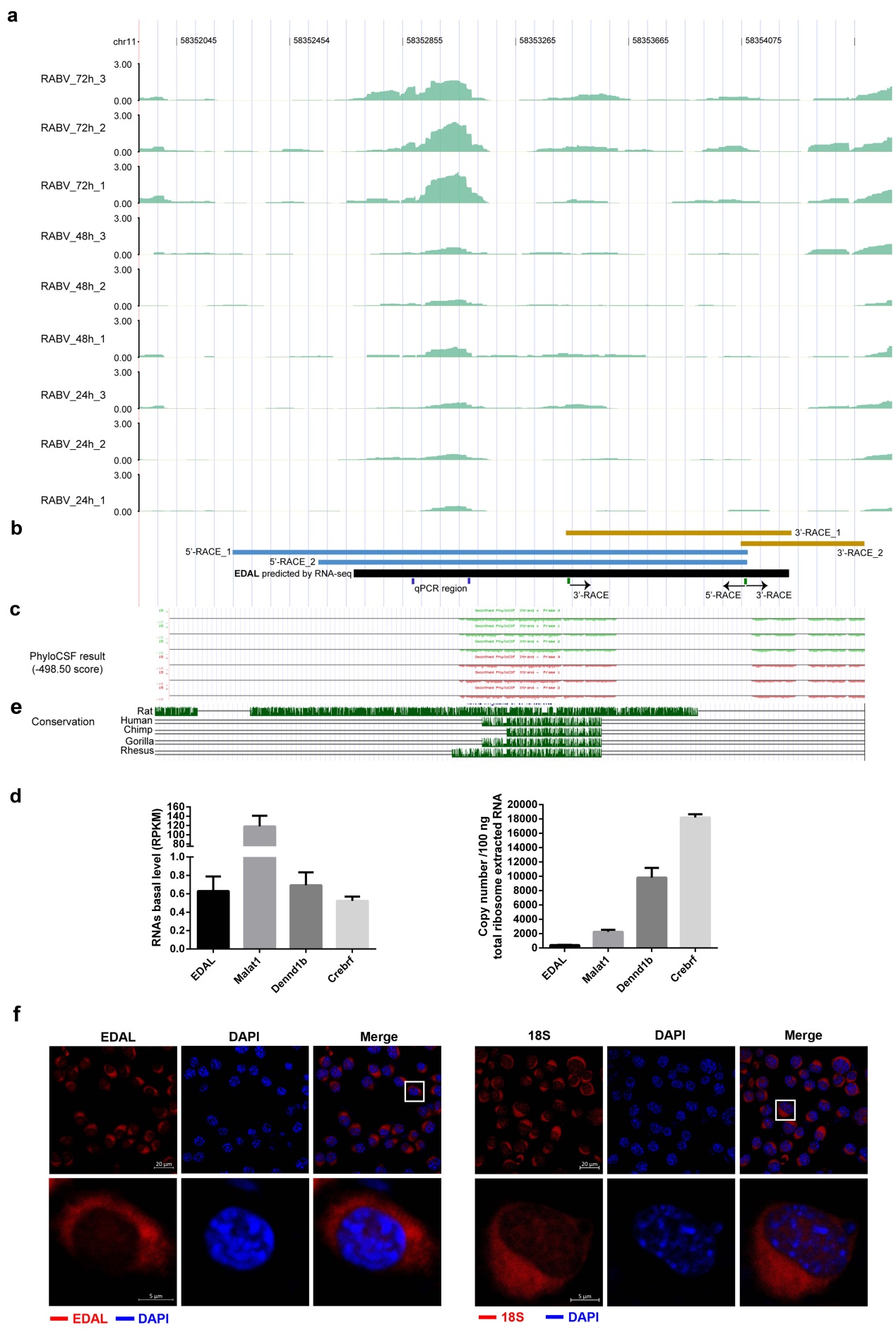


**Figure S2. EDAL transcriptome analysis. (Related to Fig. 1)**

**a** Read density of EDAL. The read density is based on normalized RNA-seq signals (TPM) for each sample after RABV infection. The nine tracks show RNA-seq read density at three time points after RABV infection, with three replicates per time point. Density is shown on the y-axis.

**b** The RACE track shows the genomic location of long sequences ends detected by 5’ RACE (blue) and 3’ RACE (orange). The black rectangle indicates the predicted genomic location of EDAL by RNA-seq. The locus of 5’-RACE, 3’-RACE, and RT-qPCR primers were shown in EDAL.

**c** The PhyloCSF score track shows negative protein-coding scores calculated by PhyloCSF. Scores below zero indicate non-coding features. The repeated masker track shows predicted repeat sequences.

**d** The basal level of the target RNAs in RNA-seq (left). Ribosome-RNA complex was isolated from N2a cells, and the RNA copy numbers were quantified by qPCR (right). Malat1 was inclued as a noncoding RNA control, while Dennd1b and Crebrf were selected as the coding mRNA controls. (n=3)

**e** Conserved sequences in EDAL. Sequence analyses were performed using the UCSC genome browser.

**f** RNA fluorescent *in situ* hybridization (FISH) assay were performed in N2a cell. 18S ribosomal RNA (18S) was included as a cytoplasmic control.


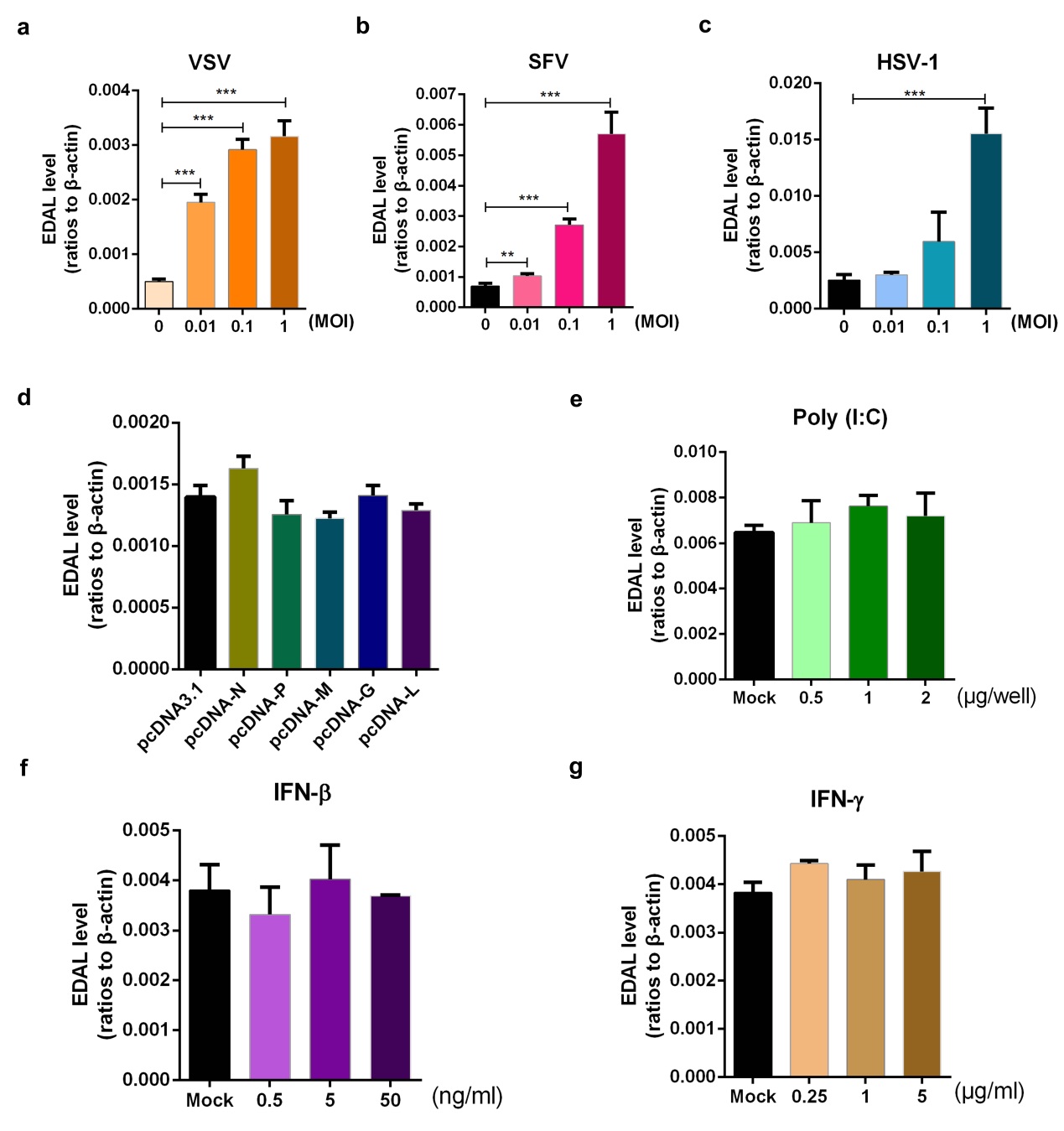


**Figure S3. EDAL is not up-regulated by RABV proteins, dsRNA, or interferons. (Related to Fig. 1)**

**a** N2a cells were infected with VSV at different MOIs for 12 h and EDAL level was analyzed by qPCR.

**b** N2a cells were infected with SFV at different MOIs for 18 h and EDAL level was analyzed by qPCR.

**c** N2a cells were infected with HSV-1 at different MOIs for 18 h and EDAL level was analyzed by qPCR.

**d** N2a cells were transfected with plasmids expressing different RABV proteins. EDAL levels were analyzed by qPCR at 24 h post transfection.

**e** N2a cells were transfected with poly(I:C) (a mimic of dsRNA) at indicated doses. EDAL levels were measured by qPCR at 24 h post transfection.

**f,g** N2a cells were treated with IFN-β (**f**) or IFN-γ (**g**) for 24 h. EDAL levels were analyzed by qPCR.

Statistical analysis of grouped comparisons was carried out by student’s t test (**P<0.01; ***P<0.001). Bar graph represents means ± SD, *n* = 3.


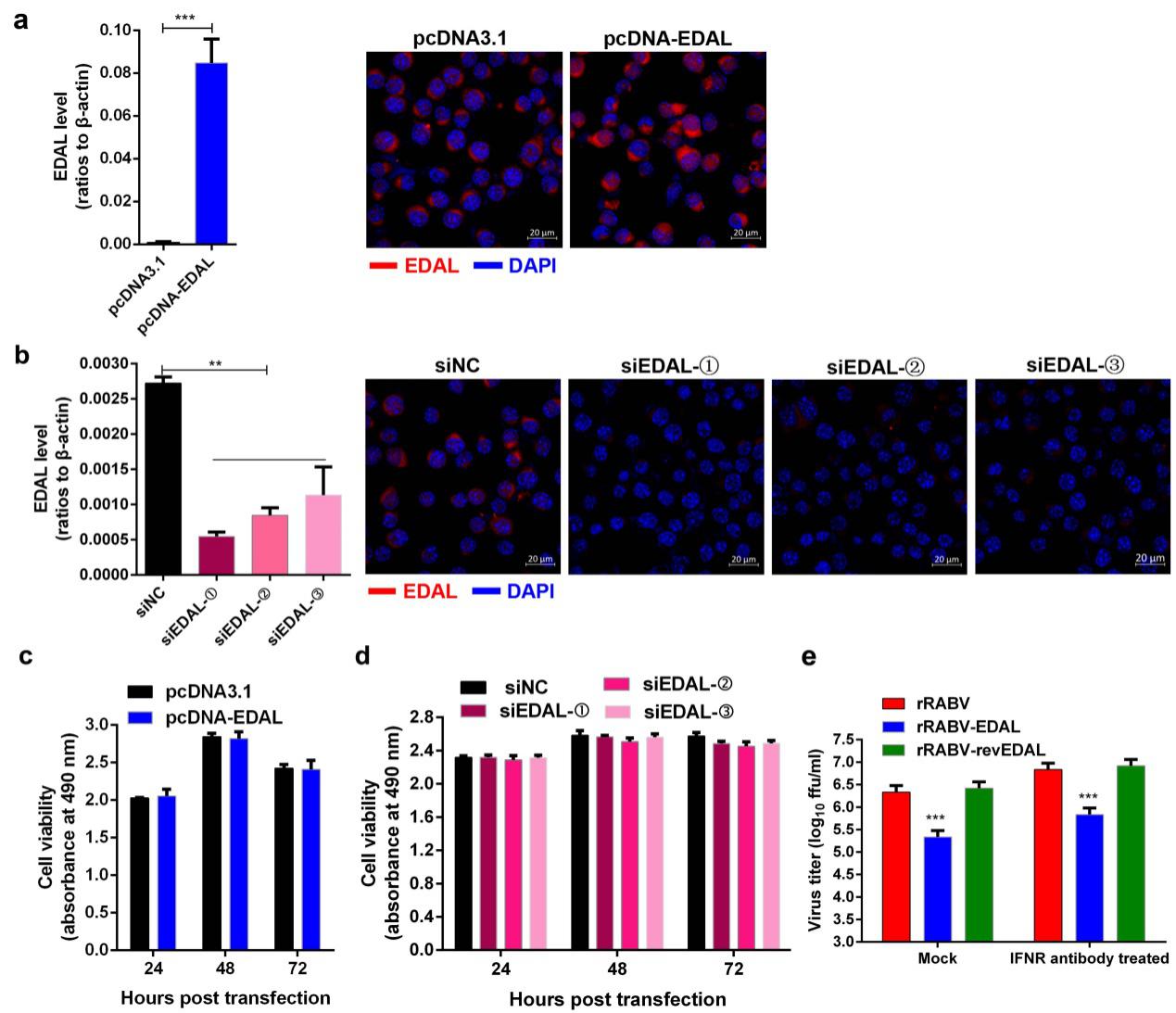


**Figure S4. Cell viability post overexpressing or silencing EDAL. (Related to Fig. 2)**

**a** EDAL was cloned into a mammalian expression vector pcDNA3.1, named pcDNA-EDAL. After transfection in N2a cells for 48 h, the expression of EDAL was evaluated by qPCR and FISH, respectively.

**b** N2a cells were transfected with three different sets of EDAL specific siRNAs (siEDAL-①, ②, ③) or siNC for 72 h, then the expression of EDAL was evaluated by qPCR and FISH.

**c** N2a cells were transfected with pcDNA3.1 or pcDNA-EDAL for indicated times, cell viability was evaluated using a Cell Titer 96 AQueous One Solution cell proliferation assay kits (G3582) from Promega.

**d** N2a cells were transfected with siEDAL or siNC for indicated times, cell viability was measured.

**e** N2a cells were pretreated with anti-IFN α/β receptor antibody (2 µg/ml) and then infected with rRABV, rRABV-EDAL or rRABV-revEDAL at MOI 0.01. At 48 dpi, virus titers in the cell culture were measured.

Statistical analysis of grouped comparisons was carried out by student’s t test (**P* < 0.05; **P<0.01; ***P<0.001). Bar graph represents means ± SD, *n* = 3.


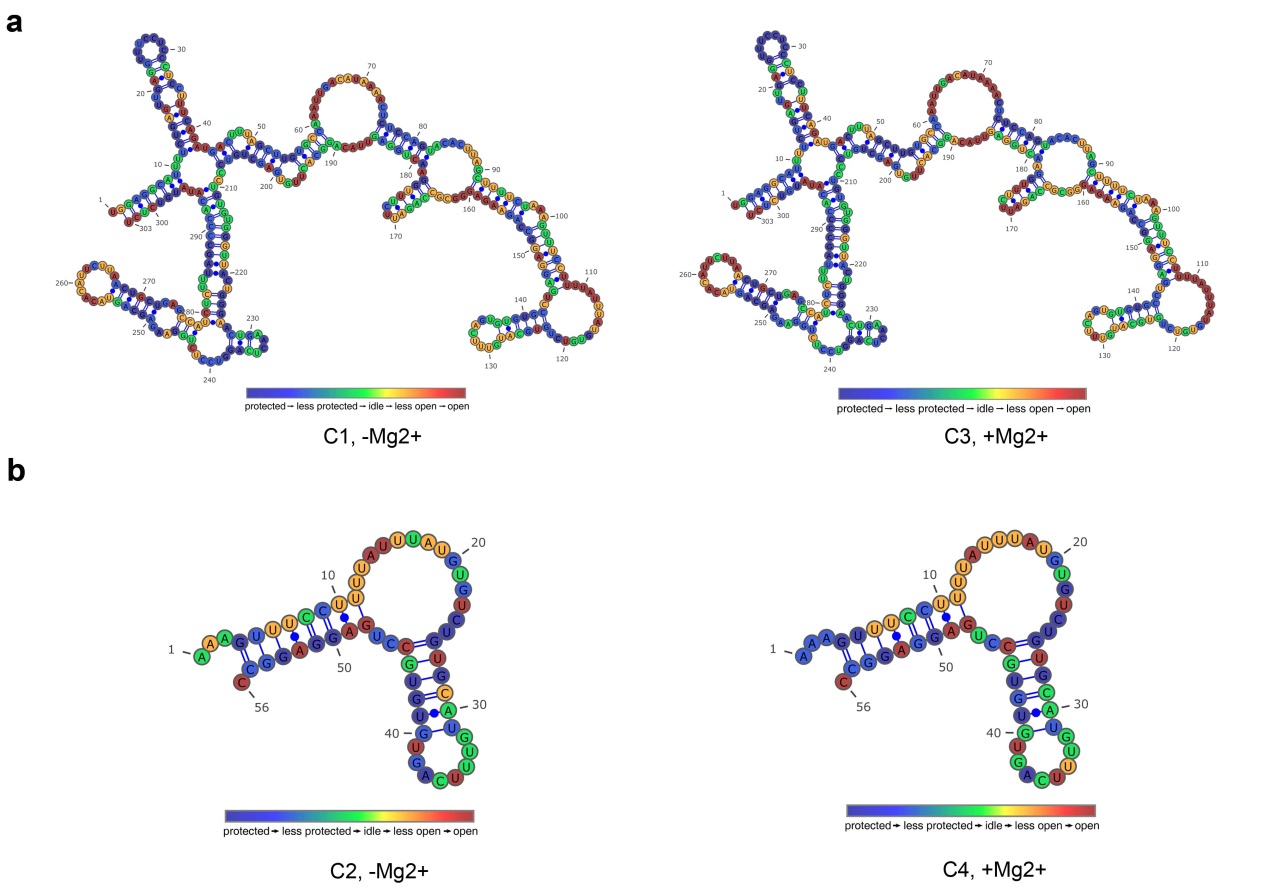


**Figure S5. Micrococcal nuclease footprinting sequencing. (Related to Fig. 5)** The secondary structures of the *in vitro* transcribed EDAL-1 (**a**) and EDAL-98-153 (**b**) were probed by micrococcal nuclease partial digestion and footprinting sequencing. The cleavage site represented by the two ends of a sequence read was recovered. The cleave intensity was mapped onto to the predicted secondary structures. The structures obtained from RNA folded in the absence (left panels) and presence (right panels) of 5 mM MgCl_2_ are shown.

**
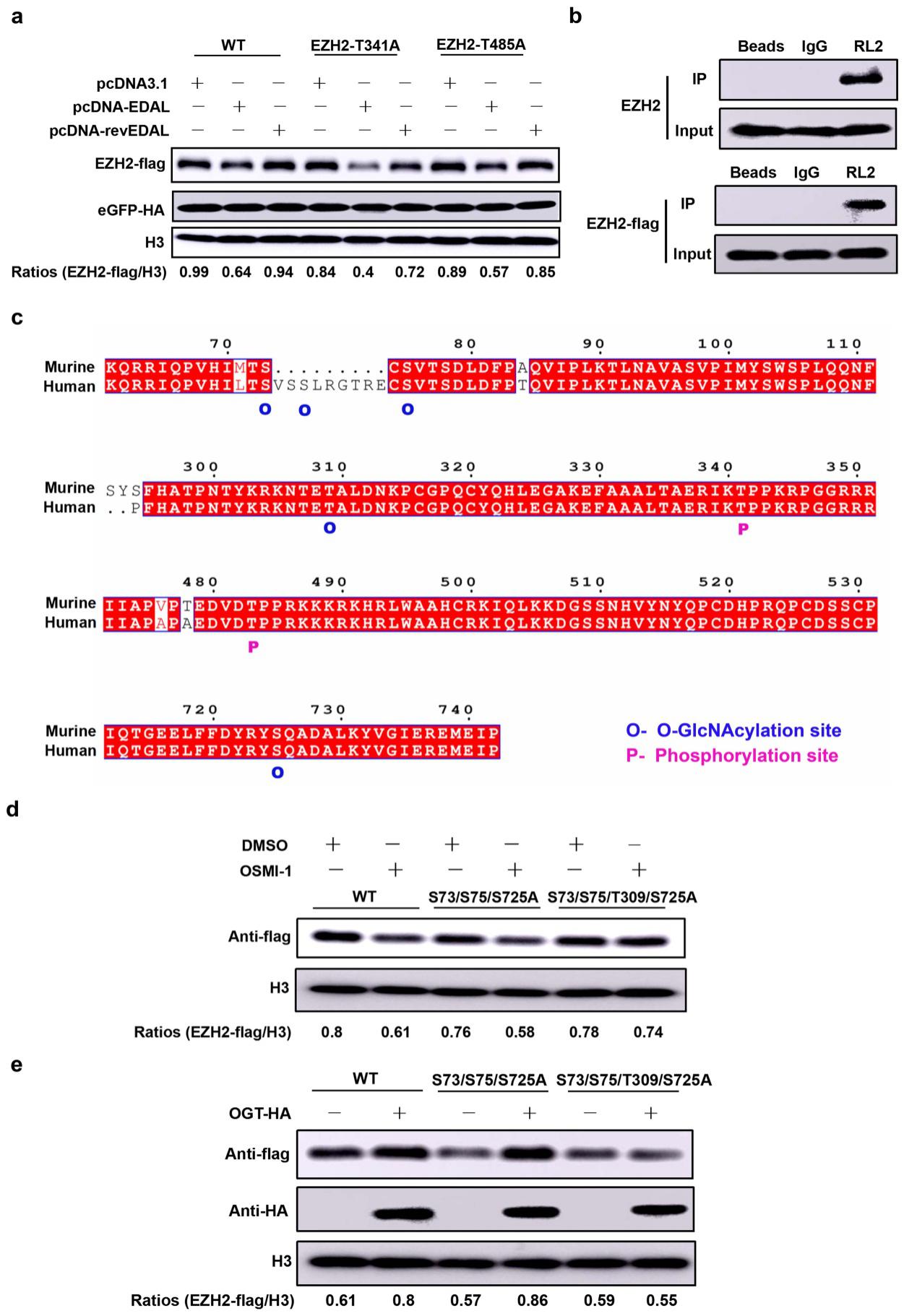
**

**Figure S6. Amino acid sequence comparison between murine and human EZH2. (Related to Fig. 6)**

**a** The potential phosphorylation sites of murine EZH2 was mutated into A. Then the mutated EZH2 was expressed together with pcDNA3.1, pcDNA-EDAL or pcDNA-revEDAL in N2a cells for 48 h. Then EZH2-flag level was analyzed by Western blotting and normalized to H3.

**b** The cell lysates were added with IgG or RL2 antibody and IP assays were performed. Then the EZH2 or EZH2-flag level were analyzed by Western blotting.

**c** The amino acid sequence of murine and human EZH2 were aligned by using an online software ESPript3.0 (<http://espript.ibcp.fr/ESPript/cgi-bin/ESPript.cgi>). The *O*-GlcNAcylation sites and phosphorylation sites of human EZH2 were marked by O (*O*-GlcNAcylation) or P (phosphorylation), respectively.

**d** N2a cells were transfected with the plasmids expressing WT EZH2, EZH2-S73/S75/S725A or EZH2-S73/S75/T309/S725A for 12 h and then treated with OGT inhibitor OSMI-1 for 36 h. At 48 h post transfection, the protein level was analyzed by Western blotting and normalized to H3.

**e** N2a cells were transfected with the plasmids expressing WT EZH2, EZH2-S73/S75/S725A or EZH2-S73/S75/T309/S725A together with pCAGGS-OGT-HA in N2a cells for 48 h. Then EZH2-flag level was analyzed by Western blotting and normalized to H3.


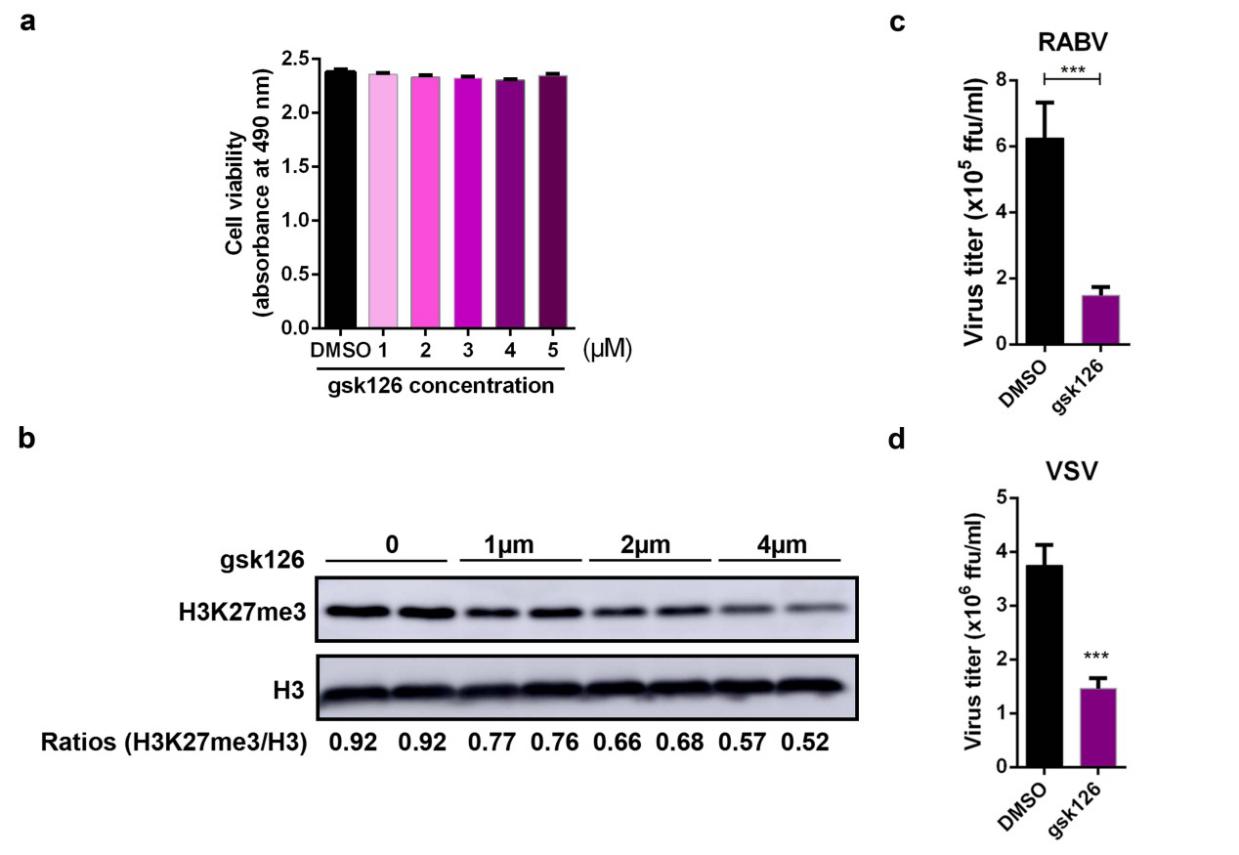


**Figure S7. EZH2 specific inhibitor gsk126 inhibits RABV and VSV replication in N2a cells.**

**a,b** After treatment with different concentrations of gsk126, an EZH2 specific inhibitor, the viability of N2a cells was evaluated by using Cell Titer 96 AQueous One Solution cell proliferation assay kit (Promega, Madison, WI) (**a**). (n=3) H3K27me3 levels were measured by Western blotting and normalized to H3 (**b**).

**c** N2a cells were treated with 4 µM gsk126 or DMSO for 12 h, and then infected with rRABV at MOI 0.01. At 48 hpi, the virus load in the supernatant was titrated.

**d** N2a cells were treated with 4 µM gsk126 or DMSO for 12 h, then infected with VSV at MOI 0.01 for 12 h, the virus load in the supernatant were measured.

Statistical analysis of grouped comparisons was carried out by student’s t test (***P<0.001). Bar graph represents means ± SD, *n* = 3.
