# Supplementary material for: A novel antiviral lncRNA, EDAL, shields a T309 O-GlcNAcylation site to promote EZH2 lysosomal degradation
Source: Genome Biol. 2020 Sep 1;21:228. doi: 10.1186/s13059-020-02150-9 (PMC7465408; doi:10.1186/s13059-020-02150-9)
Supplement: Supplementary file 3 — Additional file 3. Supplemental Table S2. Sequencing and mapping information of ChIP-seq experiments. Each sample was tested in duplicates. Table S3. The primer sets used in this study. Table S4. The sequence of lncRNA EDAL expressed in this study. [file 13059_2020_2150_MOESM3_ESM.docx]

Additional files for

**A novel antiviral lncRNA, EDAL, shields a T309 *O*-GlcNAcylation site to promote EZH2 lysosomal degradation**

Baokun Sui, Dong Chen, Wei Liu, Qiong Wu, Bin Tian, Yingying Li, Jing Hou, Shiyong Liu, Juan Xie, Hao Jiang, Zhaochen Luo, Lei Lv, Fei Huang, Ruiming Li, Chengguang Zhang, Yuling Tian, Min Cui, Ming Zhou, Huanchun Chen, Zhen F. Fu, Yi Zhang, Ling Zhao

Corresponding authors:

Ling Zhao, Mailing address: State Key Laboratory of Agricultural Microbiology, Huazhong Agricultural University, Wuhan, 430070, China. E-mail: [zling604@yahoo.com](mailto:zling604@yahoo.com)

Yi Zhang, Mailing address: Center for Genome analysis and Laboratory for Genome Regulation and Human Health, ABLife Inc., Wuhan, 430075, China. E-mail: [yizhang@ablife.cc](mailto:yizhang@ablife.cc)

**This file includes:** Tables S2 to S4

**Table S2.** Sequencing and mapping information of ChIP-seq experiments. Each sample was tested in duplicates.

| Sample | Raw reads | Reads after QC | total mapped reads | uniquely mapped reads | multiple mapped reads |
| --- | --- | --- | --- | --- | --- |
| pcDNA3.1_H3K27me3_1 | 69932858 | 55668777 | 52437993 (94.20%) | 39942525 (76.17%) | 12495468 (23.83%) |
| pcDNA3.1_H3K27me3_2 | 67891278 | 45142907 | 42187626 (93.45%) | 30301699 (71.83%) | 11885927 (28.17%) |
| pcDNA3.1_input_1 | 78994340 | 65958160 | 63686273 (96.56%) | 46843529 (73.55%) | 16842744 (26.45%) |
| pcDNA3.1_input_2 | 76318976 | 55917681 | 53343077 (95.40%) | 38161225 (71.54%) | 15181852 (28.46%) |
| pcDNA-EDAL_H3K27me3_1 | 73941712 | 58906209 | 55816652 (94.76%) | 40379662 (72.34%) | 15436990 (27.66%) |
| pcDNA-EDAL_H3K27me3_2 | 68085768 | 45952519 | 42358792 (92.18%) | 31024033 (73.24%) | 11334759 (26.76%) |
| pcDNA-EDAL_input_1 | 72335164 | 59681813 | 57346675 (96.09%) | 41376294 (72.15%) | 15970381 (27.85%) |
| pcDNA-EDAL_input_2 | 70565824 | 51660023 | 49080890 (95.01%) | 35607692 (72.55%) | 13473198 (27.45%) |

**Table S3.** The primer sets used in this study.

| qPCR primer name | Sequence(5’-3’) |
| --- | --- |
| XLOC_059122-F | GCTGTGGGGCATTTTCTCAA |
| XLOC_059122-R | AGCAGGTCAGGAATCAAGAG |
| ENSMUSG00000087684-F | CTGTGTCTTGGCTTGGGAGT |
| ENSMUSG00000087684-R | CCTGGGTGTTTCCTTTCTCA |
| XLOC_050868-F | GTCAGCCCTCTCTTTCCGCC |
| XLOC_050868-R | GCCTCCTGCTCTTCACGCTC |
| ENSMUSG00000085744-F | AGGGTCTCTGCCTGGAACT |
| ENSMUSG00000085744-R | AGTGGATGCTTTGTGAGG |
| XLOC_005934-F | AGTCTCCTGGGTGTTTGTGG |
| XLOC_005934-R | TGTGATGTCCCCTTGTGATG |
| XLOC_023040-F | CCAGTTTGGGAGGGGAGGAC |
| XLOC_023040-R | ATGGGTGTTGCGGATGGTG |
| XLOC_026667-F | AAATGGAAACCGAGGGTGGG |
| XLOC_026667-R | ATTGAGGGGCTGGGATGTGA |
| XLOC_047835-F | GGGAACCAGAGACAACGGGA |
| XLOC_047835-R | GCTGCTCCTGCCCACCATT |
| XLOC_058596-F | AGTAGGGCAGTGTTTGGCAC |
| XLOC_058596-R | GGCAGGTGGATTTCTGAGTT |
| Mouse-Crebrf-F | AGCAGAGAGTGGCAGCCTTT |
| Mouse-Crebrf-R | TGGCTGTTCTCTCATGTTGTCA |
| Mouse-Dennd1b-F | TTGCAAAGAGTCACGCCAGA |
| Mouse-Dennd1b-R | CTGCACCAAACCAGAACAGG |
| Mouse-18S-F | TATGGTTCCTTTGGTCGCTCG |
| Mouse-18S-R | GATCTGATAAATGCACGCATCC |
| Mouse-Malat1-F | GGCGGAATTGCTGGTAGTTT |
| Mouse-Malat1-R | AGCATAGCAGTACACGCCTT |
| Mouse β-Actin-F | CACTGCCGCATCCTCTTCCTCCC |
| Mouse β-Actin-R | CAATAGTGATGACCTGGCCGT |
| Mouse EDAL-F | GTCCCTGTGTGGGTTACTGG |
| Mouse EDAL-R | TGGGGCTTACTTCCTTTCTG |
| RABV N mRNA-F | GATCGTGGAACACCATACCC |
| RABV N mRNA-R | TTCATAAGCGGTGACGACTG |
| Mouse Pcp4l1-F | ACACCAAAACACCTCCAGCA |
| Mouse Pcp4l1-R | CCTCCTCGGCCTTCTTGATG |

| ChIP-qPCR primer name | Sequence(5’-3’) |
| --- | --- |
| Mouse Pcp4l1-F | TCCCGCTCTCTCCGTCTTA |
| Mouse Pcp4l1-R | GCCTCCAGCCCAACCAATA |

| Primers for PCR | Sequence(5’-3’) |
| --- | --- |
| PpcDNA-EDAL-F | CTCACTATAGGGAGACCCAAGCTGGCTAGCTGGAGGCATTTTCTGAG |
| PpcDNA-EDAL-R | CGAGGCTGATCAGCGGGTTTAAACGGGCCCTGTGTTTGTTAAAATAC |
| PpcDNA-XLOC_059122-F | CTCACTATAGGGAGACCCAAGCTGGCTAGCCAATCCCCAATCTGTAG |
| PpcDNA-XLOC_059122-R | CGAGGCTGATCAGCGGGTTTAAACGGGCCCCTAACTGAGGAAATGCC |
| PpcDNA-ENSMUSG00000085744-F | CTCACTATAGGGAGACCCAAGCTGGCTAGCCCACATACTGAATCTGA |
| PpcDNA-ENSMUSG00000085744-R | CGAGGCTGATCAGCGGGTTTAAACGGGCCCCCGCCTTGGGGGCATAT |
| PpcDNA-XLOC_23040-F | CTCACTATAGGGAGACCCAAGCTGGCTAGCTCTATGTGAGGACACTTC |
| PpcDNA-XLOC_23040-R | CGAGGCTGATCAGCGGGTTTAAACGGGCCCTGCTCTGAAGCCTATGAA |
| PpcDNA-ENSMUSG00000103464.1-F | CTCACTATAGGGAGACCCAAGCTGGCTAGCGTTCAATAAAACTTTGGT |
| PpcDNA-ENSMUSG00000103464.1-R | CGAGGCTGATCAGCGGGTTTAAACGGGCCCCCGCGGCAAAAGCTTTAT |
| PpcDNA-ENSMUSG00000087590.2-F | CTCACTATAGGGAGACCCAAGCTGGCTAGCTTTCTATGCTCGCACGCA |
| PpcDNA-ENSMUSG00000087590.2-R | CGAGGCTGATCAGCGGGTTTAAACGGGCCCGAACAGCACATCGAAGCA |
| PrRABV-EDAL-F | CATGAAAAAAACTAACACTCCTCCCGTACGTGGAGGCATTTTCTGAG |
| PrRABV-EDAL-R | TACAGTTTTTTTCTCGACTGAAATGCTAGCTGTGTTTGTTAAAATAC |
| PrRABV-reEDAL-F | CATGAAAAAAACTAACACTCCTCCCGTACGTGTGTTTGTTAAAATAC |
| PrRABV-reEDAL-R | TACAGTTTTTTTCTCGACTGAAATGCTAGCTGGAGGCATTTTCTGAG |
| PpCAGGS-EZH2-flag-F | GCCACCATGGACTACAAAGACGATGACGACAAGGGCCAGACTGGGAAG |
| PpCAGGS-EZH2-flag-R | CTCGAGTTACTTGTCGTCATCGTCTTTGTAGTCAGGGATTTCCATTTC |
| PpCAGGS-Wnt6-F | TTGTGCTGTCTCATCATTTTGGCAAAGAATTCGCCACCATGCTGCCGCCGGTGCCCTC |
| PpCAGGS-Wnt6-R | TGGCAGAGGGAAAAAGATCTGCTAGCTCGAGTTAGAGGCACAGGCTGAGTT |
| PpCAGGS-Foxn4-F | TTGTGCTGTCTCATCATTTTGGCAAAGAATTCGCCACCATGATAGAAAGTGGCATTTG |
| PpCAGGS-Foxn4-R | TGGCAGAGGGAAAAAGATCTGCTAGCTCGAGTTAAAGCAGAGCAATAGGCT |
| PpCAGGS-Ccbl2-F | TTGTGCTGTCTCATCATTTTGGCAAAGAATTCGCCACCATGCTTTTGGCCCAGAGGAG |
| PpCAGGS-Ccbl2-R | TGGCAGAGGGAAAAAGATCTGCTAGCTCGAGTTAAGACTTCTGGCTGTTCC |
| PpCAGGS-Fmo5-F | TTGTGCTGTCTCATCATTTTGGCAAAGAATTCGCCACCATGGCCAAAAAAAGGATTGC |
| PpCAGGS-Fmo5-R | TGGCAGAGGGAAAAAGATCTGCTAGCTCGAGTTAAAAATAAGCCAGGATGAC |
| PpCAGGS-Matn2-F | TTGTGCTGTCTCATCATTTTGGCAAAGAATTCGCCACCATGGAGAAGATGTTGGTGGGG |
| PpCAGGS-Matn2-R | TGGCAGAGGGAAAAAGATCTGCTAGCTCGAGTTATCTGTATTTTAGGCGATT |
| PpCAGGS-Pcp4l1-F | GAATTCGCCACCATGAGCGAGCTTAACACCAAAAC |
| PpCAGGS-Pcp4l1-R | CTGCTAGCTCGAGTTAGGAGCTGGAATCCTTTTTCC |

**Table S4.** The sequence of lncRNA EDAL expressed in this study.

| TGGAGGCATTTTCTGAGTTGAGGTTTCCTCCCTCCTTTCAGATGACTTTAGCTTGTGCCAAATTGACATAAAACTCTCCAGTACACTTAGCTTTTCTAAAGTTTCCTTTTATTTATGTGTCTGTGCATGTTTCAGTGTGTGCCTGAGGAGGCCAGAAGAGGGCGCCAGATTCTTTGGAACTGGAGTTACAGGCACTTGTGAGCT***GTCCCTGTGTGGGTTACTGG***GAACTGAACTCAGGTCCTCTGGAAGAGCAGTACACATTCTTAACTGCTGAGCCATCTCTTTAGCCCCACATATGTCTCTTTGGTTGAAATATTAAGCTTGGGGTAGTAGAGCCTGGATGCTTGAGGCTATCTGGCTCTGGCTTGATTGAACTCTAAAAACAAAAGAACCTCTGTATAGCAG***CAGAAAGGAAGTAAGCCCCA***AGATATGTGTGATTAGCTGGTAGAGGGCTCCTTAGGGAAAGAAGAGGTAGACACCCAGTGACAAGTGGTATAAAGGGTGCCCAGAAGTACTGACTGGTATTGTGCACAGCCTCGCTAGCTTTATCACCCTTGCTTATCTTAACCTGGCTTATCTTGACCCGCCTTAGGTTTCTAGGTGTGGGGCTGTTCCTGGGGCTCACTGACCTCCTCAGCTGTTGACAGACAGAAGGTTTATGGATCCAGTGTCTGTGAAATCACTGATAAATGGAGTTTTAGACAGACCCCACCCTTGGTTGGCTCCGGTGGCCTCAGATAGCTAACCCTGTAACGGGAAGCCAGACTCCACGAGGACAACAGAGTATGCAGCTCTCCAGAAGGCATGTTCACATGGCTGGAACGTTTTTTAAAGTCTTCTCTGGAAGGCATGCTCTTTAGGAAGAAGACCAGGAGAGTGGGCCTGAGGATTTGCCCGTGAAACTCAGCCAGCTCAGAAAAAGCCATTGGCTAGTTAAAATGCAGTTGCCCAGTGTAGATTTTTTACTTGCTATTTTATTATTTTATTTGATGCGCATGGGCATTTTTGTCTGCATGTATATCTGTGCACCATGTGTGAGTAGTGTCCAGGAGGTCAGAAGAAGGTGTTGGACCCCCTGGAACTGGATTACAGATGGCTGTTTCCATGCAGGTGATGGGAGCTGTACCCAGGTTCCCTGCAAAAGCAGCCGGTGCTCTTTACTGATGAGCTATTTCCTCAGCCCCTTCACTTATGATTTAGTTAGAAAAGGTCTGCCTGTCTGCCTGTCTGCCTGCCTGCCTGTCTGTCTGTGTTGGGTAATATGTACACGAGCATGTGTGCTTTCAGAGGCCAGAAGAAGTCACTGTGTGTCCAGAAGCTGGAGTTGGAGGCATTTGTGATGTGCCTGGTGTGGGTTCTGAGGACCTAAGCAGGGTCCTCTGCAAGAGCAGGGAATACCCTTAGCAACGGAACCACTTCTCCAGCCCCTTACTGGTTTTTGTTTTGTTTTTAATGGAATATATTGGATGTCTCCACAATTATGTTCAGGTGACTGCAGAGCCTGGAAATTGCTGCTGTAATTGATACAGAACATACTAGTGTTCTTGGTATTTTAACAAACACA |
| --- |

The sequences in italics show the qPCR primer pairs.
