# Supplementary figures and images for: A novel antiviral lncRNA, EDAL, shields a T309 O-GlcNAcylation site to promote EZH2 lysosomal degradation
Source: Genome Biol. 2020 Sep 1;21:228. doi: 10.1186/s13059-020-02150-9 (PMC7465408; doi:10.1186/s13059-020-02150-9)

**Complete images for all Western blots**


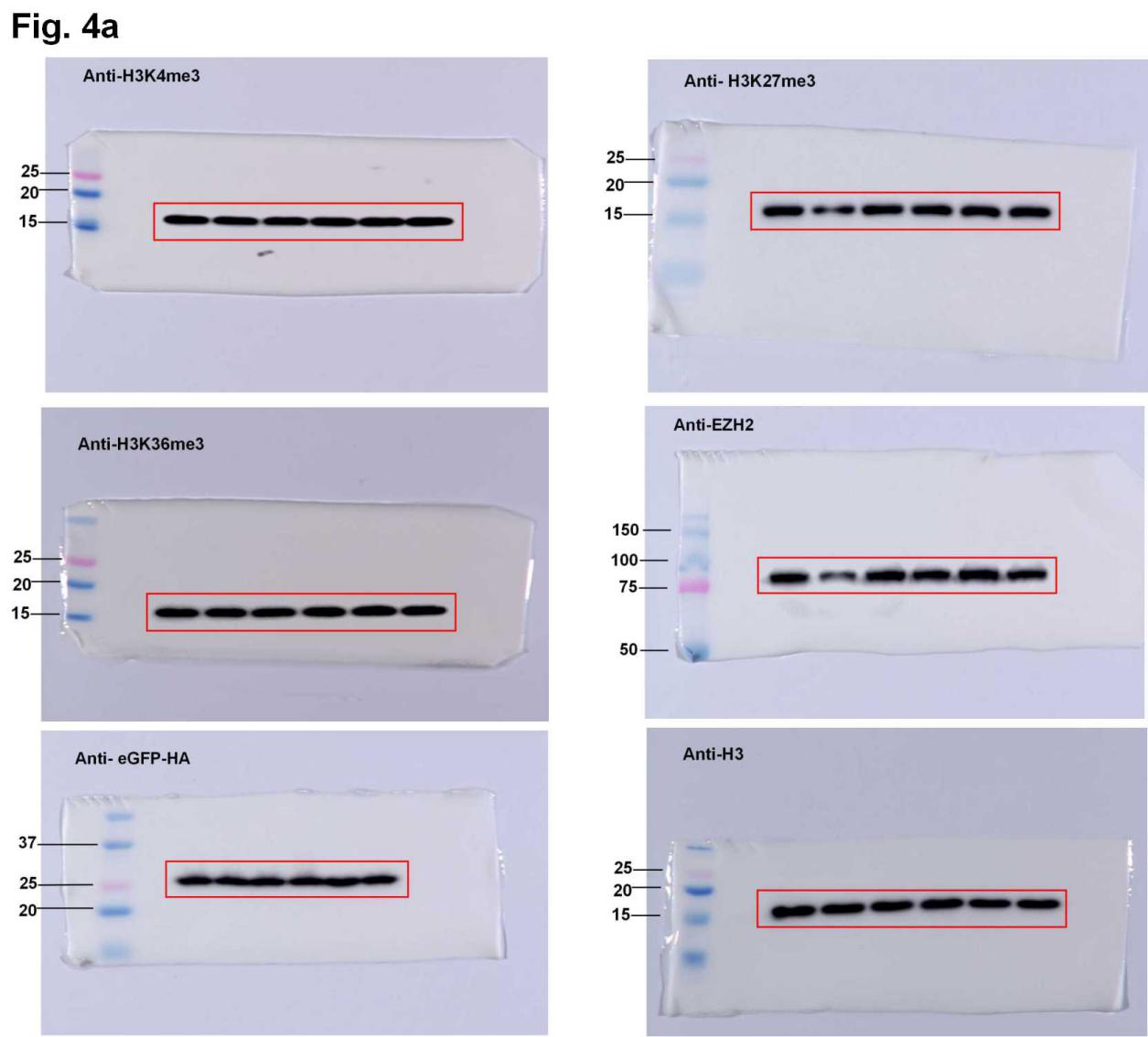


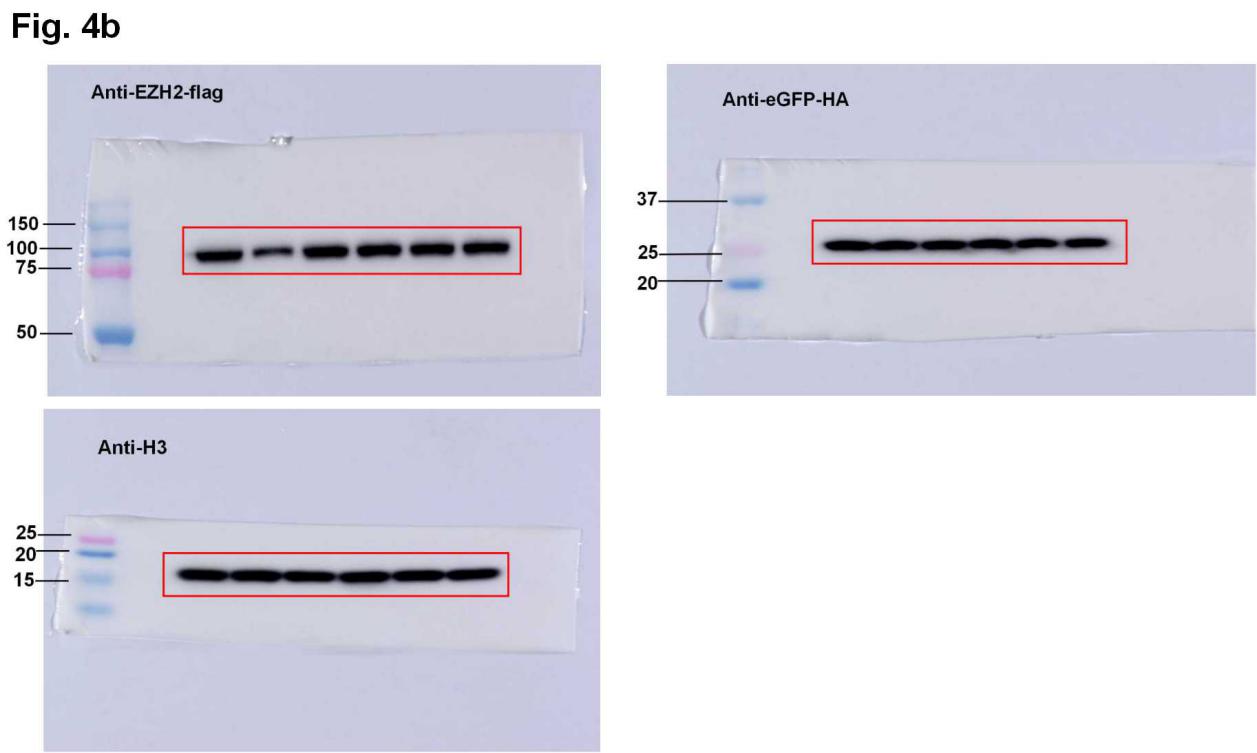


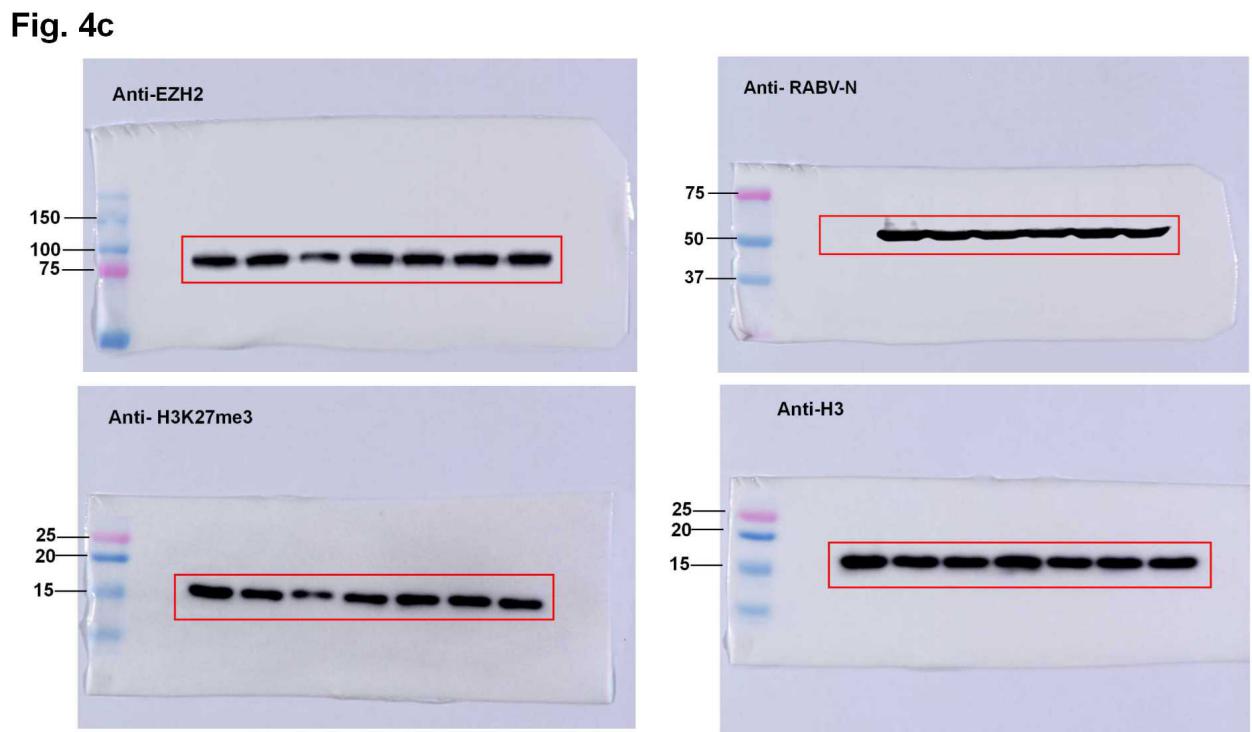


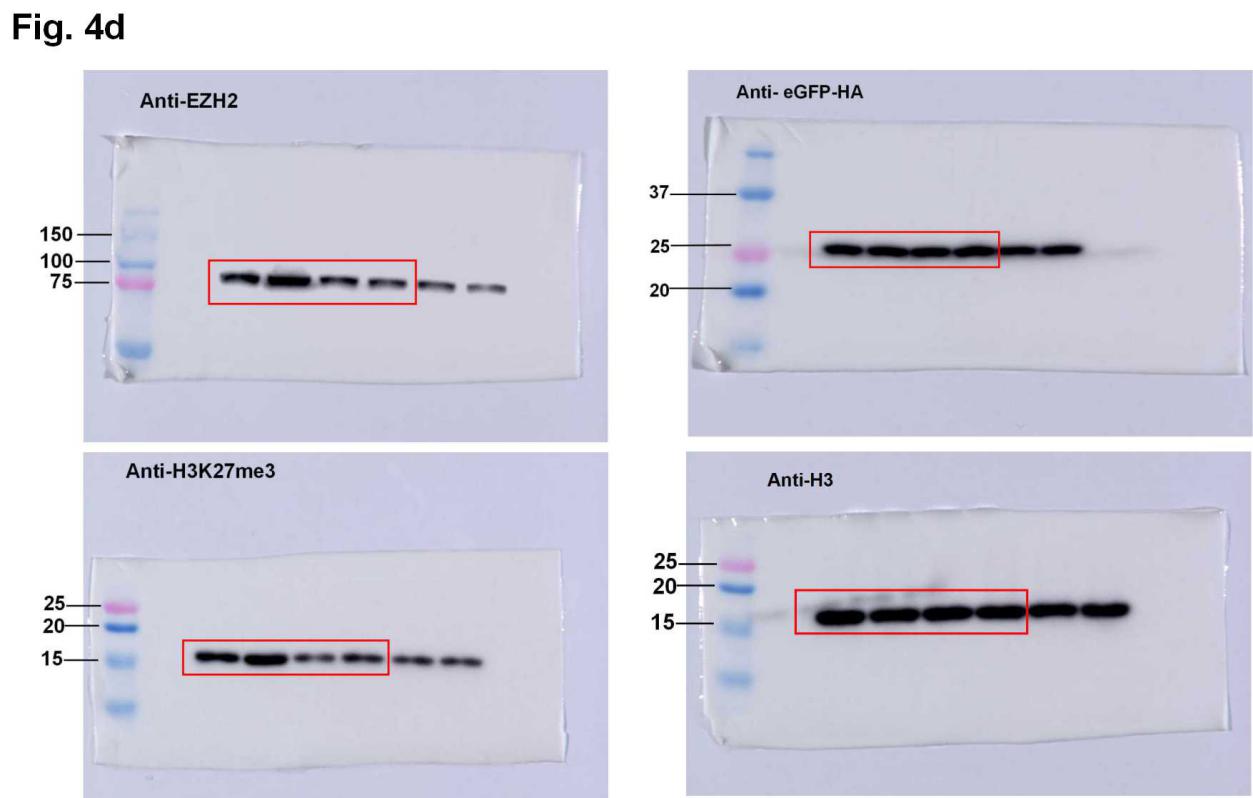


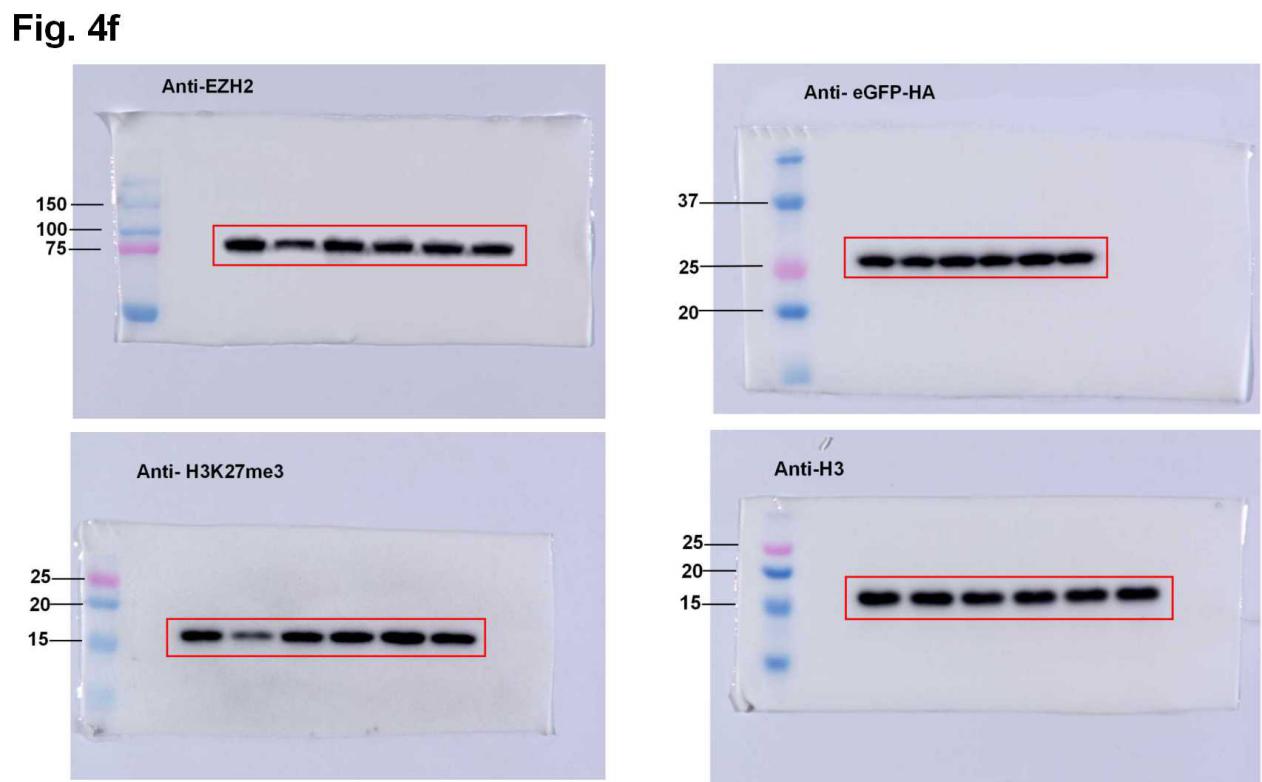


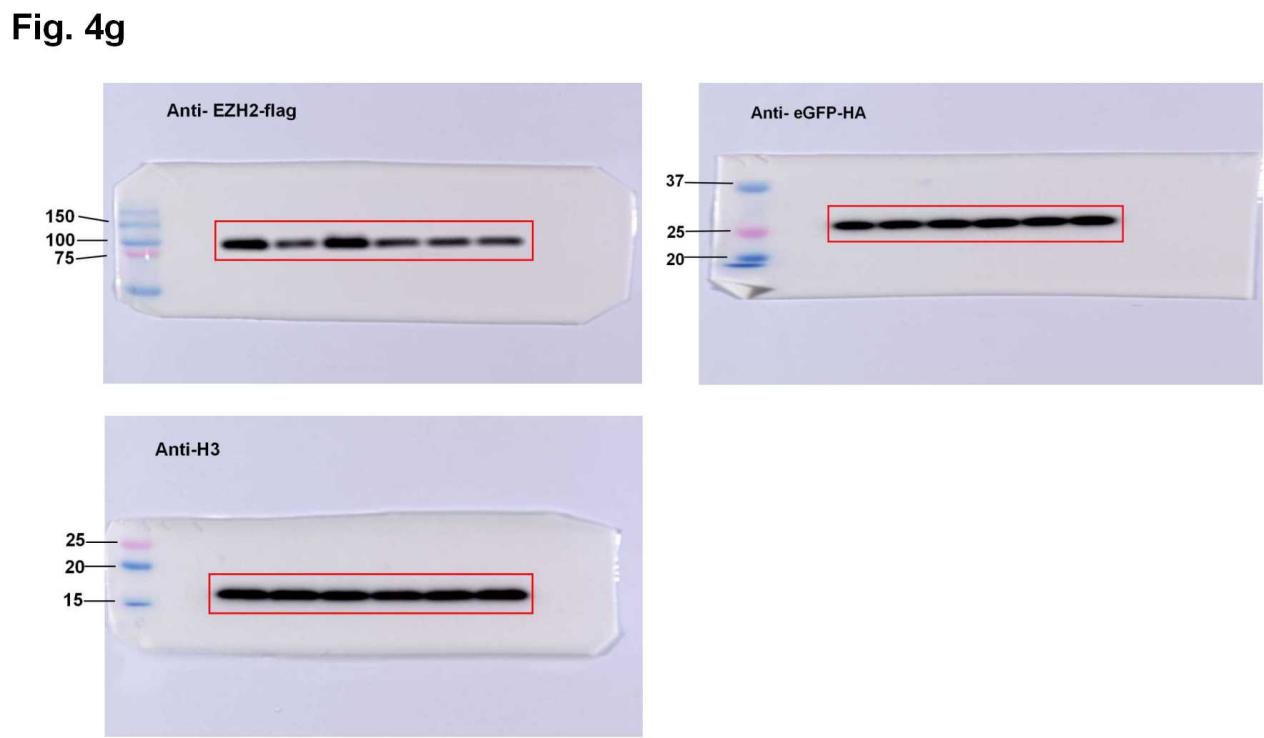


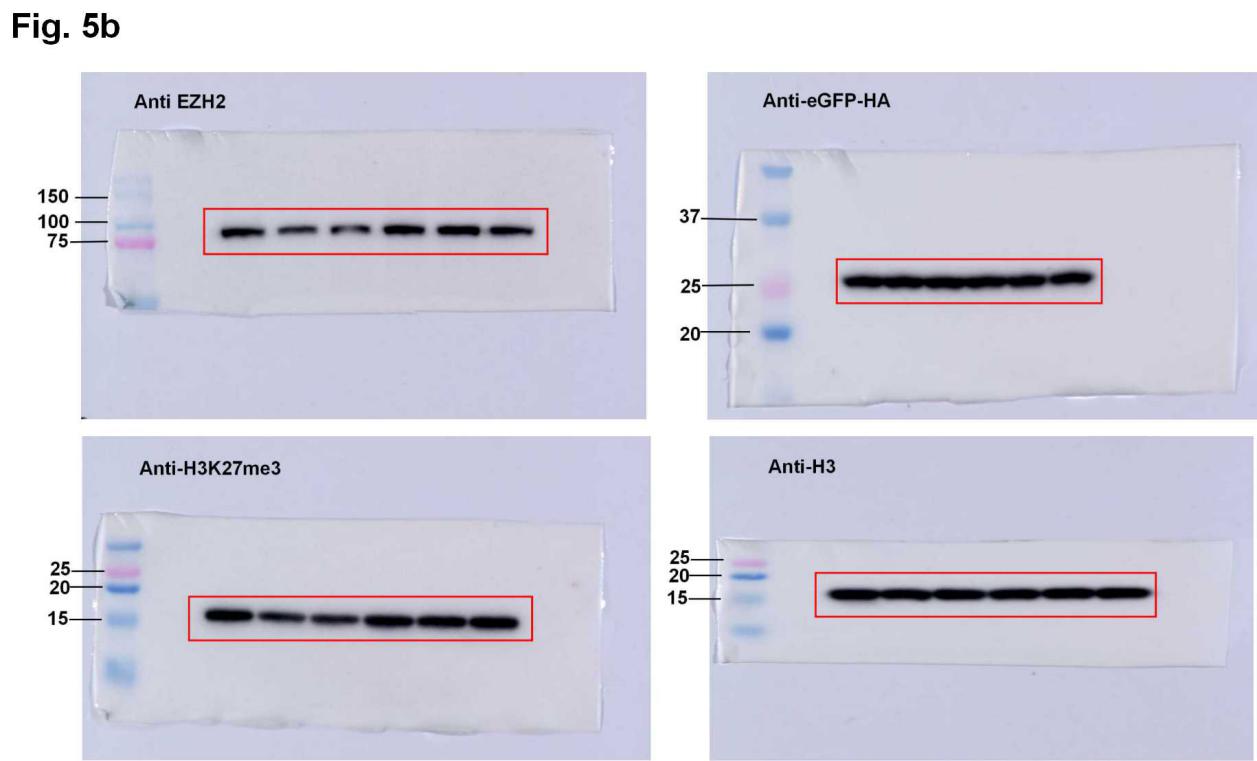


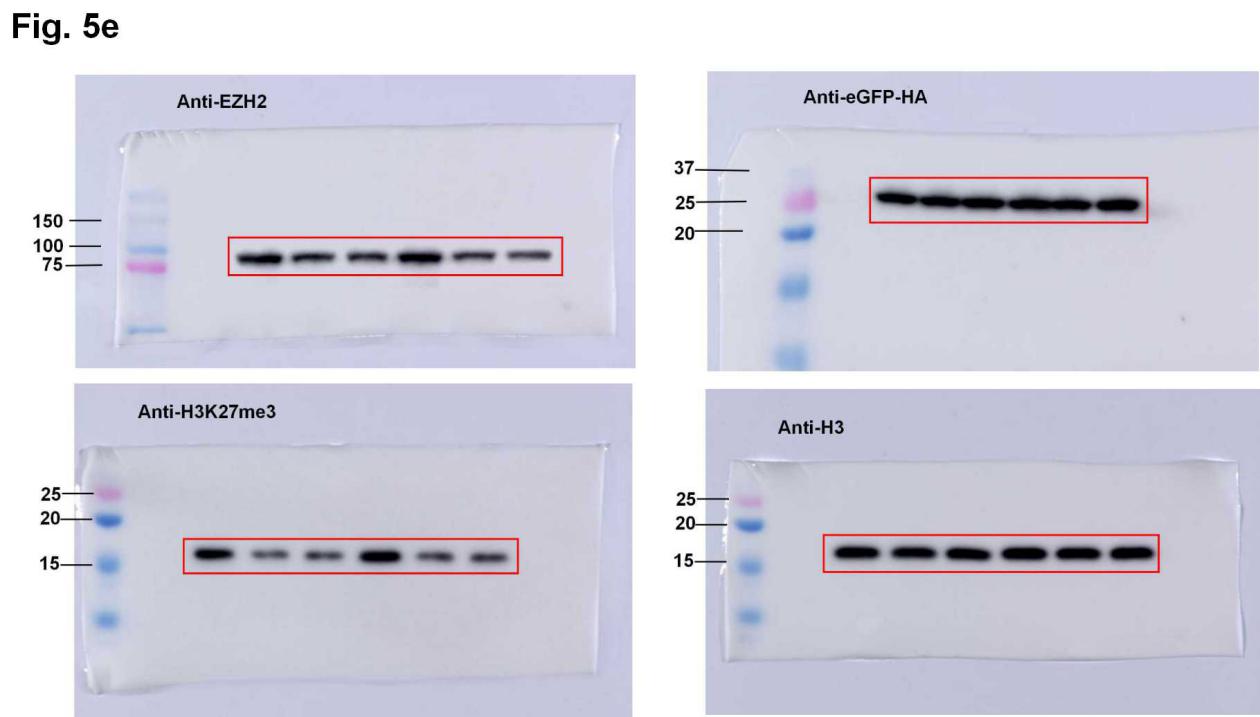


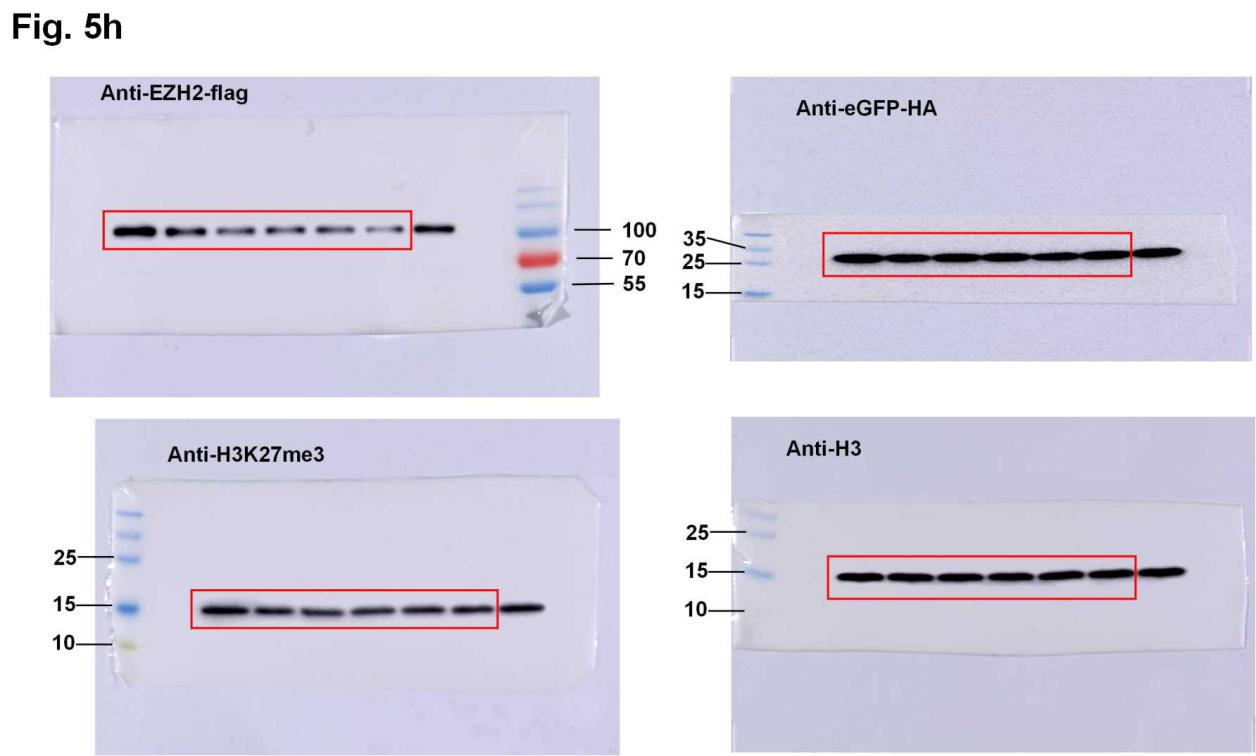


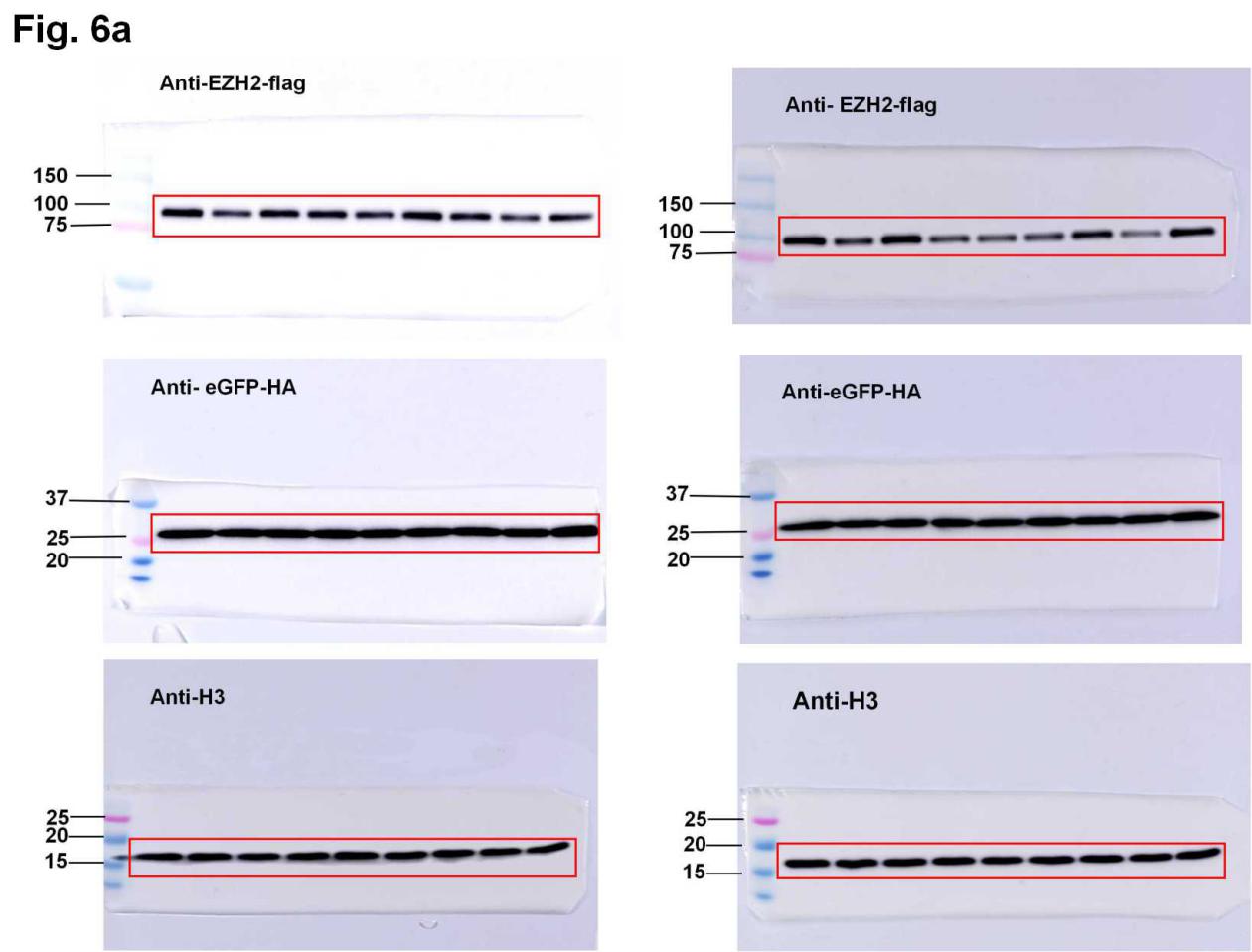


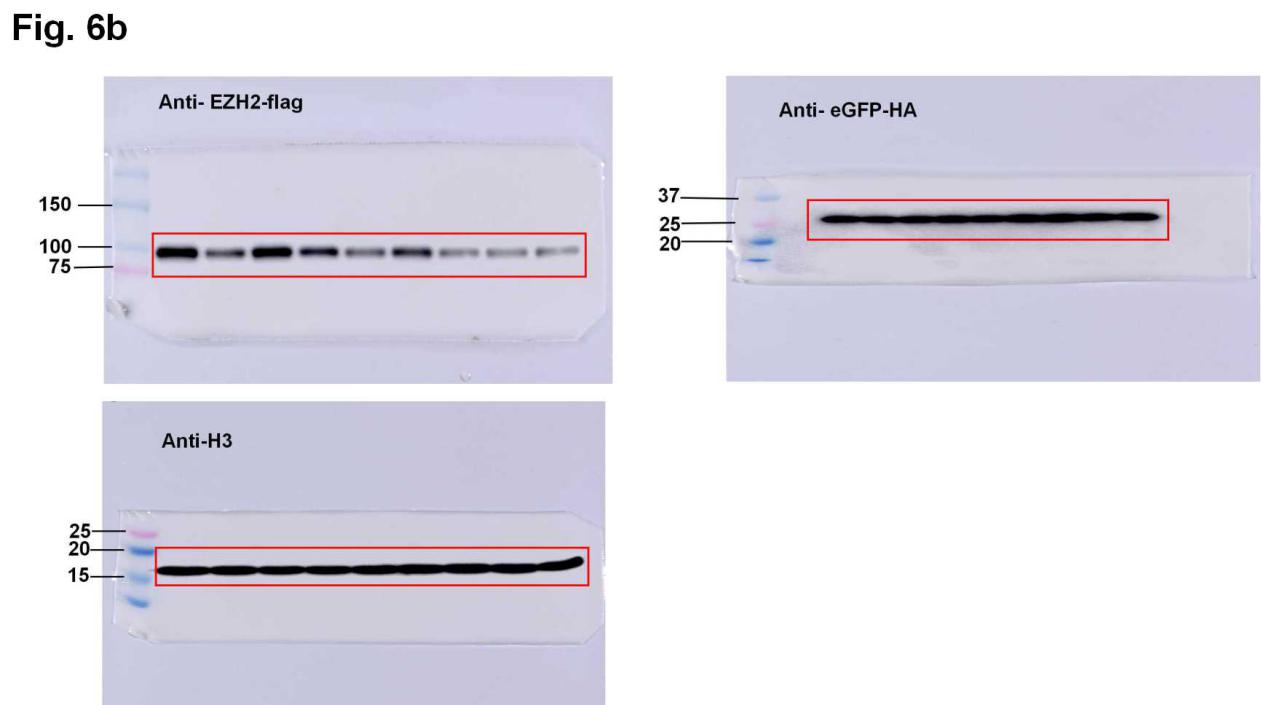


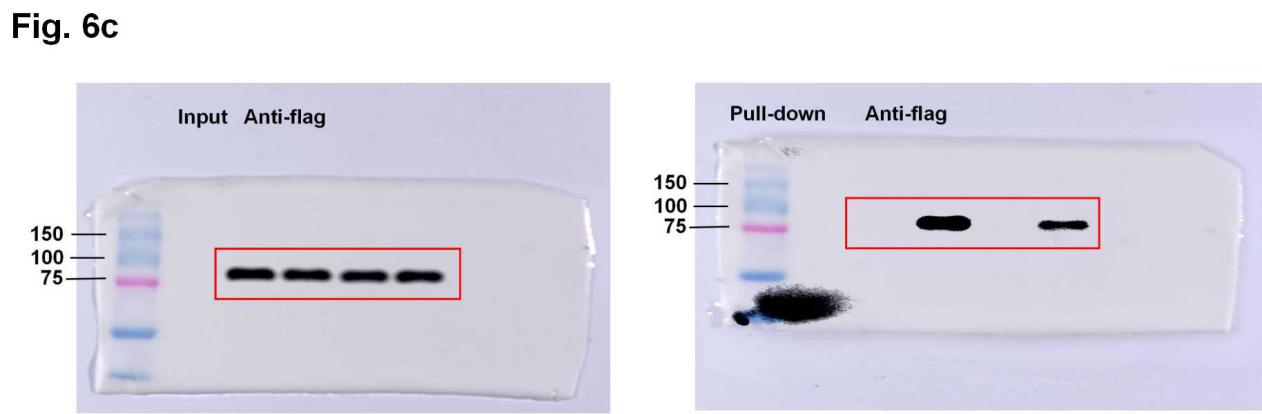


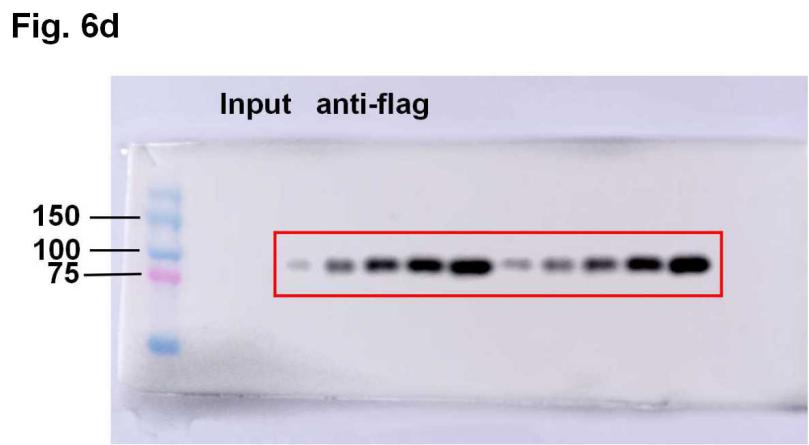


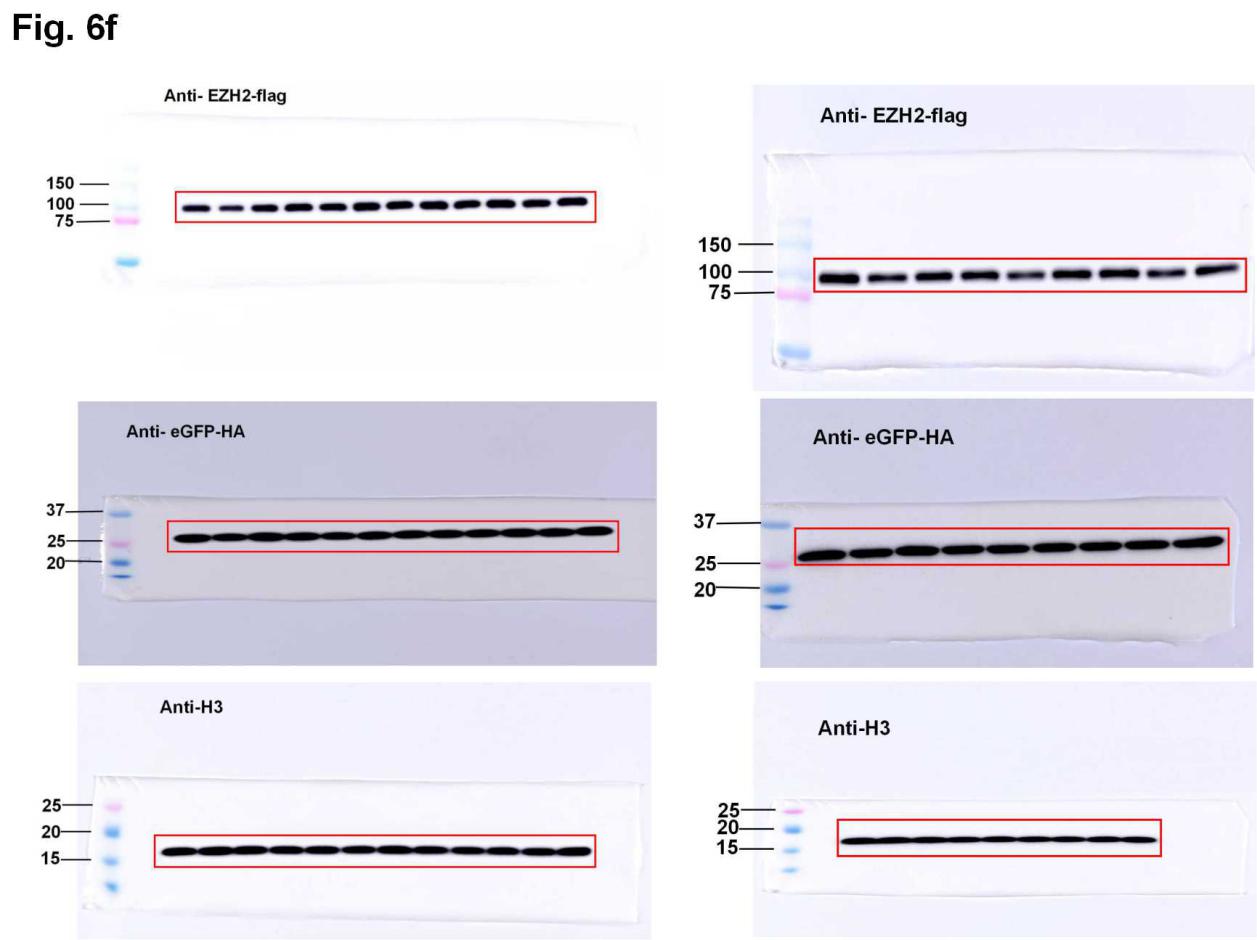


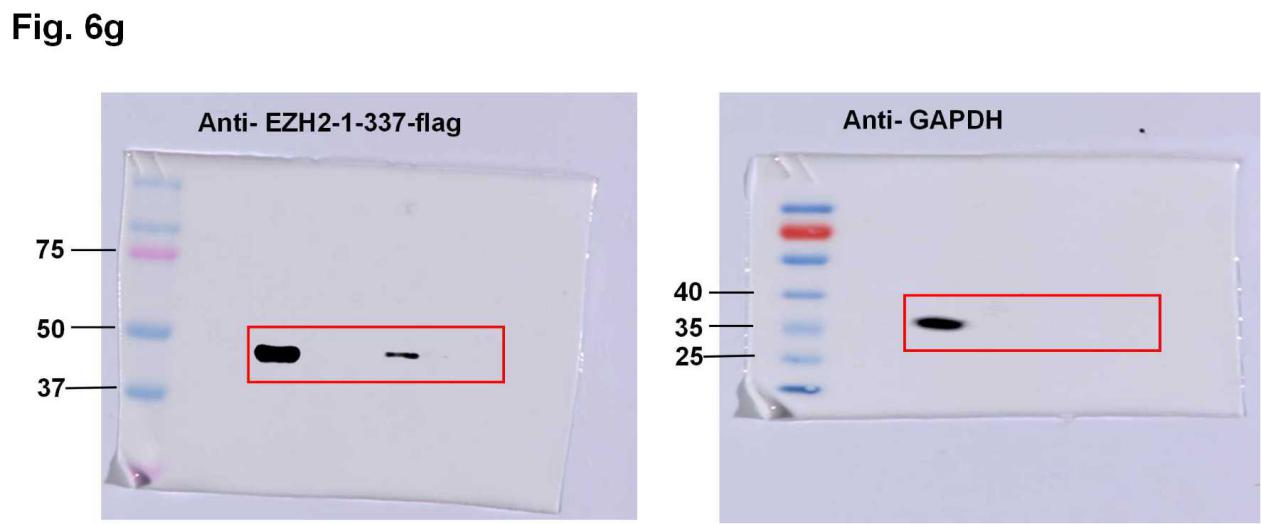


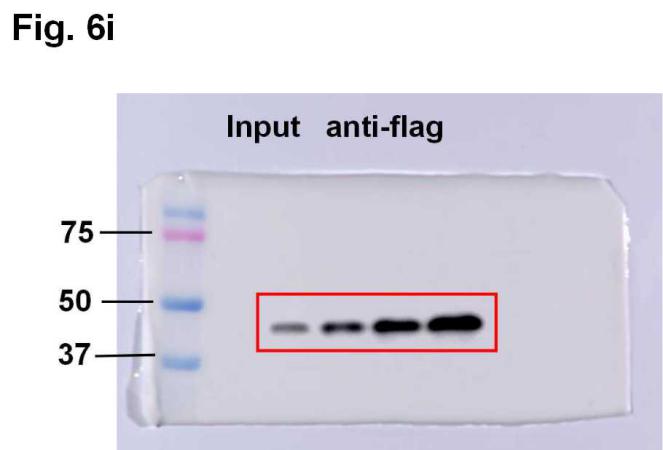


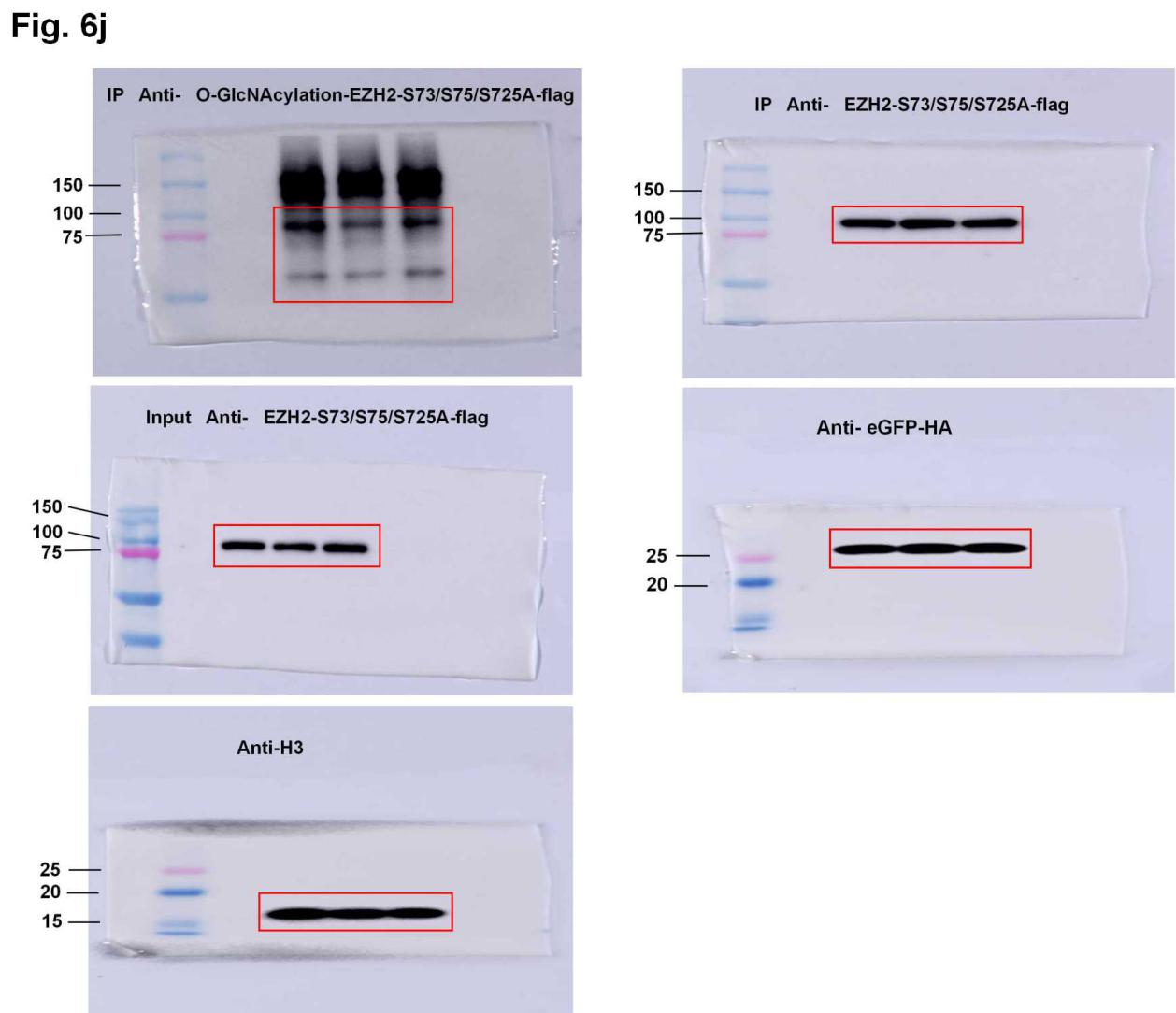


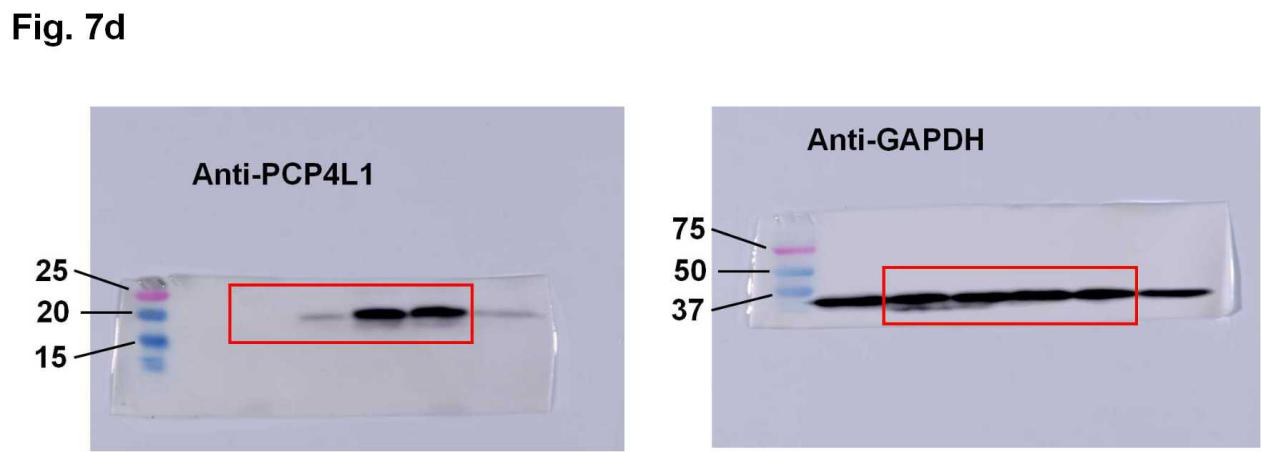


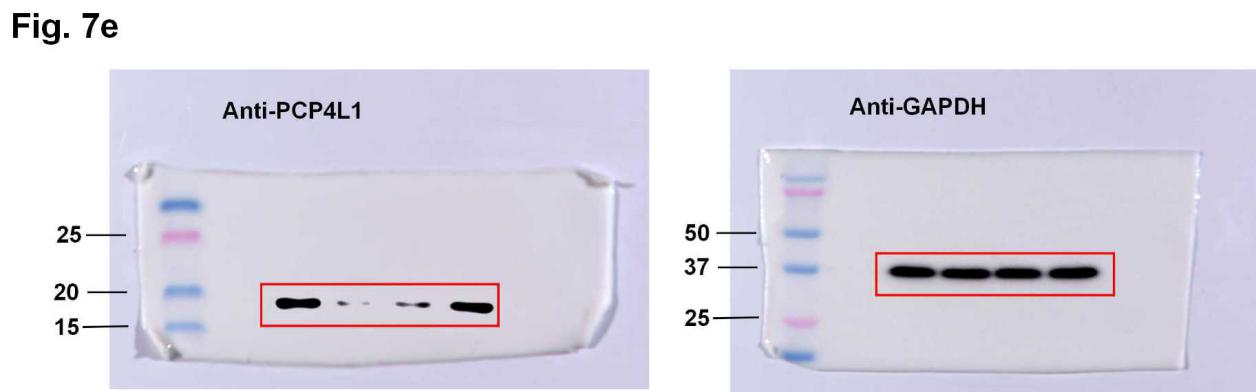


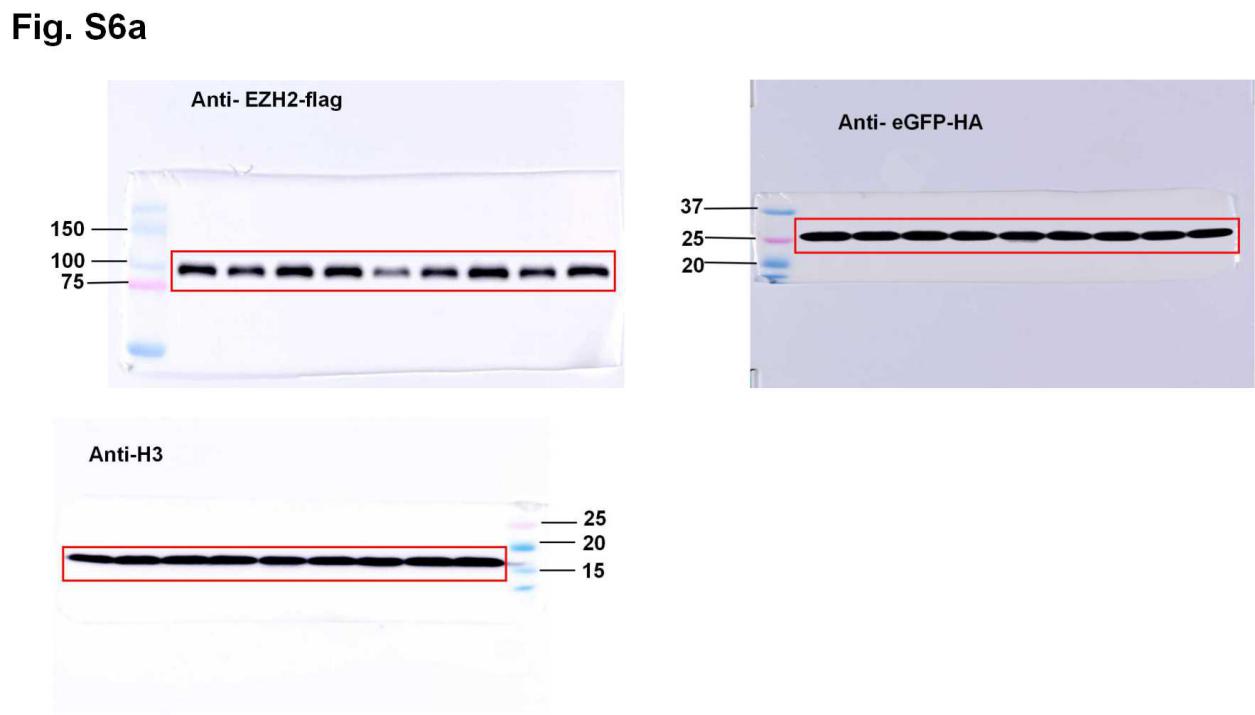


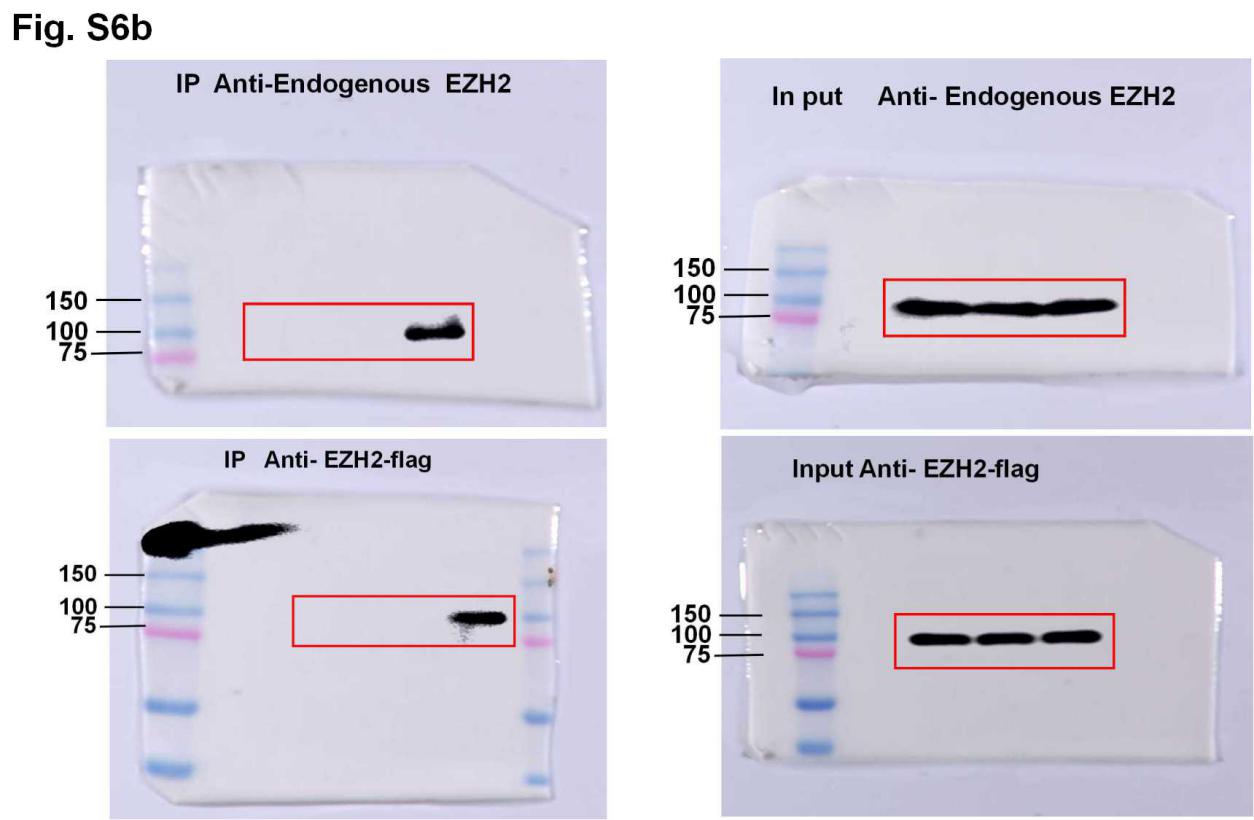


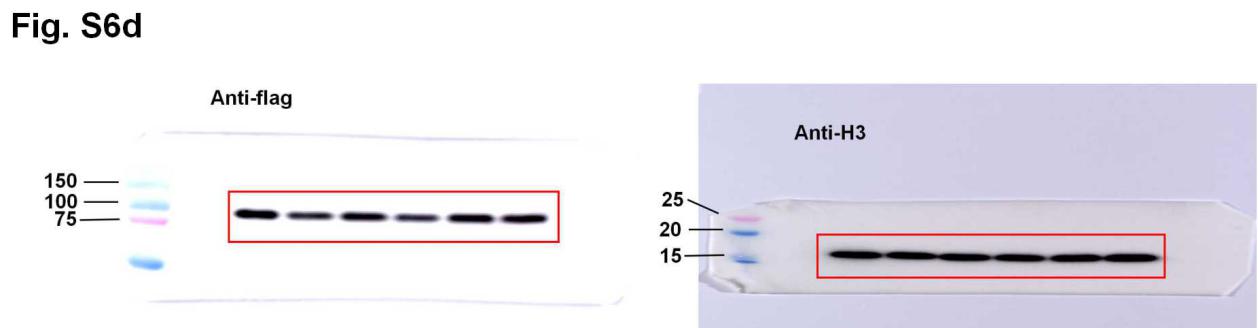


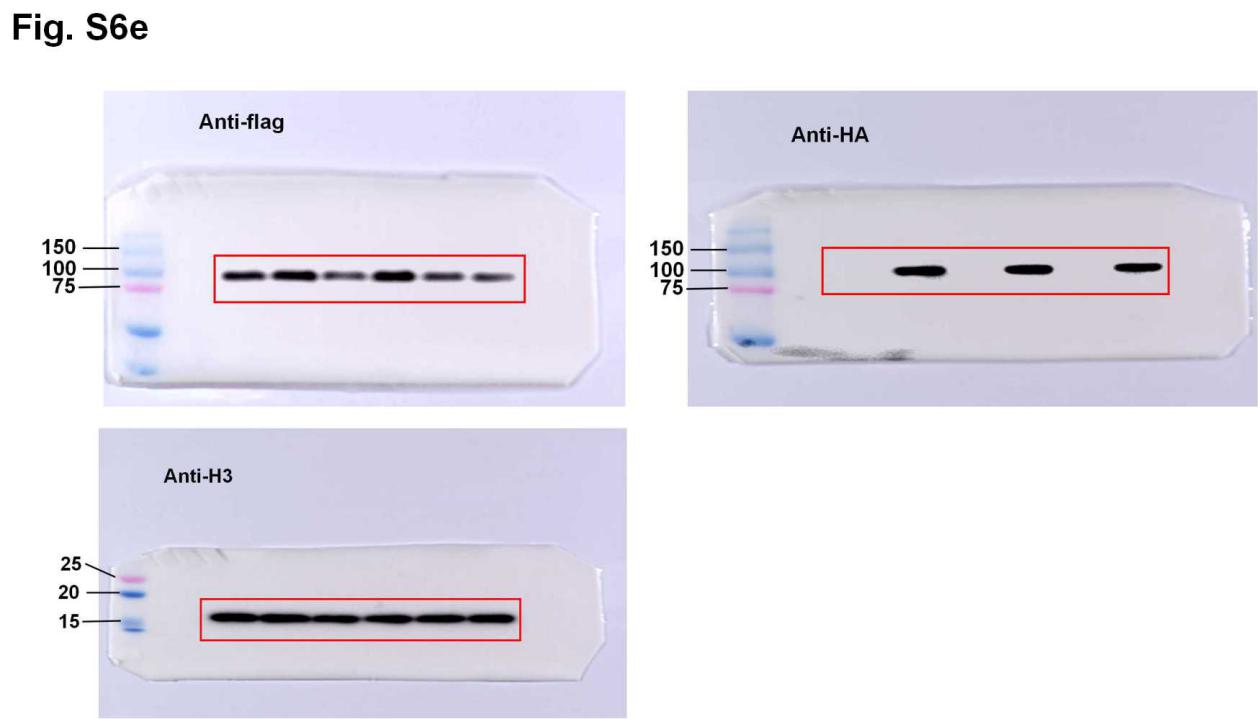

Supplement: Supplementary file 4 — Additional file 4. Complete western blot images of all figures in the manuscript are provided in additional file 4. [file 13059_2020_2150_MOESM4_ESM.docx]
