# Supplementary material for: A novel antiviral lncRNA, EDAL, shields a T309 O-GlcNAcylation site to promote EZH2 lysosomal degradation
Source: Genome Biol. 2020 Sep 1;21:228. doi: 10.1186/s13059-020-02150-9 (PMC7465408; doi:10.1186/s13059-020-02150-9)
Supplement: Supplementary file 5 — Additional file 5. Review history. [file 13059_2020_2150_MOESM5_ESM.docx]

**Review history**

**First round of review**

**Reviewer 1**

In this manuscript, Sui et al. identified a novel lncRNA EDAL that could inhibit viral replication in vitro and in vivo. Interestingly,  EDAL binds EZH2 via an 18-nt hairpin region, and such binding could block the O-GlcNAcylation modification of T309 in EZH2 and promote its degradation via the lysosomal pathway. The EZH2 degradation further reduced the global H3K27me3 level and boosted the transcription of Pcp4l1, a 68aa polypeptide that able to inhibit the replication of neurotropic viruses.  Overall, I am excited about the discovery, and it should be of great interest to the scientific community. However, more experimental evidence is needed to support the critical conclusion of EDAL binding at EZH2.  I would support the publication of this paper if the authors can address the following concerns.
Major points:
1. The authors found that EDAL could form four sub-structures and demonstrated that only EDAL-1 could prevent viral replication. Whether these RNA secondary structures are real is unclear if only based on predictions using RNAstructure software, which usually gives many alternative structures. Mapping the whole secondary structure of EDAL-1 is not necessary, but I strongly recommend the authors to determine the EDAL-1 structure using T1/V1 footprinting or in-line probing assays.
2. It seems that the authors identified a different region in EZH2 that could bind RNA. Whether authors can compare the binding of EDAL with wild-type or mutant forms of EZH2 using gel-shift assay? The authors can use the proteins deleting either 342-368aa or 305-312aa.
3. Page 17, Line 421-423, the conclusion "EDAL interaction sites on EZH2 are in its N-terminal region (1-337 aa)" needs to be reworded.  The RNA pull-down assay in Fig.6e indicates that EDAL can interact with the N-terminal region of EZH2. However, the authors can not exclude the possibility that EDAL may also bind the 342-368 aa in EZH2.  The authors should include the mutant as a control for comparison.
4. The authors propose that EDAL1 may restrict viral replication by upregulating PCP4L1. The observation is interesting, but whether the antiviral activity of EDAL1 is via PCP4L1 is unclear. I recommend authors to test whether knockdown PCP4L1 in viruses infected N2a cells could compromise the antiviral effects of EDAL.
5. The RNA FISH shown in Fig.S2e is not convincing. The authors can include an additional control such as MALAT1 to prove the FISH assay is working. Additionally, a nuclear/cytoplasmic fractionation and qPCR assay should also be applied to quantify the relative distribution of EDAL in neuronal cells.
Minor points:
1. In Fig. 1k, the last column (kidney) was not displayed properly.
2. In Fig. 2a,h; Fig S4e: the base number for log in the y-axis should be labeled.
3. Page 8, Line 173: 'Bessides RABV' should be 'Besides RABV.'
4. Page 8, Line 181: 'Figure S2d-2g' should be 'Figure S3d-3g.'
5. Fig 4h, Line 1322: 'transfected togethwe' should be 'transfected together.'
6. Fig. 6b legend, Line 1364, 'The potential ... sites ... was mutated' should be 'The potential ... sites ... were mutated.'

**Reviewer 2**

A novel antiviral lncRNA EDAL shields a T309 O-GlcNAcylation site to promote EZH2 lysosomal degradation.

In this paper the authors identify a new lncRNA EZH2 degradation associated lncRNA (EDAL). They show that EDAL is virally inducible and it functions to bind to EZH2 that gets degraded. They identify the region within EDAL that mediates if functions and the region it associates with in EZH2. Secondly, they show that EDAL leads to the increase in Pcp41 transcription. Pcp41 is a 10kDa peptide that inhibits virus replication. Overall this is an interesting and highly mechanistic story. I have concerns about the strength of some of the data. My biggest issue is the nature of overexpression in most of the experiments and how this data can be interpreted given that they are overexpressing the lncRNA on top of the endogenous transcript (using the REVlncRNAs as a control however the endogenous lncRNA is always present).  Specific concerns are outlined below and would need to be addressed before I could recommend publishing the story

Fig.1 and Supplemental Fig.2
-     The authors are describing this new lncRNA for the first time. It would be useful to show the reader the outline of the lncRNA (specific isoform that the authors are looking at). I find Fig.Sup2 very confusing. From the reads from the RNA seq there looks to be exonic structure to the lncRNA. Yet from the RACE they show that it is just one large exon. Which is it really? What is the exact sequence being overexpressed? Are there really no exon/intron junctions? Is there any long-read sequencing of this locus available to provide a better understanding of the isoform structure of this locus?
-     For the qPCR primers used where are they amplifying? Here the authors could test if any are exon spanning if indeed there are exons or not. How many primer sets were used to test qPCRs like those shown in Fig1 d-k
-     The authors just performed PhyloCSF analysis to say this is indeed a lncRNA. Unfortunately, I don't think this is sufficient to make this claim. Other data such as polysome profiling is required to make a claim of no coding potential especially given that EDAL appears to be localizing to the cytosol
-     Localization experiments are very weak. SupFig2E, where are the controls? How do we know the probe is specific? This needs to be performed with siRNA as a control as well as strong positive and negative controls

Fig.2
-     Knockdown as shown in SupFig4 is quite modest (50%)
-     I would like to see more than a single siRNA being used. Even better would be the use of CRISPRi to knockdown the endogenous gene and show these effects 

Fig.2C
-     how does the overexpression of EDAL rescue this effect? Does the siRNA not target the sequence within EDAL in the overexpression construct?
-     The authors say that EDAL expression is MOI and time dependent. It is really time dependent? Overtime the virus is replicating, so is it just from accumulation of virus?

Fig2G and Fig.3
-     It wasn't clear to me how informative the experiment outlined in Fig.2G is. The authors place the lncRNA into the viral genome however this is a host lncRNA and all of its impacts on the lncRNA will occur from the host cell becoming infected with the virus. While the data looks interesting I think the motivation and insight gained from this experiment needs to be better explained to the reader. If the authors want to show that EDAL impacts RABV in vivo they would need to generate a knockout mouse and infect with RABV.

Fig.4
-     All the mechanistic work is very interesting however it is all dependent on the overexpression system. It would be nice to confirm some of the findings with the endogenous genes

Fig.5
-     It is very interesting that the authors pin points the antiviral activity to 56nt portion of the lncRNA. Can the authors speculate on why the lncRNA is so long if only 56nt are needed?
-     URL in figure legend is broken

Sup5.
-     Fig Sup.5b, the data showing the interaction with EZH2 is very weak
-     As currently outlined and discussed SupFig.5d and e are. The findings should be rewritten with more clarity to enable the read to interpret the data.

Fig.6
-     These experiments involve overexpressing EDAL using REVEDAL a however how is the readout not impacted by the expression of the endogenous EDAL?  

-     The use of the gsk126 is very exciting from a clinical perspective. Can it be administered post infection? Could an in vivo experiment be carried out to give mice gsk126 at day 4 following infection and will this be protective? Can this molecule cross the blood brain barrier?

-     RNA-seq of differentially expressed genes and ChIP-seq of H3K27me3 was performed to identify genes that are regulated specifically through the EDAL-EZH2 pathway 6 genes were then tested…how were these genes selected? (line 490-491)

-     The data on Pcp4l1 is quite clear, however how this protein is antiviral is not clear? This causes the end of the story to appear open ended. Even providing more discussion could be useful.

Materials and Methods: Need to add additional information to methods to ensure another researcher could try to replicate results. For example, how much siRNA was transfected? How much probe was used in FISH experiments

Minor comments:
-     Line 80 "none" I assume you mean known
-     Figure 1k is cut off
-     Line 375 specifically - spelling error
-     Syntax of line 471 is confusing

Note for all WBs:
-     It is great that ratios are written under each band, however are these WBs performed in triplicate? If so, can standard deviations be shown as well next to each ratio?

**Authors’ response to reviewers**

Reviewer reports:
Reviewer #1: In this manuscript, Sui et al. identified a novel lncRNA EDAL that could inhibit viral replication in vitro and in vivo. Interestingly, EDAL binds EZH2 via an 18-nt hairpin region, and such binding could block the O-GlcNAcylation modification of T309 in EZH2 and promote its degradation via the lysosomal pathway. The EZH2 degradation further reduced the global H3K27me3 level and boosted the transcription of Pcp4l1, a 68aa polypeptide that able to inhibit the replication of neurotropic viruses. Overall, I am excited about the discovery, and it should be of great interest to the scientific community. However, more experimental evidence is needed to support the critical conclusion of EDAL binding at EZH2. I would support the publication of this paper if the authors can address the following concerns.

Major points:
1 The authors found that EDAL could form four sub-structures and demonstrated that only EDAL-1 could prevent viral replication. Whether these RNA secondary structures are real is unclear if only based on predictions using RNAstructure software, which usually gives many alternative structures. Mapping the whole secondary structure of EDAL-1 is not necessary, but I strongly recommend the authors to determine the EDAL-1 structure using T1/V1 footprinting or in-line probing assays.
*Response: We thank the reviewer’s suggestion! Considering the reliability of RNAstructure software, it is more convincible to confirm the predicted structure by utilizing the traditional biochemistry methods. Due to the impact of COVID-19 on international transportation, we are facing a big trouble in importing α-32P ATP, which is required for T1/V1 footprinting or in-line probing assays. Alternatively, we performed micrococcal nuclease footprinting sequencing to analyze the secondary structures of EDAL-1 and EDAL-98-153 according to the recent reports (1, 2). Briefly, we in vitro transcribed these two RNA molecules by T7 RNA polymerase and probed their secondary structures by limited enzymatic digestion using micrococcal ribonuclease, an endo-exonuclease that preferentially digests single-stranded nucleic acids at the 5' side of A or T. The digested RNA fragments were ligated with 5’ and 3’ adaptors followed by cDNA library preparation and Illumina sequencing. After mapping on the corresponding RNA molecule sequence, we recovered both the 5’-end and 3’-end nucleotide signals representing the cleavage site from mapped reads. Plotting the cleavage signals showed that ribonuclease probing results were primarily in good accordance with the predicted structures of both molecules and the corresponding results were shown in Figure S5 and Table S1.

References:
1) Mailler E, Paillart JC, Marquet R, Smyth RP, Vivet-Boudou V: The evolution of RNA structural probing methods: From gels to next-generation sequencing. Wiley Interdiscip Rev RNA 2019, 10:e1518.
2) Kwok CK, Tang Y, Assmann SM, Bevilacqua PC: The RNA structurome: transcriptome-wide structure probing with next-generation sequencing. Trends Biochem Sci 2015, 40:221-232.*

2 It seems that the authors identified a different region in EZH2 that could bind RNA. Whether authors can compare the binding of EDAL with wild-type or mutant forms of EZH2 using gel-shift assay? The authors can use the proteins deleting either 342-368aa or 305-312aa.
*Response: Thanks for the reviewer’s comments! Previous studies have demonstrated that there is a konwn RNA binding domain in 343-368 aa of human EZH2, which can binding many different lncRNAs with limitted specificity, and this corresponding region in murine EZH2 was 338-364 aa. According to the reviewer suggestion, we constructed murine EZH2 deleting 338-364 aa (EZH2-▲338-364) and then compared the binding ability of EDAL with wt EZH2 and EZH2-▲338-364 by using RNA pull-down and gel-shift assays. After deleting 338-364 aa, we found that EZH2-▲338-364 still bind with EDAL by RNA pull-down assay and the new data were shown in Fig.6c. Consistently, the gel-shift assay results also confirmed that EZH2-▲338-364 could bind with EDAL, which is shown in Fig.6d. These new data support that EDAL binds with a different region besides EZH2 338-364 aa.*

3 Page 17, Line 421-423, the conclusion "EDAL interaction sites on EZH2 are in its N-terminal region (1-337 aa)" needs to be reworded. The RNA pull-down assay in Fig.6e indicates that EDAL can interact with the N-terminal region of EZH2. However, the authors can not exclude the possibility that EDAL may also bind the 342-368 aa in EZH2. The authors should include the mutant as a control for comparison.
*Response: Agreeing with the reviewer’s suggestion, we have compared the binding of EDAL with wt EZH2 and EZH2-▲338-364 by using RNA pull-down assays. After deleting 338-364 aa, we found that EZH2-▲338-364 still bind with EDAL by RNA pull-down and gel-shift assays (Fig.6c, d). Our data suggest that EZH2 1-337 aa also can bind with EDAL besides 338-364 aa. To be noted, EZH2 338-364 aa has been recognized as a RNA binding domain and this region can unspecific bind with different RNA (1, 2). As suggested, we modified the original claim of "EDAL interaction sites on EZH2 are in its N-terminal region (1-337 aa)" into"EDAL could binds EZH2 N-terminal region (1-337 aa)" in line 461-462.

Reference:
1) Davidovich C, Wang X, CifuentesRojas C, Goodrich KJ, Gooding AR, Lee JT, Cech TR: Towards a Consensus on the Binding Specificity and Promiscuity of PRC2 for RNA. Molecular Cell 2015, 57:552-558.
2) Kaneko S, Li G, Son J, Xu CF, Margueron R, Neubert TA, Reinberg D: Phosphorylation of the PRC2 component Ezh2 is cell cycle-regulated and up-regulates its binding to ncRNA. Genes Dev 2010, 24:2615-2620.*

4 The authors propose that EDAL1 may restrict viral replication by upregulating PCP4L1. The observation is interesting, but whether the antiviral activity of EDAL1 is via PCP4L1 is unclear. I recommend authors to test whether knockdown PCP4L1 in viruses infected N2a cells could compromise the antiviral effects of EDAL.
*Response: According to the reviewer’s suggestion, we have knocked down PCP4L1 via siRNA (Fig. 7e, f) and then overexpress EDAL in N2a cells. The results show that EDAL overexpression have no obvious effect on RABV replication in PCP4L1 knockdown N2a cells (Fig. 7g). These new data support that EDAL restricts viruses via PCP4L1.*

5 The RNA FISH shown in Fig.S2e is not convincing. The authors can include an additional control such as MALAT1 to prove the FISH assay is working. Additionally, a nuclear/cytoplasmic fractionation and qPCR assay should also be applied to quantify the relative distribution of EDAL in neuronal cells.
*Response: Agreeing with the reviewer’s suggestion, we have repeated the FISH assay and included another cytoplasmic 18S rRNA as a positive control (Fig. S2f). Additionally, according to the reviewer’s suggestion, we also performed a nuclear/cytoplasmic fractionation and qPCR assay. 18S or β-actin was used as the cytoplasmic positive control and lncRNA Malat1 as the nuclear positive control (shown in Fig. 1d). Both of the results demonstrate that EDAL is mainly located in cytoplasm.*

Minor points:
1 In Fig. 1k, the last column (kidney) was not displayed properly.
*Response: Thanks for the comment! We have fixed it.*

2 In Fig. 2a,h; Fig S4e: the base number for log in the y-axis should be labeled.
*Response: According to the suggestion, we have labeled the base number for log in the y-axis.*

3 Page 8, Line 173: 'Bessides RABV' should be 'Besides RABV.'
*Response: We have fixed the typo in line 187*.

4 Page 8, Line 181: 'Figure S2d-2g' should be 'Figure S3d-3g.'
*Response: We have corrected the wrong labeling in line 195.*

5 Fig 4h, Line 1322: 'transfected togethwe' should be 'transfected together.'
*Response: We have fixed the typo in line 1480.*

6 Fig. 6b legend, Line 1364, 'The potential ... sites ... was mutated' should be 'The potential ... sites ... were mutated.'
*Response: We have fixed the grammar mistake in line 1522.*


Reviewer #2: A novel antiviral lncRNA EDAL shields a T309 O-GlcNAcylation site to promote EZH2 lysosomal degradation.

In this paper the authors identify a new lncRNA EZH2 degradation associated lncRNA (EDAL). They show that EDAL is virally inducible and it functions to bind to EZH2 that gets degraded. They identify the region within EDAL that mediates if functions and the region it associates with in EZH2. Secondly, they show that EDAL leads to the increase in Pcp41 transcription. Pcp41 is a 10kDa peptide that inhibits virus replication. Overall this is an interesting and highly mechanistic story. I have concerns about the strength of some of the data. My biggest issue is the nature of overexpression in most of the experiments and how this data can be interpreted given that they are overexpressing the lncRNA on top of the endogenous transcript (using the REVlncRNAs as a control however the endogenous lncRNA is always present). Specific concerns are outlined below and would need to be addressed before I could recommend publishing the story

Fig.1 and Supplemental Fig.2
- The authors are describing this new lncRNA for the first time. It would be useful to show the reader the outline of the lncRNA (specific isoform that the authors are looking at). I find Fig.Sup2 very confusing. From the reads from the RNA seq there looks to be exonic structure to the lncRNA. Yet from the RACE they show that it is just one large exon. Which is it really? What is the exact sequence being overexpressed? Are there really no exon/intron junctions? Is there any long-read sequencing of this locus available to provide a better understanding of the isoform structure of this locus?
*Response: Thanks for the comment! According to the suggestion, we re-made Fig.S2b and make it clearer for readers. This novel lncRNA, EDAL, was predicted by cufflink software (1) with default parameters (the detailed methods were described in supplemental information). From the prediction results, only one exon was detected for this lncRNA. The RACE assay also supported this prediction result. The discontinuous distribution of RNA-seq reads may be attributed to the insufficient RNA-seq reads. We also aligned the long sequences obtained by RACE assay to the lncRNA locus. Mismatches or deletions/insertions were not detected from the alignment. The overlapped part and the extended parts between 5’ RACE and 3’ RACE sequences indicated that this lncRNA has only one exon (Figure S2b). The exact sequence of EDAL expressed in this study is listed in Table S4.*

*Reference:
1) Trapnell C, Roberts A, Goff L, Pertea G, Kim D, Kelley DR, Pimentel H, Salzberg SL, Rinn JL, Pachter L: Differential gene and transcript expression analysis of RNA-seq experiments with TopHat and Cufflinks. Nature Protocols 2012, 7:562.*

- For the qPCR primers used where are they amplifying? Here the authors could test if any are exon spanning if indeed there are exons or not. How many primer sets were used to test qPCRs like those shown in Fig1 d-k
*Response: We used two 3’RACE primers and one 5’RACE primer to perform RACE experiments. The exact RACE primer sites were shown in Figure S2b. There is a large overlap region between 3’RACE and 5’RACE primers. Based on our 5’RACE and 3’ RACE data (Figure S2b), we did not find any spanning exon in EADL, so we used 1 primer set in qPCR assay. The exact position of the primer sites in EDAL is shown in Figure S2b and Table S4.*

- The authors just performed PhyloCSF analysis to say this is indeed a lncRNA. Unfortunately, I don't think this is sufficient to make this claim. Other data such as polysome profiling is required to make a claim of no coding potential especially given that EDAL appears to be localizing to the cytosol
*Response: Thanks for the suggestion! Agreeing with the comment, we isolated the ribosome-RNA complex by using size exclusion chromatography with MicroSpin S-400 HR Columns and extracted the ribosome binding RNA. Then we quantified the level of EDAL as well as a well-known lncRNA Malat1 and two coding RNAs Dennd1b and Crebrf by using qPCR. To be noted, Dennd1b and Crebrf dispalyed the comparable basal level with EDAL in N2a cells. The results showed that the level of ribosome-binding EDAL was far more less than lncRNA Malat1 and two coding RNAs, Dennd1b and Crebrf, indicating its non-coding characteristics (Additional file 2: Figure S2d).*

- Localization experiments are very weak. SupFig2E, where are the controls? How do we know the probe is specific? This needs to be performed with siRNA as a control as well as strong positive and negative controls
*Response: Thanks for the suggestion! We have repeated FISH experiment and included 18S rRNA as a cytoplasmic control (Fig. S2f). To make sure that the probe is specific, FISH were also performed post EDAL overexpression or silencing as shown in Fig. S4a and S4b, respectively. Additionally, a nuclear/ cytoplasmic fractionation and qPCR assay also performed. 18S rRNA and β-actin were measured as the cytoplasmic positive control, and lncRNA Malat1 was tested as the nuclear positive control (Fig. 1d). These new data supported that EDAL was mainly located in cytoplasm.*

Fig.2
- Knockdown as shown in SupFig4 is quite modest (50%)
- I would like to see more than a single siRNA being used. Even better would be the use of CRISPRi to knockdown the endogenous gene and show these effects
*Response: Thanks for the suggestions! We repeated this experiment by using three different sets of siRNA and optimized the transfection condition. The knock-down efficiency of different siRNA was shown in Fig. S4b. We re-evaluated the effect of EDAL silencing on RABV proliferation (Fig. 2b). CRISPRi may be a better approach to confirm this finding, and we will try to introduce this cutting-edge technique in our future study.*

Fig.2C
- how does the overexpression of EDAL rescue this effect? Does the siRNA not target the sequence within EDAL in the overexpression construct?
*Response: This experiment is aimed to prove that siRNA for EDAL is not off-target. Firstly, we transfected siRNA into the cells and the transfected siRNA is mainly targeted to the endogenous EDAL. After 24 hours, the quantity of siRNA in the cells was very limited and then we transfected another EDAL-expressing plasmid into cells. After DEAL overexpression, we found that the virus titer recovered to that transfected with control siRNA and empty vector, indicating that siRNA for EDAL is not off-target.*

- The authors say that EDAL expression is MOI and time dependent. It is really time dependent? Overtime the virus is replicating, so is it just from accumulation of virus?
*Response: Agreeing with the comment, we intent to mention that the up-regulation of EDAL is correlated with virus replication. We have modified the description in line 186-192 to avoid any confusion.*

Fig2G and Fig.3
- It wasn't clear to me how informative the experiment outlined in Fig.2G is. The authors place the lncRNA into the viral genome however this is a host lncRNA and all of its impacts on the lncRNA will occur from the host cell becoming infected with the virus. While the data looks interesting I think the motivation and insight gained from this experiment needs to be better explained to the reader. If the authors want to show that EDAL impacts RABV in vivo they would need to generate a knockout mouse and infect with RABV.
*Response: To test the impacts of EDAL on virus pathogenicity in vivo, we planned two strategies: (1) to insert EDAL into the genome of RABV and overexpress it with virus replication; (2) to construct EDAL knockout mice. Actually, plan 2 is our first choice. Unfortunately, we found deficiency of EDAL compromised the mouse breeding. Thus, we have to construct the recombinant RABV expressing EDAL and test the impact of EDAL on RABV pathogenicity in vivo. We mentioned the purpose for construction of these recombinant viruses in line 231-232.*

Fig.4
- All the mechanistic work is very interesting however it is all dependent on the overexpression system. It would be nice to confirm some of the findings with the endogenous genes
*Response: Thanks for the suggestion! We transfected siEDAL into N2a cells to knockdown the endogenous EDAL, and the results showed that silencing EDAL enhanced the endogenous EZH2 level (Fig.4d) and restricted RABV replication (Fig.2b). In addition, we added the new data of silencing endogenous PCP4L1 on RABV replication (Fig. 7e-g) in this revised version.*

Fig.5
- It is very interesting that the authors pin points the antiviral activity to 56nt portion of the lncRNA. Can the authors speculate on why the lncRNA is so long if only 56nt are needed?
*Response: That is really a good question! lncRNAs play their roles in regulating gene expression via their special secondary structures and many other previous studies support this point (1). In our study, we found that the special hairpin structure of EDAL in 98-153 nt is critical to be associated with EZH2. However, to explain how and why this special structure of EDAL contacts with EZH2 depends on deciphering the crystal structure of EZH2 binding with EDAL in the future.*

Reference:
1) Cech TR, Steitz JA: The noncoding RNA revolution-trashing old rules to forge new ones. Cell 2014, 157:77-94.

- URL in figure legend is broken
*Response: We have fixed this problem in line 1486.*

Sup5.
- Fig Sup.5b, the data showing the interaction with EZH2 is very weak
*Response: We repeated this experiment by optimizing the antibody concentration. The new data clearly confirmed the O-GlcNAcylation modification of murine EZH2 (shown in Fig. S6b).*

- As currently outlined and discussed SupFig.5d and e are. The findings should be rewritten with more clarity to enable the read to interpret the data.
*Response: Agreeing with the suggestion, we modified the descriptions related to Sup Fig.5d and 5e (Sup Fig.6d and 6e in this revised reversion) in line 478-489, and tried to make it more clearly to readers.*

Fig.6
- These experiments involve overexpressing EDAL using REVEDAL a however how is the readout not impacted by the expression of the endogenous EDAL?
*Response: The reverse EDAL, revEDAL, was used as a control in these experiments to indicate the specificity effect of EDAL on EZH2. According to the qPCR results shown in Fig. S4a, the endogenous level of EDAL were far more less than overexpressed EDAL, so the effect of endogenous EDAL can be ignored.*

- The use of the gsk126 is very exciting from a clinical perspective. Can it be administered post infection? Could an in vivo experiment be carried out to give mice gsk126 at day 4 following infection and will this be protective? Can this molecule cross the blood brain barrier?
*Response: According to the suggestion, we gave mice gsk126 (50 mg/kg as previously reported (1)) at day 4 post infection with lethal dose of RABV and the survivor ratio was monitored daily for 3 weeks.However, we did not observe enhanced survivor ratio for mice given with gsk126 compared with the control group given with DMSO. Thus we speculate that gsk126 may not pass through BBB. Supporting our results, a previous study showed that gsk126 displayed much less membrane permeability than other EZH2 inhibitors. The proteins of P-glycoprotein (P-gp) and breast cancer resistance protein (BCRP) restricted the brain penetration of gsk126 (2).*

*References:
1) Arbuckle JH, Gardina PJ, Gordon DN, Hickman HD, Yewdell JW, Pierson TC, Myers TG, Kristie TM: Inhibitors of the Histone Methyltransferases EZH2/1 Induce a Potent Antiviral State and Suppress Infection by Diverse Viral Pathogens. Mbio 2017, 8:e01141-01117.
2) Zhang P, de Gooijer MC, Buil LC, Beijnen JH, Li G, van Tellingen O: ABCB1 and ABCG2 restrict the brain penetration of a panel of novel EZH2-Inhibitors. Int J Cancer 2015, 137:2007-2018.*

- RNA-seq of differentially expressed genes and ChIP-seq of H3K27me3 was performed to identify genes that are regulated specifically through the EDAL-EZH2 pathway 6 genes were then tested…how were these genes selected? (line 490-491)
*Response: We performed both RNA-seq and ChIP-seq to select the potential targeted genes. The selected genes must satisfy these two conditions in the same time: (1) the genes were up-regulated post EDAL overexpression; (2) the genes lose H3K27me3 mark. Based on this selection criterion, the top 6 genes were picked up for further investigation. To make it easier to understand for readers, we emphasize these descriptions in line 531-532.*

- The data on Pcp4l1 is quite clear, however how this protein is antiviral is not clear? This causes the end of the story to appear open ended. Even providing more discussion could be useful.
*Response: Thanks for the suggestion! Here, for the first time we identified PCP4L1 as a novel antiviral peptide. In this study, we found that it could limit the replication of multiple viruses. We are still working on the detailed mechanism how it restricts different viruses. As suggested, we have added more information about this protein in line 649-656 of the discussion part.*

Materials and Methods: Need to add additional information to methods to ensure another researcher could try to replicate results. For example, how much siRNA was transfected? How much probe was used in FISH experiments
*Response: According to the reviewer’s suggestion, we have added more detailed information, including the transfection of siRNA (line 821-823) and probes of FISH (line 815), in the method part.*

Minor comments:
- Line 80 "none" I assume you mean known
*Response: Since there are already some reports showing that lncRNAs can regulate the host innate immune response. Here, we tried to focus on lncRNAs targeting on other antiviral mechanism besides innate immunity. Thus, we use “none” in this sentence.*

- Figure 1k is cut off
*Response: We have fixed this panel.*

- Line 375 specifically - spelling error
*Response: We have fixed the typo in line 407.*

- Syntax of line 471 is confusing
*Response: These descriptions have corrected in this version (in line 512-513).*

Note for all WBs:

- It is great that ratios are written under each band, however are these WBs performed in triplicate? If so, can standard deviations be shown as well next to each ratio?
*Response: Thanks for the suggestion! It will be more convincible to show the standard deviations of WB results. However, we used more than 6 panels in Fig.4 and Fig.6, and adding more statistical analysis panels will make the figures too crowd. As suggested, we would like to provide statistical analysis along with WB results in our future study.*

**Second round of review**

**Reviewer 1**

The authors have addressed all my concerns, and I now support the publication in Genome Biology.

**Reviewer 2**

The Authors have done a good job answering my questions. It is interesting that the authors mention they made a KO mouse for EDAL and it is lethal. This is exciting and I wonder if they have a phenotype with a heterozygote mouse. Can they obtain data from a moue with just 50% of the gene?
One other small point is the argument not to include statistics on western blots. This remains a bit of a concern. The authors do not mention in the figures how many times the westerns were performed. Is each blot representative of 3? Also while the argument for not making the figure cluttered is understandable I think there are easy ways to include data from multiple western blots (for example you show intensities and ratios beneath, these numbers could be averaged over multiple experiments and shown with SDs)

**Authors’ response to reviewers**

Reviewer #1: The authors have addressed my concerns, and I now support the publication in Genome Biology.

*Response: Thanks for the comments!*

Reviewer #2: The Authors have done a good job answering my questions. It is interesting that the authors mention they made a KO mouse for EDAL and it is lethal. This is exciting and I wonder if they have a phenotype with a heterozygote mouse. Can they obtain data from a moue with just 50% of the gene?

*Response: In fact, EDAL-/- mice are not lethal but have some breeding problems, even the heterozygote mice. We were suffering the pains to get enough pups for animal experiments. We plan to construct neuron-specific EDAL-/- mice to solve this trouble in our future study.*

One other small point is the argument not to include statistics on western blots. This remains a bit of a concern. The authors do not mention in the figures how many times the westerns were performed. Is each blot representative of 3? Also while the argument for not making the figure cluttered is understandable I think there are easy ways to include data from multiple western blots (for example you show intensities and ratios beneath, these numbers could be averaged over multiple experiments and shown with SDs)

*Response: Thanks again for the suggestion! The Western blots in this study were performed at least twice and some of them were repeated three times. We have added this description in the figure legends in this revised version. As suggested, to make the data more convincible we would like to repeat three times and provide statistical analysis along with Western blot results in our future study.*
